# Stereochemistry of Complex Marine Natural Products by Quantum Mechanical Calculations of NMR Chemical Shifts: Solvent and Conformational Effects on Okadaic Acid

**DOI:** 10.3390/md12010176

**Published:** 2014-01-07

**Authors:** Humberto J. Domínguez, Guillermo D. Crespín, Adrián J. Santiago-Benítez, José A. Gavín, Manuel Norte, José J. Fernández, Antonio Hernández Daranas

**Affiliations:** 1 University Institute of Bio-Organic Chemistry “Antonio González” (CIBICAN), Avda. Astrofísico Francisco Sánchez 2, La Laguna 38206, Spain; E-Mails: hdomrod@ull.es (H.J.D.); guillermo.diaz@ues.edu.sv (G.D.C.); ajsb_88@hotmail.com (A.J.S.-B.); jgavin@ull.es (J.A.G.); mnorte@ull.es (M.N.); jjfercas@ull.es (J.J.F.); 2 Department of Organic Chemistry, Faculty of Pharmacy, Avda. Astrofísico Francisco Sánchez s/n, La Laguna 38206, Spain; 3 School of Chemistry, Faculty of Natural Science and Mathematics, University El Salvador, Final 25, Avda. Norte, San Salvador, El Salvador; 4 Department of Chemical Engineering and Pharmaceutical Technology, Faculty of Pharmacy, Avda. Astrofísico Francisco Sánchez s/n, La Laguna 38206, Spain

**Keywords:** quantum mechanical calculations, nuclear magnetic resonance, chemical shifts, marine toxin, structure determination, stereochemistry

## Abstract

Marine organisms are an increasingly important source of novel metabolites, some of which have already inspired or become new drugs. In addition, many of these molecules show a high degree of novelty from a structural and/or pharmacological point of view. Structure determination is generally achieved by the use of a variety of spectroscopic methods, among which NMR (nuclear magnetic resonance) plays a major role and determination of the stereochemical relationships within every new molecule is generally the most challenging part in structural determination. In this communication, we have chosen okadaic acid as a model compound to perform a computational chemistry study to predict ^1^H and ^13^C NMR chemical shifts. The effect of two different solvents and conformation on the ability of DFT (density functional theory) calculations to predict the correct stereoisomer has been studied.

## 1. Introduction

Marine natural products have generated great interest within the scientific community due to their fascinating biological activities, as well as by their extraordinary molecular diversity that have made them challenging problems for structure elucidation [1,2]. Structure determination is mostly achieved by interpretation of MS (mass spectrometry) and NMR (nuclear magnetic resonance) data and stereochemical assignments are generally the most time consuming step within this procedure [3]. This is particularly true when one has the need to assign independent molecular segments containing remote stereogenic centers, though it can be done by the use of the ^3^*J*_H,H_ and ^2,3^*J*_C,H_ providing an adequate number of them can be measured [4]. Asymmetric synthesis of the target molecule and subsequent comparison of their NMR spectroscopic data is another valuable and highly used approach, although very time consuming. Moreover, it has been proven that nowadays quantum mechanical calculations of NMR chemical shifts are also an excellent tool for determining molecular structures [5,6,7,8].

Two significant restrictions applies for the use of quantum-chemistry methods in structure determination, (1) computational limitations, related to the size of the studied system and the accuracy of the theoretical approach; and (2) bulk effects, mainly related to the interaction of the studied molecule with the solvent. With regard to the first issue, the continuous development of new computer facilities and computational methods allows, especially for atoms of the first two rows of the periodic table, the use of increasingly extended basis sets at either HF (Hartree-Fock), DFT (density functional theory), or post-HF methods, but still is a concern as the molecular size increases or when a large number of molecules have to be simulated. Nevertheless, it have been reported that the use of DFT methods, with relatively simple basis sets can yield accurate chemical shift predictions [9]. Concerning the second point, the effect of solvent on the computation of NMR parameters is relatively complex, as the studied molecules can be polarized by electrostatic interactions, make specific bonds, or simply change their conformation [10,11,12,13]. However, from a practical point of view, solvent effects on computed chemical shifts of small molecules dissolved in commonly used NMR solvents such as CDCl_3_ are frequently small [14]. As NMR chemical shifts are strongly affected by molecular conformation; geometry optimization is a crucial factor in an accurate computation of NMR chemical shifts. Regarding this point, Goodman *et al.* [15] have reported that, estimates of energy and isotropic shielding in solution by DFT methods can be reliably obtained by single-point calculations on the gas-phase of structures obtained from faster molecular mechanics conformational searches, thus, circumventing the need for time consuming optimizations in solvent. On the other hand, relatively few studies have considered the difference between results obtained with and without solvent models, but the general conclusion is that consideration of solvent generally leads to an improvement of the results [9,16].

**Figure 1:**
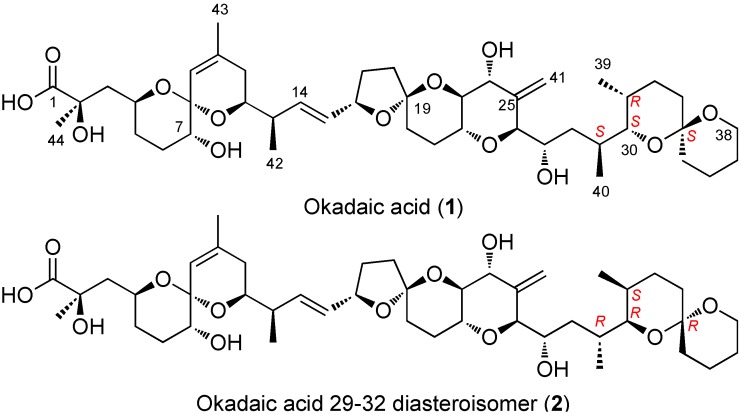
Structures of okadaic acid (**1**) and of the studied diasteroisomer (**2**).

In this work, we address the question of assigning one set of NMR experimental data to different possible structures where two stereoclusters are joined by an acyclic linker. We have chosen okadaic acid (**1** in Figure 1) as a structurally representative of a large group of marine toxins—that shares many common structural features—including yessotoxins, brevetoxins, ciguatoxins, palytoxins and other related compounds, such as amphidinolides, amphidinols, belizenolide, *etc.* [17,18,19]. Moreover, the structure of okadaic acid has been profusely studied by NMR and X-ray crystallography, and although it is a potentially flexible molecule (three acyclic portions can be identified within the molecule) it turns out to be conformationally restricted by the existence of an intramolecular H-bond between the carboxyl group at C-1 and the hydroxyl at C-24 [20,21,22,23]. Finally, okadaic acid is soluble in different solvents, so the influence of this parameter on the results of these calculations can also be addressed.

## 2. Results and Discussion

Our aim in this study is to verify that quantum mechanics computational simulations provide valid support to the structural characterization of the important group of polyether marine toxins, of which complex structures are usually elucidated on the basis of NMR spectral data. Thus, in this communication, the effect of two different solvents, the use of two different levels of theory and the influence of molecular conformation on the ability of DFT calculations to predict the correct stereoisomer has been studied. For this purpose, we present a systematic investigation of structure assignment using different statistical tools such as correlation coefficient (*R*^2^), corrected mean absolute deviation (CMAD) [5] and DP4 probability [6].

### 2.1. Crystallographic Structure of Okadaic Acid

The crystallographic structure of okadaic acid was used as our “gold” standard to reference all the calculations. Uemura *et al.* [24] published the structure after crystallization in methanol and the atomic coordinates were downloaded from the Cambridge Crystallographic Data Centre [25] under the accession number CCDC 691258.

### 2.2. Experimental NMR Data of Okadaic Acid

**Figure 2:**
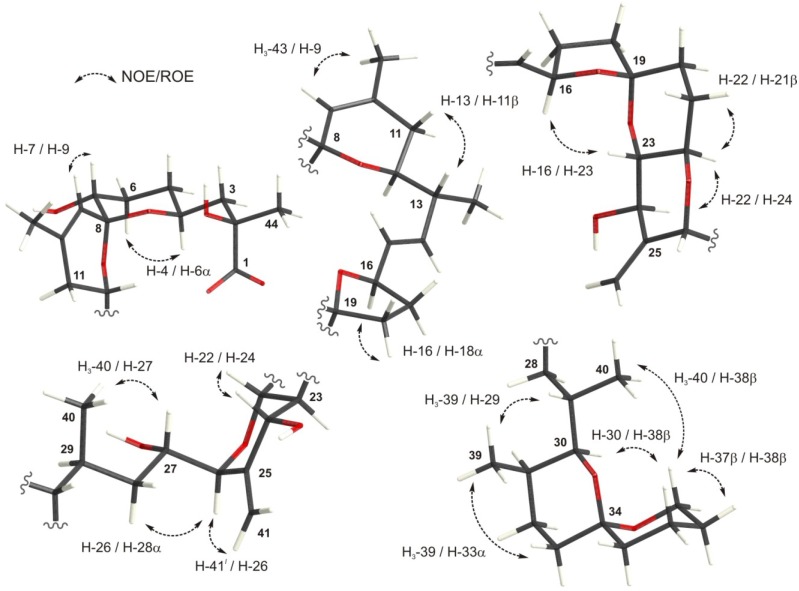
Relevant dipolar correlations observed for okadaic acid in CD_3_OD.

Accurate ^1^H and ^13^C NMR chemical shift assignments are critical for an appropriate comparison between experimental and calculated values. Stereospecific assignments for okadaic acid were available for almost all atoms when using CDCl_3_ as solvent (except for protons at C-20 and C-35) [23]. However, this was not the case when CD_3_OD is used as solvent, where nonstereospecific assignments were only available [21]. For this reason, we accomplished a full assignment of every proton in okadaic acid measuring ^3^*J*_HH_ values and dipolar correlations from a series of 1D-Selective TOCSY and NOESY as well as 2D ROESY experiments [26]. NMR data and relevant dipolar correlations are summarized in Figure 2 and Table 1.

**Table 1:** ^1^H and ^13^C NMR data for okadaic acid in CD_3_OD (*J* in Hz).

C	δ_C_	δ_H_	^3^*J*_H,H_	C	δ_C_	δ_H_	^3^*J*_H,H_
1	182.2	-	-	23	78.1	3.28	9.8, 9.8
2	76.0	-	-	24	71.8	3.92	9.8
3	46.1	1.79*^S^*	2.0, 12.0	25	146.7	-	-
		1.65*^R^*	11.0, 12.0				
4	68.2	3.92	2.0, 2.5, 9.0, 11.0	26	86.2	3.80	8.8
5	33.1	(β) 1.72*^R^*	4.0, 9.0, 10.0, 13.0	27	65.8	3.94	2.0, 8.8, 10.0
		(α) 1.30*^S^*	2.5, 2,5, 5.0, 13.0				
6	28.2	(α) 1.82*^R^*	4.0, 4.8, 5.0, 13.0	28	36.3	1.28*^R^*	2.6, 10.0, 12.0
		(β) 1.51*^S^*	2.5, 9.5, 10.0, 13.0			0.82*^S^*	2.0, 11.0, 12.0
7	73.2	3.22	4.8, 9.5	29	32.0	1.78	2.6, 6.4, 10.5, 11.0
8	97.1	-	-	30	76.7	3.13	2.2, 10.5
9	123.5	5.13	-	31	28.5	1.69	2.2, 2.5, 6.5, 6.9
10	138.8	-	-	32	27.5	(α) 1.88*^S^*	2.5, 2.5, 12.0, 12.0
						(β) 1.25*^R^*	2.0, 6.5, 6.5, 12.0
11	33.4	(β) 1.90*^R^*	11.0, 16.0	33	30.6	1.22(2H)	-
		(α) 1.69*^S^*	4.0, 16.0				
12	71.4	3.71	4.0, 8.0, 11.0	34	96.5		-
13	42.9	2.20	7.0, 8.0, 8.5	35	36.6	(β) 1.49*^R^*	2,5, 4.3, 13.0
						(α) 1.26*^S^*	2.5, 13.0, 13.0
14	137.2	5.81	8.5, 15.4	36	19.4	(α) 1.79*^S^*	2.5, 2.5, 4.3, 4.3, 13.0
						(β) 1.40*^R^*	2.5, 2.5, 13.0, 13.0, 13.0
15	131.9	5.34	7.9, 15.4	37	26.1	1.37(2H)	-
16	80.2	4.52	7.5, 7.5, 7.9	38	60.9	(β) 3.57*^S^*	3.0, 11.5, 12.0
						(α) 3.39*^R^*	2.5, 3.0, 12.0
17	31.0	(α) 2.04*^R^*	6.2, 7.5, 9.5, 12.0	39	10.6	0.79	6.9
		(β) 1.43*^S^*	5.0, 5.5, 7.5, 12.0				
18	37.7	(α) 1.88*^S^*	5.0,9.5, 12.5	40	16.8	0.91	6.4
		(β) 1.72*^R^*	5.5, 6.2, 12.5				
19	106.5	-	-	41	112.6	5.25	-
						4.91	
20	33.4	1.75 (2H)	-	42	16.9	0.98	7.0
21	27.5	(α) 1.75*^R^*	4.0, 13.0	43	22.9	1.59	-
		(β) 1.65*^S^*	10.0, 13.0				
22	71.1	3.50	4.0, 9.8, 10.0	44	27.5	1.16	-

### 2.3. Diasteroisomeric Structure of Okadaic Acid

**Figure 3:**
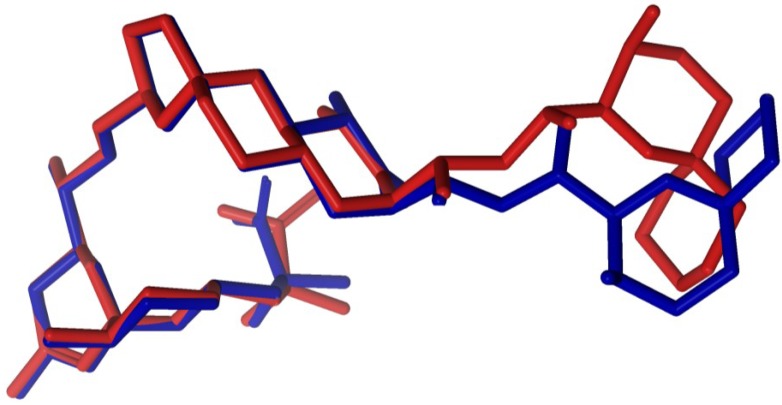
Crystallographic structure of okadaic acid in *blue* superimposed with the studied 29*R*, 30*R*, 31*S*, 34*R* diasteroisomer in *red*.

In order to test the possibility of differentiating an incorrect diasteroisomer of okadaic acid (**2**) from the correct structure (**1**), we assembled an alternative molecule where the whole C-29→C-38 stereocluster was inverted while the C-1→C-28 moiety maintained the same configuration (Figure 3). The crystallographic structure of okadaic acid was used as a template where the C-29→C-38 fragment was manually reoriented in order to find a similar extended conformation. Afterwards, an unrestrained minimization was performed in the previous structure to optimize the geometry. Therefore, the new molecule (**2**) shows the alternate configuration (29*R*, 30*R*, 31*S*, 34*R*) instead of the right one (29*S*, 30*S*, 31*R*, 34*S*). The selection of the C-29→C-38 moiety was based on the fact that it is spatially distant from the C-1→C-26 pseudo-macrocyclic portion of the molecule. In this way, we could simulate a situation where one has two well-defined stereoclusters but their relative configurations are difficult to connect. Indeed, it has been shown that correlating relative configurations of two separated “stereoarrangements” by NMR and DFT calculations is a fairly challenging task that has to be taken with caution [27]. Here, in principle, the introduced structural modifications would not induce large differences in the calculated chemical shifts, as changes in their chemical environments are minor. In addition, an acyclic linker connects the C-1→C-26 and C-29→C-38 stereoclusters and consequently, the effects of conformational flexibility could be tested.

### 2.4. Conformational Searches

The crystal structure of okadaic acid was used as a starting template for the conformational searches. Simulations were performed using the OPLS2005 force field [28], as implemented in MacroModel 9.9 [29] using the generalized Born/surface area (GBSA) solvent model. Searches were undertaken using the mixed torsional and low-mode sampling scheme [30], and all local minima found within 10 kJ of the global minimum were used in the DFT calculations.

### 2.5. Calculation of NMR Chemical Shifts

**Figure 4:**
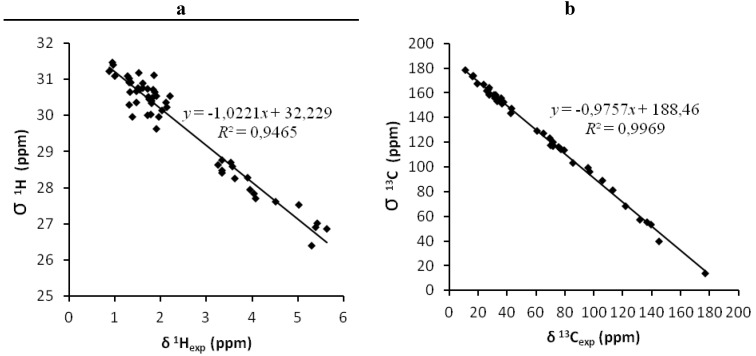
^1^H correlations (**a**) and ^13^C correlations (**b**) between calculated isotropic shieldings and experimental chemical shifts of okadaic acid. Fitting parameters are indicated for each nucleus.

Based on previously reported results, we decided to use two of the most popular hybrid functionals, the B3LYP [31,32,33,34] and the mPW1PW91 [35] with the 6 − 31 + G * basis set to calculate the isotropic chemical shielding values for all atoms within okadaic acid (**1**) and its diasteroisomer (**2**). In order to calculate the empirically scaled chemical shifts (δ_scaled_), we plot the experimental values measured either in CDCl_3_ or CD_3_OD against the theoretically calculated isotropic shieldings (Figure 4). Afterwards, the intercept (*a*) and the slope (*b*) of the regression line were used to calculated the scaled chemical shifts as δ_scaled_ = (δ_exp_ − *a*)/*b*. Using this approach, systematic errors can be compensated as the obtained values do not depend fundamentally on the calculation of one particular molecule, such as TMS (tetramethylsilane) [7].

We started all our calculations with the crystallographic structure of okadaic acid. Using its atomic coordinates, isotropic shieldings were calculated and subsequently scaled computed chemical shifts (δ_scaled_) were empirically obtained. This was done by applying linear regression parameters obtained from the plot of isotropic shieldings against the experimental chemical shifts (δ_exp_), as it can be seen in Figure 4. In this way, systematic errors, caused by an inaccurate reference value, can be avoided. This approach has been proposed as appropriate to remove systematic errors [7]. Alternatively, generic-scaling factors obtained from large datasets can be used, however, the results obtained were slightly worse. For instance, ^13^C and ^1^H CMAD values of 2.57 ppm and 0.27 ppm (chloroform) or 2.35 ppm and 0.23 ppm (methanol), respectively, were obtained using generic parameters taken from the CHESHIRE webpage [36] at the B3LYP/631G + (d,p) level in the gas phase as oppose to ^13^C and ^1^H CMADs of 1.90 ppm and 0.25 ppm in chloroform or 1.93 ppm and 0.22 ppm in methanol, respectively, when we used specific scaling factors obtained as described above.

The computed chemical shifts—either in the gas phase or using the Poisson Boltzmann finite (PBF) element method solvation model for chloroform or methanol—are shown in Table 2 and Table 3.

**Table 2:** Experimental and computed δ ^13^C for okadaic acid and the studied diasteroisomer.

C	1-Gas (CDCl_3_)	1-CHCl_3_ (CDCl_3_)	2-Gas (CDCl_3_)	2-CHCl_3_ (CDCl_3_)	Expt (CDCl_3_)	Expt (CD_3_OD)	1-Gas (CD_3_OD)	1-CH_3_OH (CD_3_OD)	2-Gas (CD_3_OD)	2-CH_3_OH (CD_3_OD)
1	178.7	179.5	172.9	172.5	177.1	182.2	180.2	180.7	174.3	176.0
2	76.2	76.5	73.9	74.8	77.0	76.0	76.8	77.1	74.5	75.8
3	42.1	42.1	43.2	43.0	42.8	46.1	42.4	42.2	43.4	43.0
4	66.8	67.6	68.3	68.0	69.8	68.2	67.3	68.1	68.8	67.8
5	31.1	31.3	30.9	30.7	32.0	33.1	31.3	31.4	31.1	30.9
6	31.2	31.3	35.3	35.7	27.5	28.2	31.4	31.5	35.5	35.9
7	73.1	72.4	71.4	71.0	72.0	73.2	73.7	72.8	71.9	71.2
8	94.8	94.4	94.5	93.6	97.0	97.1	95.6	95.1	95.2	93.0
9	123.5	120.2	123.3	120.2	121.9	123.5	124.5	119.7	124.3	118.7
10	138.3	140.9	138.4	141.7	139.9	138.8	139.4	143.9	139.5	144.0
11	34.5	34.6	31.6	31.3	33.6	33.4	34.7	35.0	31.8	31.6
12	70.1	69.7	67.5	68.3	72.0	71.4	70.7	70.5	68.0	67.5
13	46.3	45.9	46.2	45.7	42.6	42.9	46.6	46.2	46.5	46.1
14	136.3	136.8	134.0	135.0	136.9	137.2	137.4	139.1	135.1	135.3
15	134.2	133.4	139.6	138.5	131.8	131.9	135.3	133.6	140.8	138.2
16	76.6	76.5	74.9	75.2	79.6	80.2	77.2	76.8	75.5	75.3
17	30.6	30.6	29.0	28.9	31.1	31.0	30.8	30.6	29.2	29.0
18	36.7	36.9	33.1	33.3	37.7	37.7	36.9	37.3	33.3	33.5
19	101.9	101.8	104.1	103.4	106.1	106.5	102.7	102.6	104.9	104.2
20	35.9	35.6	35.7	35.6	33.2	33.4	36.1	35.8	36.0	35.8
21	26.3	26.2	26.5	26.6	27.0	27.5	26.5	26.3	26.6	26.9
22	72.4	72.5	72.9	70.1	70.2	71.1	72.9	72.8	73.5	70.3
23	75.5	75.3	73.1	72.5	76.9	78.1	76.1	75.9	73.7	73.4
24	72.7	72.4	72.9	72.7	71.6	71.8	73.3	73.0	73.4	73.8
25	152.3	151.4	148.8	149.8	145.2	146.7	153.5	151.9	150.0	152.6
26	87.6	87.0	90.9	89.6	85.3	86.2	88.3	87.5	91.6	90.9
27	63.0	63.1	72.3	71.1	65.0	65.8	63.5	63.5	72.8	71.8
28	33.9	34.1	38.5	39.9	35.7	36.3	34.1	34.4	38.7	40.9
29	33.7	33.8	41.9	41.6	31.5	32.0	33.9	34.3	42.2	41.9
30	74.1	74.4	72.5	74.6	75.5	76.7	74.7	74.9	73.1	75.3
31	29.9	29.9	34.3	34.6	27.8	28.5	30.1	30.0	34.5	34.9
32	26.6	26.8	23.5	24.9	26.8	27.5	26.8	27.0	23.6	25.1
33	31.8	32.0	29.4	29.4	30.8	30.6	32.0	32.2	29.5	29.8
34	91.6	92.2	93.6	93.9	96.0	96.5	92.3	93.0	94.3	94.8
35	38.2	38.6	37.5	37.7	36.3	36.6	38.4	39.0	37.7	38.2
36	21.7	21.8	21.1	22.0	19.2	19.4	21.8	22.0	21.2	22.2
37	27.3	27.9	25.3	26.4	25.9	26.1	27.5	28.1	25.5	26.7
38	60.7	60.7	58.2	58.5	60.8	60.9	61.1	61.3	58.6	59.3
39	10.6	10.5	12.0	12.3	11.1	10.6	10.6	10.5	12.0	12.4
40	15.1	14.8	13.0	12.4	16.6	16.6	15.2	14.7	13.0	12.7
41	109.8	110.4	117.5	118.3	113.0	112.6	110.7	111.8	118.5	118.8
42	15.9	15.6	12.4	11.9	16.3	16.9	16.0	15.6	12.4	12.2
43	22.3	22.1	20.3	20.0	23.5	22.9	22.4	22.1	20.4	20.1
44	24.6	25.1	20.3	19.8	27.7	27.5	24.7	25.3	20.4	19.2

1-gas (CDCl_3_): δ_scaled_ computed for **1** in the gas phase and scaled against experimental data obtained in CDCl_3_ solution. 1-CHCl_3_ (CDCl_3_): δ_scaled_ computed for **1** using a CHCl_3_ solvation model and scaled against experimental data obtained in CDCl_3_ solution. 2-gas (CDCl_3_): δ_scaled_ computed for **2** in the gas phase and scaled against experimental data obtained in CDCl_3_ solution. 2-CHCl_3_ (CDCl_3_): δ_scaled_ computed for **2** using a CHCl_3_ solvation model and scaled against experimental data obtained in CDCl_3_ solution. Expt (CDCl_3_): Experimental NMR data of **1** obtained in CDCl_3_ solution. Expt (CD_3_OD): Experimental NMR data of **1** obtained in CD_3_OD solution. 1-gas (CD_3_OD): δ_scaled_ computed for **1** in the gas phase and scaled against experimental data obtained in CD_3_OD solution. 1-CH_3_OH (CD_3_OD): δ_scaled_ computed for **1** using a CH_3_OH solvation model and scaled against experimental data obtained in CD_3_OD solution. 2-gas (CD_3_OD): δ_scaled_ computed for **2** in the gas phase and scaled against experimental data obtained in CD_3_OD solution. 2-CH_3_OH (CD_3_OD): δ_scaled_ computed for **2** using a CH_3_OH solvation model and scaled against experimental data obtained in CD_3_OD solution.

**Table 3:** Comparison of experimental and computed δ ^1^H for okadaic acid.

C	1-Gas (CDCl_3_)	1-CHCl_3_ (CDCl_3_)	2-Gas (CDCl_3_)	2-CHCl_3_ (CDCl_3_)	Expt (CDCl_3_)	Expt (CD_3_OD)	1-Gas (CD_3_OD)	1-CH_3_OH (CD_3_OD)	2-Gas (CD_3_OD)	2-CH_3_OH (CD_3_OD)
H3 *	1.43	1.54	1.59	1.56	1.62	1.65	1.35	1.49	1.52	1.35
H3 ^†^	1.83	1.83	1.46	1.61	2.12	1.79	1.75	1.75	1.39	1.86
H4	4.20	4.24	3.71	3.95	3.96	3.92	4.15	4.16	3.63	3.62
H5 ^†^	1.45	1.36	1.21	1.35	1.31	1.30	1.37	1.25	1.14	1.14
H5 *	1.17	1.36	1.96	1.94	1.72	1.72	1.08	1.34	1.89	1.84
H6 ^†^	1.69	1.66	1.86	1.59	1.83	1.51	1.62	1.54	1.80	1.41
H6 *	2.16	2.08	1.87	2.06	1.79	1.82	2.09	1.93	1.80	1.71
H7	3.38	3.65	3.10	3.21	3.34	3.22	3.32	3.65	3.03	3.00
H9	5.71	5.60	5.50	5.51	5.29	5.13	5.68	5.41	5.42	5.22
H11 *	1.54	1.61	1.71	1.80	1.87	1.90	1.46	1.65	1.64	1.89
H11 ^†^	1.66	1.80	1.45	1.76	1.91	1.69	1.58	1.80	1.38	1.45
H12	3.72	3.37	3.97	4.03	3.35	3.71	3.67	3.29	3.89	4.09
H13	1.65	1.85	1.51	2.16	2.21	2.20	1.57	1.88	1.44	1.65
H14	5.26	5.23	5.37	5.29	5.63	5.81	5.23	5.24	5.29	5.13
H15	5.10	5.17	5.60	5.70	5.42	5.34	5.06	5.15	5.52	5.22
H16	4.52	4.48	4.14	4.03	4.51	4.52	4.47	4.31	4.06	3.90
H17	1.48	1.61	1.91	1.92	1.54	1.43	1.40	1.51	1.84	1.73
H17 *	1.96	2.01	1.30	1.48	2.14	2.04	1.89	1.89	1.23	1.42
H18 ^†^	2.05	1.95	1.96	1.99	2.04	1.88	1.98	1.78	1.89	1.82
H18 *	1.85	1.95	1.76	1.90	1.80	1.72	1.77	1.91	1.69	1.95
H20 *	1.84	1.87	1.75	1.75	1.47	1.75	1.76	1.83	1.68	1.70
H20 ^†^	1.90	1.96	1.74	1.70	1.32	1.75	1.83	1.84	1.67	1.60
H21 ^†^	1.78	1.82	1.78	1.55	1.81	1.65	1.70	1.78	1.71	1.35
H21 *	2.17	2.05	2.28	1.99	1.72	1.75	2.10	1.89	2.21	1.82
H22	3.56	3.55	4.58	4.17	3.57	3.50	3.51	3.57	4.50	4.15
H23	3.68	3.70	3.77	3.62	3.35	3.28	3.62	3.60	3.70	3.62
H24	4.43	4.36	4.07	4.00	4.07	3.92	4.38	4.36	4.00	4.15
H26	3.87	3.93	4.16	4.34	3.90	3.80	3.82	3.97	4.08	4.71
H27	4.30	4.35	5.77	5.48	4.04	3.94	4.25	4.32	5.69	5.61
H28 *	1.11	1.08	1.83	1.63	1.28	1.28	1.03	1.07	1.76	1.64
H28 ^†^	0.75	0.60	0.84	1.08	0.95	0.82	0.66	0.53	0.78	1.19
H29	2.55	2.24	1.59	1.61	1.91	1.78	2.49	2.06	1.52	1.65
H30	3.52	3.49	3.22	3.45	3.25	3.13	3.46	3.42	3.15	3.38
H31	1.75	1.72	1.40	1.57	1.75	1.69	1.67	1.68	1.34	1.49
H32 *	1.10	1.18	1.68	1.64	1.86	1.25	1.01	1.12	1.61	1.30
H32 ^†^	2.22	2.05	0.84	1.05	1.96	1.88	2.15	1.88	0.77	0.84
H33 *	1.54	1.63	0.51	0.90	1.34	1.22	1.46	1.56	0.45	0.67
H33 ^†^	1.03	1.13	1.50	1.58	1.52	1.22	0.94	1.02	1.43	1.53
H35 ^†^	1.53	1.50	2.04	1.74	1.31	1.26	1.45	1.39	1.97	1.68
H35 *	1.28	1.44	0.69	1.07	1.48	1.49	1.20	1.38	0.63	0.95
H36 ^†^	1.30	1.32	1.56	1.42	1.61	1.79	1.21	1.26	1.49	1.35
H36 *	2.21	2.03	2.62	2.29	1.39	1.40	2.14	1.89	2.55	2.24
H37 ^†^	1.44	1.33	1.17	1.49	1.51	1.37	1.36	1.27	1.11	1.30
H37 *	1.47	1.58	1.50	1.35	1.84	1.37	1.39	1.48	1.44	1.26
H38 ^†^	3.88	3.75	3.72	3.53	3.62	3.57	3.83	3.67	3.65	3.49
H38 *	3.44	3.54	2.65	3.14	3.53	3.39	3.39	3.49	2.58	3.10
H39	0.99	0.92	1.22	1.20	0.88	0.79	0.90	0.81	1.16	1.12
H40	1.11	0.95	2.08	1.61	1.01	0.91	1.02	0.88	2.01	1.55
H41	4.60	4.81	4.34	4.44	5.02	4.91	4.56	4.87	4.26	4.67
H41	5.22	5.11	5.23	5.19	5.39	5.25	5.18	5.07	5.16	5.08
H42	0.80	0.82	0.90	0.73	0.97	0.98	0.71	0.76	0.83	0.91
H43	1.68	1.70	1.65	1.77	1.73	1.59	1.61	1.64	1.58	1.64
H44	1.28	1.28	1.51	1.40	1.36	1.16	1.20	1.18	1.44	1.29

Stereoheterotopic hydrogens are identified as *pro-R* (*) or *pro-S* (^†^). Headings are equal to those in Table 2.

There is an overall good and similar agreement between experimental and computed values as can be deduced from the corresponding average errors. Thus, the corrected mean absolute deviations:

CMAD = (1/*n*) Σ |δ_scaled_ − δ_exp_| (1)

obtained for ^1^H and ^13^C were 0.25 ppm and 1.89 ppm or 0.24 ppm and 1.84 ppm, respectively when the gas phase calculations were compared with the experimental values measured in CDCl_3_ or CD_3_OD respectively (Table 4). Very similar results were obtained using the mPW1PW91/6 − 31 + G * level of theory. It is also apparent from the results that calculations done using solvation models produced better results than those performed in the gas phase, particularly for ^1^H chemical shifts. Smaller CMADs were obtained when the experimental data was compared against the computed values using the corresponding solvation model: 0.21 ppm for ^1^H and 1.80 ppm for ^13^C were found when using chloroform and similar values of 0.21 ppm for ^1^H and 1.94 ppm for ^13^C using methanol. The correlation coefficients *R*^2^ are also fairly informative, thus when the gas phase calculated values are correlated with the experimental data measured in solution, the quality of the correlation is slightly lower than those obtained when the computed values including a solvation model are used (Table 4).

The same procedure was followed for diasteroisomer **2**, obtaining parallel trends with those observed for **1**, *i.e.*, only minor improvements taking into account solvent effects and comparable errors at the two different levels of theory used (Supplementary Information). However, larger CMAD values and smaller correlation coefficients *R*^2^ were obtained in all circumstances as can be seen in Table 4.

**Table 4:** Summary of the statistical analyses performed.

Structure	^13^C B3LYP			^1^H B3LYP			^13^C mPW1PW91			^1^H mPW1PW91		
	CMAD	*R*^2^	DP4	CMAD	*R*^2^	DP4	CMAD	*R*^2^	DP4	CMAD	*R*^2^	DP4
*CHCl_3_ as Solvent*												
**1_gas_ (A)**	1.89	0.9969	-	0.25	0.9465	-	1.81	0.9969	-	0.24	0.9499	-
**2_gas_ (B)**	3.29	0.9907	-	0.38	0.8677	-	3.18	0.9917	-	0.35	0.8904	-
**A *vs.* B**	-	-	100	-	-	100	-	-	100	-	-	100
**1_CHCl3_ (C)**	1.80	0.9972	-	0.22	0.9598	-	1.67	0.9973	-	0.21	0.9623	-
**2_CHCl3_ (D)**	3.21	0.9909	-	0.28	0.9222	-	3.10	0.9916	-	0.26	0.9346	-
**C *vs.* D**	-	-	100	-	-	100	-	-	100	-	-	100
**1_CH3OH_ (E)**	1.89	0.9972	-	0.22	0.9539	-	1.78	0.9971	-	0.23	0.9565	-
**2_CH3OH_ (F)**	3.31	0.9903	-	0.34	0.9170	-	3.09	0.9908	-	0.27	0.9300	-
**E *vs.* F**	-	-	100	-	-	100	-	-	100	-	-	100
**CS I (G)**	2.08	0.9958		0.31	0.9268		1.95	0.9961	-	0.27	0.9365	-
**G *vs.* B**	-	-	100	-	-	100	-	-	100	-	-	100
**CS II (H)**	2.35	0.9914	-	0.32	0.9229	-	2.22	0.9923	-	0.31	0.9365	-
**H *vs.* B**	-	-	100	-	-	100	-	-	100	-	-	100
*CH_3_OH as solvent*												
**1_gas_ (I)**	1.84	0.9970	-	0.24	0.9415	-	1.78	0.997	-	0.24	0.9445	-
**2_gas_ (J)**	3.32	0.9904	-	0.34	0.8893	-	3.22	0.9914	-	0.30	0.9099	-
**I *vs.* J**	-	-	100	-	-	100	-	-	100	-	-	100
**1_CH3OH_ (K)**	1.94	0.9969	-	0.21	0.9596		1.88	0.997	-	0.21	0.9589	-
**2_CH3OH_ (L)**	3.51	0.9899	-	0.29	0.9163	-	3.28	0.9909	-	0.21	0.9513	-
**K *vs.* L**	-	-	100	-	-	100	-	-	100	-	-	100
**1_CHCl3_ (M)**	1.93	0.9971	-	0.23	0.9639	-	1.81	0.9973	-	0.22	0.9626	-
**2_CHCl3_ (N)**	3.43	0.9903	-	0.25	0.9039	-	3.32	0.9917	-	0.23	0.9389	-
**M *vs.* N**	-	-	100	-	-	100	-	-	100	-	-	100
**CS I (O)**	1.75	0.9971	-	0.30	0.9299	-	1.68	0.9973	-	0.27	0.9396	-
**O *vs.* J**	-	-	100	-	-	100	-	-	100	-	-	100
**CS II (P)**	2.58	0.9913	-	0.30	0.9201	-	2.46	0.9921	-	0.27	0.9329	-
**P *vs.* J**	-	-	100	-	-	100	-	-	100	-	-	100

1_gas_: δ_scaled_ computed for **1** in the gas phase and scaled *vs.* experimental data obtained in CDCl_3_ solution; 2_gas_: δ_scaled_ computed for **2** in the gas phase and scaled *vs.* experimental data obtained in CDCl_3_ solution; 1_CHCl3_: δ_scaled_ computed for **1** using a CHCl_3_ solvation model and scaled *vs.* experimental data obtained in CDCl_3_ solution; 2_CHCl3_: δ_scaled_ computed for **2** using a CHCl_3_ solvation model and scaled *vs.* experimental data obtained in CDCl_3_ solution; 1_CH3OH_: δ_scaled_ computed for **1** using a CH_3_OH solvation model and scaled *vs.* experimental data obtained in CDCl_3_ solution; 2_CH3OH_: δ_scaled_ computed for **2** using a CH_3_OH solvation model and scaled *vs.* experimental data obtained in CDCl_3_ solution; CS I: δ_scaled_ computed for conformers obtained in conformational search I (CSI) in the gas phase and scaled *vs.* experimental data obtained in CDCl_3_ solution; CS II: δ_scaled_ computed for conformers obtained in conformational search II (CSI) in the gas phase and scaled *vs.* experimental data obtained in CDCl_3_ solution; 1_gas_: δ_scaled_ computed for **1** in the gas phase and scaled *vs.* experimental data obtained in CD_3_OD solution; 2_gas_: δ_scaled_ computed for **2** in the gas phase and scaled *vs.* experimental data obtained in CD_3_OD solution; 1_CH3OH_: δ_scaled_ computed for **1** using a CH_3_OH solvation model and scaled *vs.* experimental data obtained in CD_3_OD solution; 2_CH3OH_: δ_scaled_ computed for **2** using a CH_3_OH solvation model and scaled *vs.* experimental data obtained in CD_3_OD solution; 1_CHCl3_: δ_scaled_ computed for **1** using a CHCl_3_ solvation model and scaled *vs.* experimental data obtained in CD_3_OD solution; 2_CHCl3_: δ_scaled_ computed for **2** using a CHCl_3_ solvation model and scaled *vs.* experimental data obtained in CD_3_OD solution; CS I: δ_scaled_ computed for conformers obtained in conformational search I (CSI) in the gas phase and scaled *vs.* experimental data obtained in CDCl_3_ solution; CS II: δ_scaled_ computed for conformers obtained in conformational search II (CSI) in the gas phase and scaled *vs.* experimental data obtained in CDCl_3_ solution.

From the previous statistical analysis a selection of the correct stereoisomer looks possible. Still, a critical part in any structure prediction using chemical shift calculations relies in the quantification of the fit obtained for each possible calculated structure. For this purpose, Smith and Goodman have shown that using the DP4 probability analysis it is feasible to assign stereochemical relationships with quantifiable confidence [5]. This approach takes the error probabilities for each computed chemical shift and, subsequently, using the Bayes theorem, it transforms their product into an overall probability that the structure is right. Indeed, when we compared the scaled ^1^H and ^13^C chemical shifts (δ_scaled_) of **1** and **2** calculated in the gas phase with the experimentally observed values either in chloroform or in methanol it turned out that the DP4 analysis always identified the correct structure (**1**) as the most likely one, with a 100% probability (Table 4). Therefore, it seems that despite the use of solvation models in the calculations improves the quality of the computed values, (Table 4) the DP4 analysis is equally able to select the right stereoisomer with great confidence taking into account the solvents or not. This result is in agreement with previously reported observations [15,37], and it is probably due to the fact that chemical shift differences are calculated more accurately than the shifts themselves because of the cancellation of systematic errors. Moreover, comprehensive studies considering the solvent influence in this type of calculations have concluded its impact on chemical shifts is mainly of indirect nature as the nature of solvent affects conformational populations and subsequently the shielding constants [12,13]. Actually, although both structures (**1** and **2**) improved their data quality when solvation models were considered, no overall advantage in the capability to discriminate the correct structure is gained when using the DP4 analysis [38].

**Figure 5:**
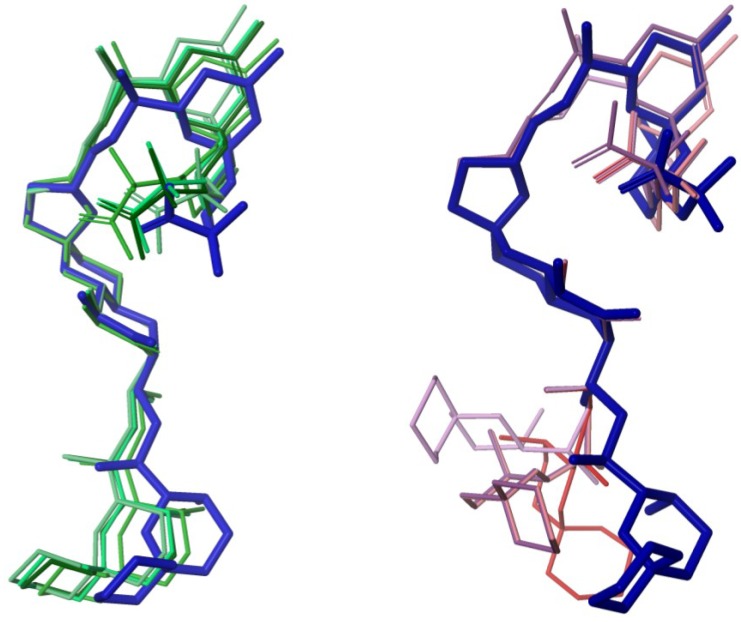
Crystallographic structure of okadaic acid (*blue*) superimposed with the energetically representative structures obtained from two conformational searches. Structures of an NMR compatible search (CS I) are in greenish and those obtained from an incompatible search (CS II) in reddish.

Up to this point, all our calculations have been done using a single, static structure, that is, using the crystallographic coordinates of okadaic acid and comparing them with those generated for its diasteroisomer **2**. Therefore, we have not taken into consideration that okadaic acid has several conformational degrees of freedom in its three acyclic moieties at C-1→C-4, C-12→C-16, and C26→C30. How good would the results be using a group of structures obtained from a molecular mechanics conformational search? What would happen if the conformational search was not good enough to find the global minimum? Although, an analysis of X-ray data and NMR coupling constants indicate that okadaic acid seems to be conformationally restricted by the existence of an intramolecular H-bond between the carboxyl group at C-1 and the hydroxyl at C-24 (Table 1) [19,20,21], we also wanted to check the importance of conformation in a chemical shift based analysis. Thus, we generated two ensembles of structures for **1**; the first one (CS I) was the result of a large-enough conformational search where the selected structures are in agreement with the NMR data (^3^*J*_H,H_ and dipolar correlations) and the second group (CS II) resulted from a short conformational search where the C-25→C-28 dihedral angle resulted incompatible with the NMR data (Figure 5).

When ^1^H and ^13^C chemical shifts, computed using the structures obtained from both conformational searches (CS I and CS II), were compared with the data calculated for **2**, it turned out that in all circumstances, the DP4 analysis selected the correct stereoisomer with 100% probabilities (Table 4). Nevertheless, as expected, the quality of the results obtained from CS I is better than those obtained from CS II. Thus, the CMADs obtained using the structures of CS I are smaller than those of CS II, in particular for ^13^C chemical shifts. Likewise, the corresponding correlation coefficients *R*^2^ follow the same trend, these are better results for CS I than for CS II (Table 4).

## 3. Experimental Section

### 3.1. Instrumentation and General Methods

NMR spectra were recorded on a Bruker AVANCE III 600 instrument equipped with a 5 mm TCI cryoprobe (Bruker, Rheinstetten, Germany). NMR spectra were obtained dissolving okadaic acid in CD_3_OD (99.8 + atom% D, Eurisotope) and CDCl_3_ (99.5 atom% D, Eurisotop). Chemical shifts are reported relative to solvent: CDCl_3_ (δ_H_ 7.26 and δ_C_ 77.0 ppm); CD_3_OD (δ_H_ 3.16, 4.75 and δ_C_ 48.3 ppm) at 300 K and coupling constants were calculated in Hz. NMR assignments were obtained from examination of 1D and 2D experiments (^1^H, ^13^C, DQF-COSY and HSQC). Spectral widths of 4200 and 22,500 Hz, and acquisition times of 0.57 and 0.24 s, were used in ^1^H-^1^H and ^1^H-^13^C experiments, respectively. Prior to Fourier transformation, zero filling was performed to expand the data to at least double the number of acquired data points. Sine bell shifted or exponential window functions with line broadening coefficients ranging from 0.1 to 3 Hz were used for 2D and 1D experiments respectively. HPLC analyses were performed on a Waters instrument equipped with a differential diffractometer detector and an X-Terra column. TLCs were carried out using Si gel Merck 60G, and were visualized with 10% phosphomolybdic acid in ethanol.

### 3.2. Prorocentrum Belizeanum Cultures

The strain of the dinoflagellate *P. belizeanum* used in this work (PBMA01), originally isolated from a coral reef of La Reunion Island, Indian Ocean, France, was obtained from the culture collection of phytoplankton cultures at the Centro Oceanográfico at Vigo, courtesy of Santiago Fraga (country). Cultures of *P. belizeanum* were grown in 250 mL flasks containing 150 mL of sea water enriched with Guillard K medium at 23 °C, at a salinity of 35, with an irradiance of 60 μE s^−1^ m^−2^ and under a 18:6 light:darkness photo cycle. Cultures were incubated statically for 6 weeks up to a final volume of 1.5 L.

### 3.3. Extraction and Isolation of Okadaic Acid

The cells from labelled cultures were filtered and extracted with methanol. The extract was chromatographed on Sephadex LH 20 using methanol as eluent. The fractions that containing the enriched toxin were chromatographed on reverse phase C-18 and the final purification was carried out in a HPLC Water instrument using a XTerra column eluted with methanol:water 4:1.

### 3.4. Computational Methods

Conformational searches were performed using the Macromodel software (version 8.5, Schrödinger Inc., San Diego, CA, USA) and the OPLS2005 force field [28,29]. Solvation effects were simulated using the generalized Born/surface area (GBSA) solvation model with chloroform or methanol. Extended nonbonded cutoff distances (a van der Waals cutoff of 8.0 Å and an electrostatic cutoff of 20.0 Å) were used. Local minima within 10 kJ of the global minimum were saved. Analysis of the results was undertaken using Maestro software.

Quantum mechanical calculations were carried out with Jaguar package (Jaguar; Schrödinger LLC, New York, NY, USA). Single point energy calculations were performed at the DFT theoretical level either in gas phase of using a Poisson-Boltzmann finite element method solvation model. B3LYP [31,32,33,34] and the mPW1PW91 [35] hybrid functionals with the 6 − 31G + (d) basis set were used. Chemical shifts were calculated using the gauge-including atomic orbital (GIAO) method [39]. Chemical shifts were calculated from their shielding constants that were first averaged according to their relative Boltzmann populations. Proton chemical shifts for each methyl group were averaged due to their conformational freedom.

## 4. Conclusions

We have tested the importance of various factors that can influence the results obtained in the calculation of ^1^H and ^13^C chemical shifts using an archetype of polyether marine toxin, okadaic acid. Quantum mechanical calculations using density functional theory can predict chemical shifts to a good-enough degree of accuracy to resolve many structural determination problems in this type of molecules. This includes challenging situations that arise when one has to decide between different stereoisomers containing remote stereogenic centers, where nuclei closest to the sites of major structural difference do not always show the largest differences in calculated shift.

A first conclusion is that the use of very large basis sets in these calculations it is not absolutely necessary [9]. In this study we have been able to select the correct diasteroisomer in a complex situation using the relatively modest 6 − 31 + G *. The inclusion of solvent effects in the calculations generally improve the quality of the results, but as all calculated structures do it, no overall advantage in the capability to discriminate the correct structure is gained. This it is probably due to the fact that chemical shift differences are calculated more accurately than the shifts themselves because of the cancellation of systematic errors [37,38]. With regard to the effect of conformational variability on the results of this kind of analysis, very similar CMADs were obtained using either the X-ray derived structure or an ensemble of structures obtained from an appropriate conformational search (the structures were in agreement with NMR derived dihedral angles and distances). However, when an ensemble of structures including a C25–C28 dihedral angle incompatible with NMR data was used, the quality of the fitting diminished but was still better than those obtained using the inappropriate diasteroisomer (**2**). Our results suggest that although the relationship between structural modifications and chemical shift differences is complex analyses based on quantum mechanical calculations of NMR chemical shifts is robust enough to help with structure elucidation of complex natural products.

## Supplementary Files

Supplementary File 1 Supplementary Information (PDF, 2450 KB)
