# Supplementary material for: Stereochemistry of Complex Marine Natural Products by Quantum Mechanical Calculations of NMR Chemical Shifts: Solvent and Conformational Effects on Okadaic Acid
Source: Mar Drugs. 2014 Jan 7;12(1):176–92. doi: 10.3390/md12010176 (PMC3917268; doi:10.3390/md12010176)
Supplement: Supplementary File 1 — Supplementary Information (PDF, 2450 KB) [file marinedrugs-12-00176-s001.pdf]

Supplementary Information

**Table S1.**	^{1}H and ^{13}C NMR data for okadaic acid in CD_3OD	2
**Table S2.**	^{1}H and ^{13}C NMR data for okadaic acid in $CDCl_3$	3
**Figure S1.**	^{1}H NMR spectrum of okadaic acid in CD_3OD	4
**Figure S2.**	^{13}C NMR spectrum of okadaic acid in CD_3OD	4
**Figure S3.**	ROESY spectrum of okadaic acid in CD_3OD	5
**Figure S4.**	HSQC spectrum of okadaic acid in CD_3OD	6
**Figure S5.**	1D-TOCSY spectrum of okadaic acid in CD_3OD (H-14, H-15, H-16)	7
**Figure S6.**	1D-TOCSY spectrum of okadaic acid in CD_3OD (H-27, H-24, H-26, H-12)	8
**Figure S7.**	1D-TOCSY spectrum of okadaic acid in CD_3OD (H-38α, H-22, H-38β)	9
**Figure S8.**	1D-TOCSY spectrum of okadaic acid in CD_3OD (H-30, H-13, H-17α)	10
**Table S3.**	Computed ^{13}C and ^{1}H chemical shifts for okadaic acid (**1**) and the studied diasteroisomer (**2**) on B3LYP-6-31G + (d)	11
**Table S4.**	Computed ^{13}C and 1H chemical shifts for okadaic acid (**1**) and the studied diasteroisomer (**2**) on mPW1Pw91-6-31G + (d)	13

S1. Correlation Graphs. B3LYP-6-31G + (d) 16
S2. Correlation Graphs. mPW1Pw91-6-31G + (d) 21
S3. Energies and Coordinates for studied okadaic acid 29–32 diasteroisomer (2) 26
S4. Representative NMR Compatible Conformational Search (CS I) 29
S5. Representative NMR Incompatible Conformational Search (CS II) 45

Table S1. ^{1}H and ^{13}C NMR data for okadaic acid in CD_3OD (J in Hz).

**C**	δ_C	δ_H	$^3J_{H,H}$	**C**	δ_C	δ_H	$^3J_{H,H}$
**1**	182.2	-	-	**23**	78.1	3.28	9.8, 9.8
**2**	76.0	-	-	**24**	71.8	3.92	9.8
**3**	46.1	1.79^S<br>1.65^R	2.0, 12.0<br>11.0, 12.0	**25**	146.7	-	-
**4**	68.2	3.92	2.0, 2.5, 9.0, 11.0	**26**	86.2	3.80	8.8
**5**	33.1	1.72^R<br>1.30^S	4.0, 9.0, 10.0, 13.0<br>2.5, 2,5, 5.0, 13.0	**27**	65.8	3.94	2.0, 8.8, 10.0
**6**	28.2	1.82^R<br>1.51^S	4.0, 4.8, 5.0, 13.0<br>2.5, 9.5, 10.0, 13.0	**28**	36.3	1.28^R<br>0.82^S	2.6, 10.0, 12.0<br>2.0, 11.0, 12.0
**7**	73.2	3.22	4.8,9.5	**29**	32.0	1.78	2.6, 6.4, 10.5, 11.0
**8**	97.1	-	-	**30**	76.7	3.13	2.2, 10.5
**9**	123.5	5.13	-	**31**	28.5	1.69	2.2, 2.5, 6.5, 6.9
**10**	138.8	-	-	**32**	27.5	1.88^S<br>1.25^R	2.5, 2.5, 12.0, 12.0<br>2.0, 6.5, 6.5, 12.0
**11**	33.4	1.90^R<br>1.69^S	11.0, 16.0<br>4.0, 16.0	**33**	30.6	1.22(2H)	-
**12**	71.4	3.71	4.0, 8.0, 11.0	**34**	96.5	-	-
**13**	42.9	2.20	7.0, 8.0, 8.5	**35**	36.6	1.49^R<br>1.26^S	2,5, 4.3, 13.0<br>2.5, 13.0, 13.0
**14**	137.2	5.81	8.5, 15.4	**36**	19.4	1.79^S<br>1.40^R	2.5,2.5,4.3,4.3,13.0<br>2.5, 2.5, 13.0, 13.0, 13.0
**15**	131.9	5.34	7.9, 15.4	**37**	26.1	1.37(2H)	-
**16**	80.2	4.52	7.5, 7.5, 7.9	**38**	60.9	3.57^S<br>3.39^R	3.0, 11.5, 12.0<br>2.5, 3.0, 12.0
**17**	31.0	2.04^R<br>1.43^S	6.2, 7.5, 9.5, 12.0<br>5.0, 5.5, 7.5, 12,0	**39**	10.6	0.79	6.9
**18**	37.7	1.88^S<br>1.72^R	5.0, 9.5, 12.5<br>5.5, 6.2, 12.5	**40**	16.8	0.91	6.4
**19**	106.5	-	-	**41**	112.6	5.25<br>4.91	-
**20**	33.4	1.75(2H)	-	**42**	16.9	0.98	7.0
**21**	27.5	1.75^R<br>1.65^S	4.0, 13.0<br>10.0, 13.0	**43**	22.9	1.59	-
**22**	71.1	3.50	4.0, 9.8, 10.0	**44**	27.5	1.16	-

Table S2. ^{1}H and ^{13}C NMR data for okadaic acid in $CDCl_3$.

C	δ_C	δ_H	C	δ_C	δ_H
**1**	176.7	-	**23**	76.5	3.35
**2**	77.2	-	**24**	71.1	4.07
**3**	42.6	2.12^{S} 1.62^{R}	**25**	144.7	-
**4**	69.3	3.96	**26**	84.9	3.90
**5**	31.6	1.72^{S} 1.31^{R}	**27**	64.7	4.04
**6**	32.8	1.83^{S} 1.79^{R}	**28**	35.3	1.28^{R} 0.95^{S}
**7**	71.5	3.34	**29**	31.1	1.91
**8**	96.5	-	**30**	75.0	3.25
**9**	121.5	5.29	**31**	27.4	1.75
**10**	139.4	-	**32**	26.6	1.96^{S} 1.86^{R}
**11**	33.2	1.91^{S} 1.87^{R}	**33**	30.3	1.52^{R} 1.34^{S}
**12**	71.5	3.35	**34**	95.6	
**13**	42.2	2.21	**35**	25.4	1.48 1.31
**14**	136.3	5.63	**36**	18.7	1.39^{R} 1.61^{S}
**15**	131.4	5.42	**37**	35.9	1.84^{R} 1.51^{S}
**16**	79.1	4.51	**38**	60.3	3.62^{S} 3.53^{R}
**17**	30.6	2.14^{R} 1.54^{S}	**39**	10.7	0.88
**18**	37.3	2.04^{S} 1.80^{R}	**40**	16.2	1.01
**19**	105.7	-	**41**	112.5	5.39 5.02
**20**	26.4	1.47 1.32	**42**	15.9	0.97
**21**	27.1	1.81^{S} 1.72^{R}	**43**	23.1	1.73
**22**	69.7	3.57	**44**	27.3	1.36

Figure S1. ^{1}H NMR spectrum of okadaic acid in CD_3OD.

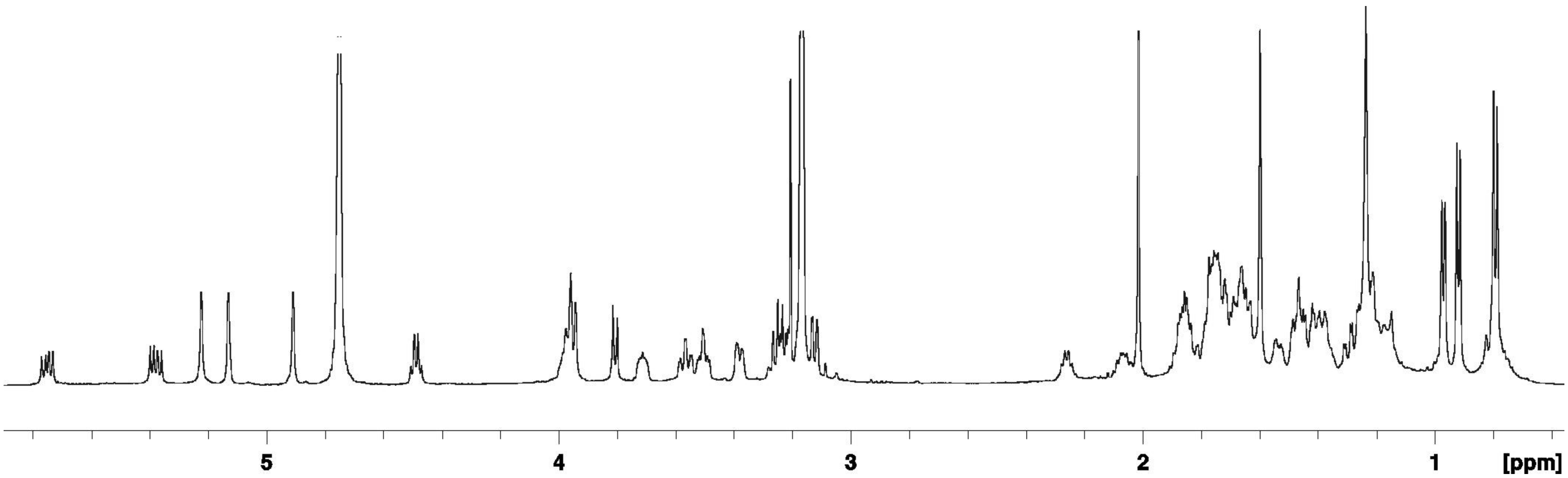

Figure S2. ^{13}C NMR spectrum of okadaic acid in CD_3OD.

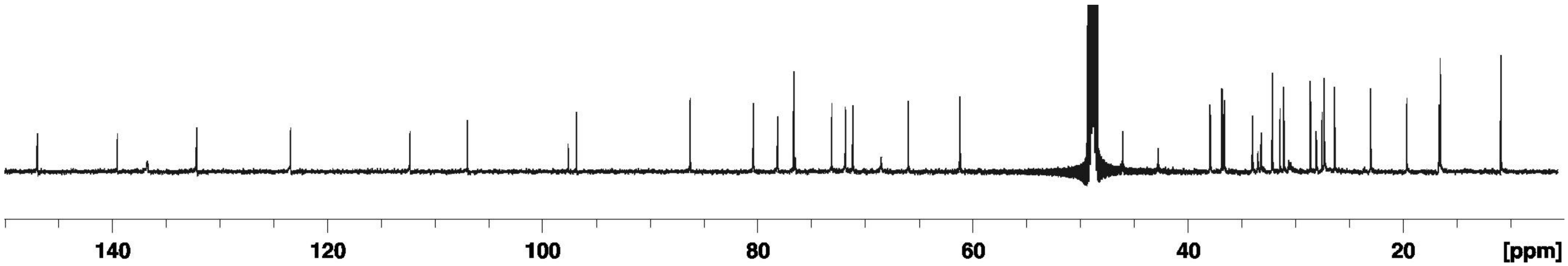

Figure S3. ROESY spectrum of okadaic acid in CD_3OD.

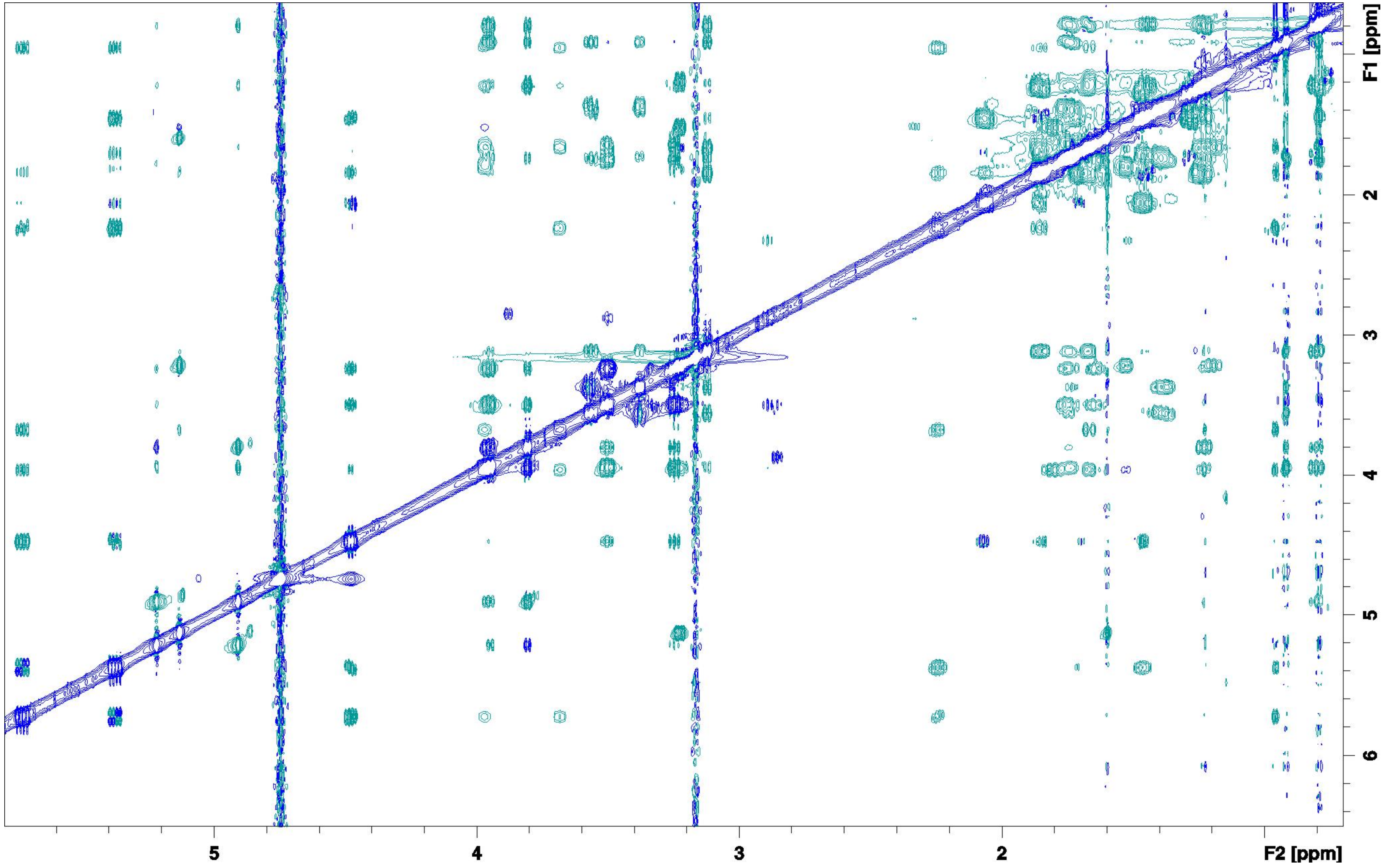

Figure S4. HSQC spectrum of okadaic acid in CD_3OD.

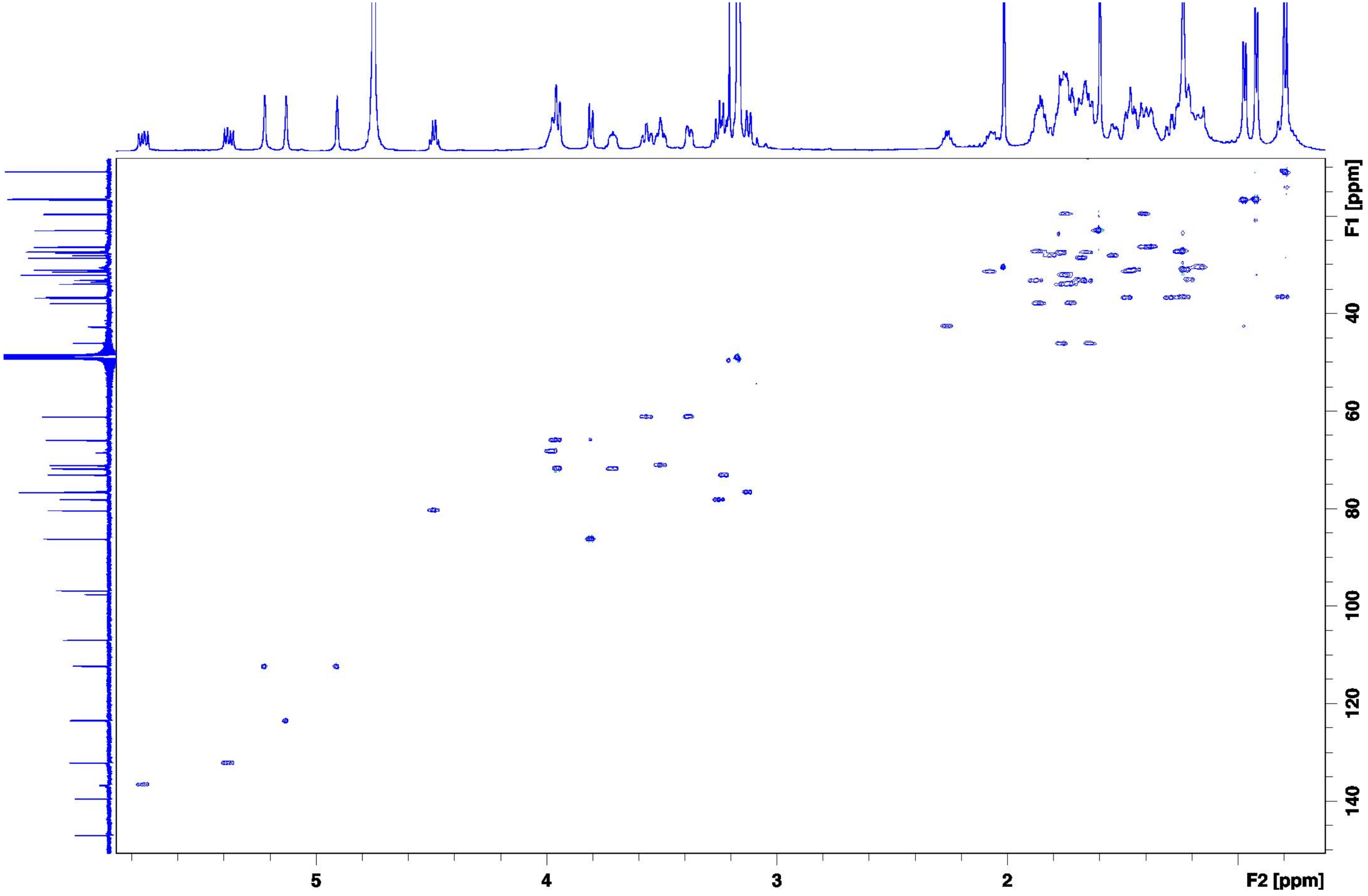

Figure S5. 1D-TOCSY spectrum of okadaic acid in CD_3OD with selective excitation (*purple*) H-14 (δ_H 5.81), (*green*) H-15 (δ_H 5.34) and (*red*) H-16 (δ_H 4.52).

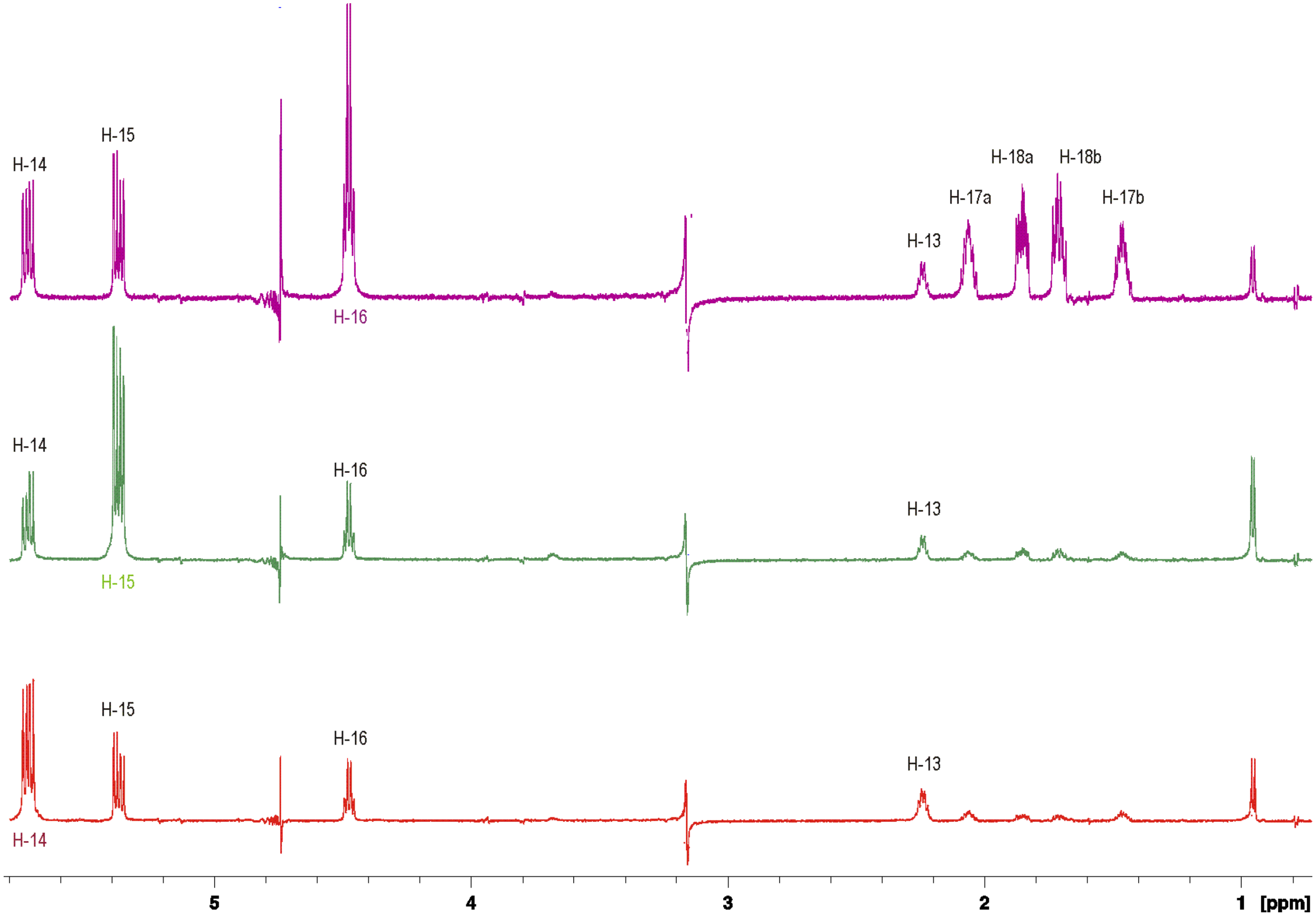

Figure S6. 1D-TOCSY spectrum of okadaic acid in CD_3OD with selective excitation (*purple*) H-27/H-24 (δ_H 3.94/3.92), (*green*) H-26 (δ_H 3.80) and (*red*) H-12 (δ_H 3.71).

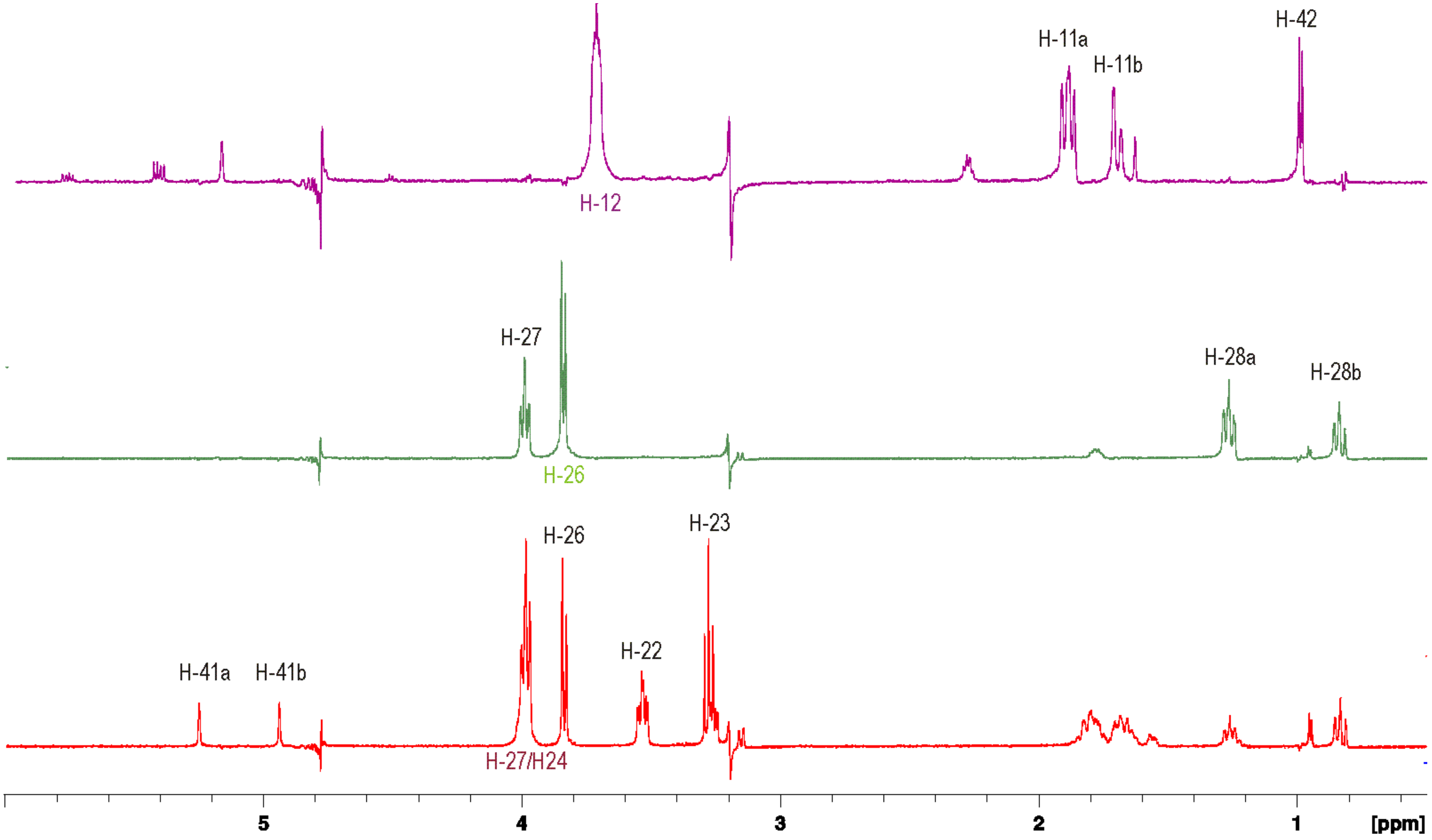

Figure S7. 1D-TOCSY spectrum of okadaic acid in CD_3OD with selective excitation (*purple*) H-38α (δ_H 3.57), (*green*) H-22 (δ_H 3.50) and (*red*) H-38β (δ_H 3.39).

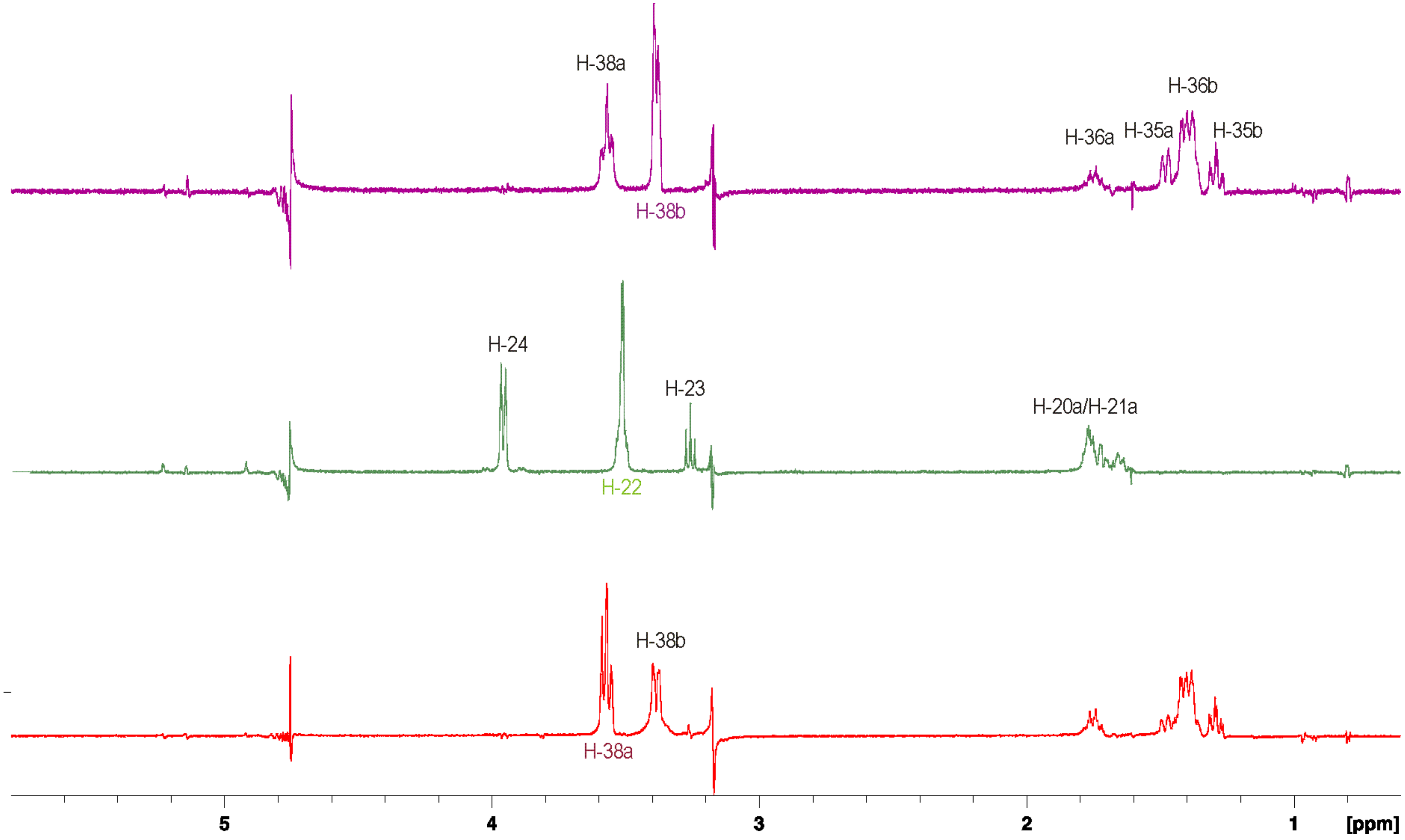

Figure S8. 1D-TOCSY spectrum of okadaic acid in CD_3OD with selective excitation (*purple*) H-30 (δ_H 3.13), (*green*) H-13 (δ_H 2.20) and (*red*) H-17α (δ_H 2.04).

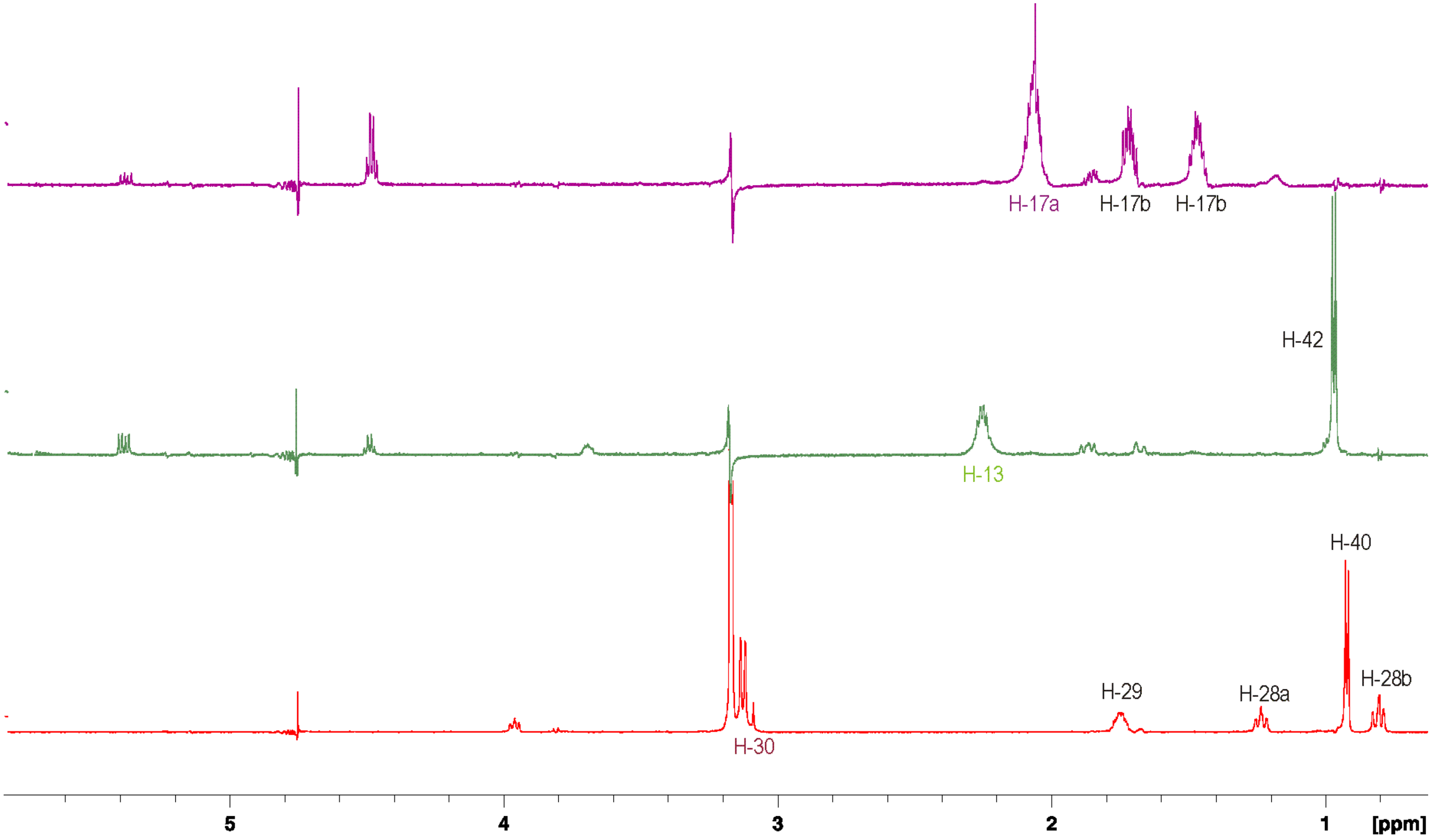

Table S3. Computed ^{13}C and ^{1}H chemical shifts for okadaic acid (**1**) and the studied diasteroisomer (**2**) on B3LYP-6-31G + (d).

C	1 (Gas)	1 ($CHCl_3$)	1 (CH_3OH)	2 (Gas)	2 ($CHCl_3$)	2 (CH_3OH)	CS I	CS II
**1**	14.1	13.3	13.4	23.4	23.0	21.2	7.8	14.3
**2**	114.1	114.0	114.0	116.6	115.7	115.5	114.9	111.5
**3**	147.4	147.5	147.8	145.5	145.9	146.2	143.6	146.8
**4**	123.3	122.7	122.7	121.8	122.1	123.0	122.6	119.2
**5**	158.1	158.1	158.3	157.1	157.4	157.7	155.6	155.7
**6**	158.0	158.1	158.1	152.9	152.8	152.9	157.6	160.7
**7**	117.1	118.0	118.1	119.0	119.3	119.8	117.6	119.4
**8**	95.9	96.5	96.4	97.2	97.8	99.3	90.9	89.7
**9**	67.9	71.3	72.7	70.1	72.6	75.1	68.2	69.9
**10**	53.6	51.0	49.2	55.9	52.2	51.3	54.5	51.5
**11**	154.8	154.8	154.8	156.4	156.9	157.0	154.5	154.5
**12**	120.0	120.6	120.3	122.6	121.8	123.2	120.8	118.3
**13**	143.3	143.8	144.0	142.6	143.3	143.3	142.4	146.0
**14**	55.5	55.0	53.8	60.0	58.6	59.5	51.6	57.1
**15**	57.5	58.4	59.2	54.7	55.2	56.8	62.5	61.6
**16**	113.7	114.0	114.2	115.6	115.3	115.9	112.5	115.4
**17**	158.6	158.7	159.1	158.8	159.2	159.4	157.4	156.5
**18**	152.7	152.6	152.6	155.0	155.1	155.2	151.7	151.0
**19**	89.1	89.2	89.2	88.2	88.5	88.7	90.4	94.5
**20**	153.4	153.9	154.0	152.5	152.9	153.0	152.4	153.8
**21**	162.8	163.1	163.2	161.2	161.4	161.4	160.2	162.1
**22**	117.8	117.9	118.2	117.5	120.1	120.6	117.9	116.6
**23**	114.8	115.1	115.1	117.3	117.9	117.7	114.4	114.4
**24**	117.5	118.0	117.9	117.5	117.6	117.3	115.5	115.9
**25**	39.9	40.8	41.4	46.1	44.6	43.2	47.6	33.3
**26**	103.0	103.7	103.9	100.6	101.6	101.3	105.0	105.0
**27**	127.0	127.0	127.1	118.1	119.2	119.1	123.6	112.8
**28**	155.4	155.3	155.4	149.9	148.8	148.2	154.7	152.0
**29**	155.6	155.6	155.5	146.7	147.1	147.3	154.9	152.3
**30**	116.1	116.0	116.0	117.9	115.9	115.9	113.3	117.3
**31**	159.3	159.5	159.7	153.9	153.8	153.9	159.1	160.0
**32**	162.5	162.4	162.5	164.0	163.0	163.1	162.6	161.6
**33**	157.5	157.4	157.5	158.5	158.7	158.7	157.5	157.2
**34**	99.1	98.6	98.5	98.0	97.5	97.5	98.5	100.0
**35**	151.2	150.9	150.9	150.9	150.9	150.8	150.4	152.3
**36**	167.3	167.3	167.4	166.3	165.7	165.8	168.0	167.3
**37**	161.8	161.4	161.5	162.3	161.6	161.6	163.7	162.5
**38**	129.3	129.4	129.3	131.4	131.1	130.9	129.9	129.3
**39**	178.1	178.4	178.6	174.9	175.0	175.0	175.7	175.6
**40**	173.7	174.2	174.4	173.9	174.9	174.7	173.8	172.8
**41**	81.3	80.8	80.3	75.5	74.4	74.9	83.7	83.6
**42**	172.9	173.4	173.5	174.4	175.4	175.2	172.4	174.2
**43**	166.7	167.0	167.3	167.0	167.7	167.8	165.6	166.1
**44**	164.5	164.1	164.2	167.0	167.8	168.6	162.8	164.2

Table S3. *Cont.*

H	1 (Gas)	1 ($CHCl_3$)	1 (CH_3OH)	2 (Gas)	2 ($CHCl_3$)	2 (CH_3OH)	CS I	CS II
**H3**	30.77	30.60	30.56	30.62	30.62	30.71	30.33	31.23
**H3**	30.36	30.32	30.29	30.76	30.57	30.17	29.97	29.64
**H4**	27.94	27.86	27.83	28.38	27.99	28.28	28.55	27.39
**H5**	30.75	30.79	30.79	31.02	30.85	30.93	30.63	31.17
**H5**	31.04	30.79	30.70	30.23	30.20	30.19	30.75	29.73
**H6**	30.50	30.49	30.50	30.33	30.59	30.65	30.31	30.27
**H6**	30.02	30.06	30.11	30.32	30.07	30.33	30.13	29.98
**H7**	28.77	28.46	28.35	29.02	28.80	28.94	28.88	28.81
**H9**	26.40	26.48	26.55	26.48	26.26	26.56	26.39	26.66
**H11**	30.66	30.53	30.39	30.49	30.36	30.13	30.49	30.46
**H11**	30.54	30.35	30.24	30.76	30.40	30.60	30.77	30.79
**H12**	28.42	28.75	28.71	28.10	27.89	27.78	27.93	28.05
**H13**	30.54	30.30	30.16	30.70	29.96	30.39	30.45	30.00
**H14**	26.85	26.85	26.73	26.62	26.51	26.66	25.90	25.25
**H15**	27.02	26.91	26.82	26.37	26.06	26.56	26.70	26.54
**H16**	27.61	27.61	27.68	27.92	27.90	27.98	27.73	27.65
**H17**	30.72	30.54	30.53	30.28	30.23	30.30	30.47	30.48
**H17**	30.22	30.12	30.15	30.92	30.71	30.63	30.85	30.86
**H18**	30.13	30.19	30.26	30.22	30.15	30.20	30.24	30.14
**H18**	30.34	30.19	30.12	30.44	30.25	30.07	30.53	30.80
**H20**	30.35	30.27	30.20	30.45	30.41	30.33	30.84	30.70
**H20**	30.29	30.18	30.20	30.46	30.47	30.44	30.08	30.35
**H21**	30.41	30.32	30.26	29.89	30.15	30.21	30.40	30.86
**H21**	30.01	30.09	30.15	30.41	30.63	30.71	30.03	30.07
**H22**	28.59	28.56	28.44	27.45	27.75	27.71	28.23	28.28
**H23**	28.47	28.40	28.40	28.31	28.35	28.28	29.08	29.29
**H24**	27.70	27.74	27.63	27.99	27.93	27.72	27.47	26.89
**H26**	28.28	28.18	28.02	27.90	27.56	27.12	28.43	28.26
**H27**	27.84	27.75	27.67	26.19	26.30	26.15	27.20	28.15
**H28**	31.09	31.08	30.98	30.37	30.54	30.40	30.84	30.12
**H28**	31.47	31.56	31.53	31.41	31.15	30.88	30.81	30.75
**H29**	29.62	29.90	29.97	30.62	30.56	30.38	29.70	30.16
**H30**	28.63	28.63	28.58	28.89	28.54	28.53	28.51	28.23
**H31**	30.44	30.42	30.36	30.81	30.61	30.56	30.21	30.44
**H32**	31.11	30.97	30.93	31.41	31.19	31.26	30.84	31.25
**H32**	29.96	30.09	30.15	30.53	30.53	30.77	30.12	29.91
**H33**	30.65	30.52	30.48	30.71	30.59	30.52	30.66	30.82
**H33**	31.18	31.02	31.03	31.75	31.35	31.44	31.39	31.30
**H35**	30.67	30.65	30.66	30.14	30.43	30.36	30.72	30.70
**H35**	30.92	30.71	30.66	31.56	31.16	31.14	31.28	31.05
**H36**	30.90	30.83	30.79	29.52	29.82	29.76	30.04	30.00
**H36**	29.97	30.11	30.15	30.65	30.78	30.71	31.20	30.94
**H37**	30.76	30.82	30.78	31.06	30.70	30.76	31.12	30.80
**H37**	30.72	30.57	30.56	30.71	30.86	30.80	30.96	30.87
**H38**	28.26	28.36	28.33	28.36	28.45	28.41	28.27	28.38

Table S3. *Cont.*

H	1 (Gas)	1 ($CHCl_3$)	1 (CH_3OH)	2 (Gas)	2 ($CHCl_3$)	2 (CH_3OH)	CS I	CS II
**H38**	28.71	28.57	28.52	29.49	28.88	28.83	28.87	28.71
**H39**	31.22	31.24	31.25	31.01	31.02	30.95	30.99	31.11
**H40**	31.10	31.21	31.18	30.10	30.56	30.50	31.11	31.13
**H41**	27.52	27.28	27.11	27.71	27.44	27.15	27.00	26.86
**H41**	26.89	26.97	26.90	26.76	26.62	26.71	27.23	26.52
**H42**	31.41	31.34	31.30	31.35	31.54	31.18	31.18	31.17
**H43**	30.51	30.45	30.40	30.55	30.39	30.40	30.57	30.63
**H44**	30.92	30.88	30.87	30.70	30.80	30.78	31.01	30.80

Table S4. Computed ^{13}C and ^{1}H chemical shifts for okadaic acid (**1**) and the studied diasteroisomer (**2**) on mPW1Pw91-6-31G + (d).

H	1 (Gas)	1 ($CHCl_3$)	1 (CH_3OH)	2 (Gas)	2 ($CHCl_3$)	2 (CH_3OH)	CS I	CS II
**1**	16.6	16.3	16.1	26.0	26.7	25.7	10.3	17.3
**2**	118.5	118.5	118.4	121.2	120.4	120.3	119.4	116.1
**3**	152.4	152.7	152.7	150.7	150.8	151.0	148.4	152.3
**4**	127.8	127.4	127.5	127.2	127.1	127.3	128.2	124.2
**5**	163.2	163.3	163.3	162.2	162.5	162.7	160.7	160.7
**6**	162.7	162.9	163.0	158.7	158.6	158.7	163.4	167.1
**7**	122.1	122.9	123.1	124.0	124.4	124.8	123.2	124.8
**8**	100.7	101.0	101.3	102.4	101.9	102.5	96.3	94.7
**9**	70.8	74.0	75.7	72.9	75.5	76.7	70.2	72.2
**10**	57.1	54.3	52.7	59.2	55.5	53.8	58.3	55.3
**11**	159.5	159.6	159.5	161.3	161.8	161.9	159.2	159.0
**12**	124.9	125.0	125.2	127.6	126.4	126.5	125.8	123.7
**13**	148.8	149.2	149.4	148.7	149.4	149.7	148.5	151.6
**14**	59.1	58.5	57.2	63.2	61.4	60.8	55.3	60.0
**15**	61.0	61.6	62.5	58.2	58.4	59.1	65.0	64.0
**16**	118.5	118.7	118.6	120.8	120.6	120.6	117.0	119.7
**17**	163.3	163.6	163.8	163.4	163.9	164.2	162.2	161.1
**18**	156.9	157.0	157.0	158.9	159.2	159.3	156.6	156.0
**19**	94.2	94.2	94.1	93.5	93.7	93.4	95.4	99.1
**20**	158.2	158.6	158.6	156.5	156.9	157.1	157.2	159.5
**21**	167.4	167.7	167.9	166.3	166.4	166.4	165.2	166.6
**22**	123.1	123.1	123.2	123.0	124.8	125.0	123.0	121.4
**23**	119.3	119.7	119.7	122.1	122.5	122.4	118.9	119.7
**24**	122.3	122.5	122.7	122.1	122.6	122.3	120.9	120.9
**25**	43.8	44.7	45.5	49.7	49.1	49.0	51.6	37.9
**26**	108.0	108.6	109.1	106.1	107.1	107.1	109.7	110.6
**27**	131.5	131.5	131.6	123.4	124.4	124.5	128.5	117.9
**28**	160.4	160.4	160.6	154.6	153.8	153.6	160.2	157.5
**29**	160.8	160.8	160.7	152.0	152.6	152.9	159.8	157.8
**30**	121.1	121.0	121.0	122.2	120.6	120.5	118.8	122.5
**31**	164.7	164.9	165.1	159.7	159.7	159.8	164.9	165.4

Table S4. *Cont.*

H	1 (Gas)	1 ($CHCl_3$)	1 (CH_3OH)	2 (Gas)	2 ($CHCl_3$)	2 (CH_3OH)	CS I	CS II
**32**	167.6	167.6	167.7	169.1	168.3	168.4	167.8	166.7
**33**	162.6	162.6	162.6	163.7	164.1	164.1	162.4	162.2
**34**	103.6	103.2	103.2	102.9	102.6	102.6	103.0	104.2
**35**	156.3	156.3	156.2	155.7	155.9	155.9	156.2	157.3
**36**	172.0	172.0	172.1	170.6	170.2	170.2	172.7	171.8
**37**	166.8	166.6	166.6	167.4	166.8	166.9	168.9	167.5
**38**	134.0	134.0	133.9	135.2	135.1	135.0	134.3	134.0
**39**	182.2	182.6	182.8	178.5	178.9	179.1	180.1	179.1
**40**	177.3	177.9	178.2	177.6	178.4	178.4	177.7	176.9
**41**	83.2	82.6	82.1	78.0	76.6	76.6	84.1	85.1
**42**	176.8	177.3	177.5	178.4	179.2	179.2	176.0	178.2
**43**	170.3	170.7	170.9	170.9	171.5	171.8	168.8	169.4
**44**	169.2	169.1	169.0	171.5	172.1	172.5	167.0	168.0
**H3**	30.71	30.55	30.47	30.56	30.40	30.39	30.22	31.10
**H3**	30.36	30.32	30.31	30.70	30.59	30.47	29.97	29.68
**H4**	27.81	27.72	27.77	28.28	27.75	27.76	28.42	27.40
**H5**	30.66	30.69	30.73	31.02	30.77	30.76	30.59	31.10
**H5**	30.97	30.73	30.61	30.39	30.32	30.30	30.83	29.78
**H6**	30.45	30.43	30.41	30.34	30.52	30.56	30.25	30.18
**H6**	29.95	29.97	30.02	30.23	29.96	30.02	30.00	30.02
**H7**	28.66	28.34	28.24	29.00	28.71	28.68	28.70	28.68
**H9**	26.30	26.36	26.42	26.32	26.16	26.14	26.28	26.51
**H11**	30.56	30.42	30.36	30.49	30.31	30.17	30.42	30.35
**H11**	30.47	30.29	30.17	30.63	30.27	30.27	30.68	30.70
**H12**	28.46	28.68	28.76	28.13	27.92	27.85	27.96	28.14
**H13**	30.29	30.07	29.95	30.28	29.58	29.58	30.26	29.95
**H14**	26.79	26.72	26.64	26.66	26.36	26.24	25.88	25.37
**H15**	26.80	26.69	26.69	26.08	25.76	25.78	26.43	26.41
**H16**	27.56	27.58	27.55	27.81	27.71	27.71	27.69	27.62
**H17**	30.64	30.49	30.41	30.24	30.08	30.07	30.42	30.41
**H17**	30.20	30.11	30.06	30.69	30.55	30.50	30.71	30.77
**H18**	30.08	30.12	30.15	30.24	30.12	30.13	30.15	30.06
**H18**	30.29	30.13	30.01	30.35	30.25	30.16	30.41	30.72
**H20**	30.30	30.22	30.11	30.38	30.32	30.25	30.79	30.63
**H20**	30.24	30.15	30.08	30.40	30.39	30.36	30.03	30.33
**H21**	30.34	30.24	30.14	29.93	30.06	30.05	30.38	30.70
**H21**	29.90	29.99	30.02	30.37	30.52	30.57	29.90	30.06
**H22**	28.54	28.49	28.35	27.54	27.71	27.63	28.34	28.31
**H23**	28.27	28.22	28.20	28.26	28.27	28.21	28.92	29.12
**H24**	27.63	27.61	27.54	27.87	27.84	27.72	27.51	27.10
**H26**	28.18	28.03	27.98	27.74	27.57	27.39	28.11	28.10
**H27**	27.79	27.72	27.64	26.28	26.37	26.28	27.32	28.22
**H28**	31.08	31.04	31.03	30.35	30.53	30.47	30.78	30.01

Table S4. *Cont.*

H	1 (Gas)	1 ($CHCl_3$)	1 (CH_3OH)	2 (Gas)	2 ($CHCl_3$)	2 (CH_3OH)	CS I	CS II
**H28**	31.45	31.54	31.56	31.40	31.23	31.14	30.81	30.69
**H29**	29.55	29.80	29.91	30.52	30.48	30.37	29.60	30.06
**H30**	28.57	28.58	28.56	28.77	28.47	28.48	28.48	28.20
**H31**	30.44	30.42	30.37	30.72	30.52	30.48	30.19	30.41
**H32**	31.06	30.92	30.86	31.37	31.11	31.09	30.77	31.17
**H32**	29.94	30.05	30.12	30.50	30.43	30.56	30.12	29.95
**H33**	30.62	30.50	30.45	30.70	30.64	30.57	30.68	30.72
**H33**	31.11	30.95	30.94	31.59	31.24	31.26	31.39	31.24
**H35**	30.63	30.62	30.62	30.19	30.48	30.45	30.65	30.65
**H35**	30.90	30.66	30.60	31.47	31.09	31.05	31.19	31.03
**H36**	30.85	30.77	30.73	29.53	29.87	29.88	29.97	30.06
**H36**	29.90	30.08	30.13	30.68	30.78	30.74	31.11	30.88
**H37**	30.73	30.79	30.75	30.96	30.59	30.60	31.05	30.80
**H37**	30.70	30.53	30.51	30.68	30.80	30.75	30.89	30.83
**H38**	28.23	28.35	28.33	28.43	28.49	28.46	28.22	28.43
**H38**	28.66	28.50	28.45	29.35	28.79	28.73	28.80	28.69
**H39**	31.18	31.20	31.22	31.00	31.03	31.01	30.95	31.03
**H40**	31.07	31.16	31.16	30.16	30.59	30.59	31.05	31.08
**H41**	27.33	27.09	26.99	27.50	27.29	27.12	26.84	26.76
**H41**	26.73	26.76	26.76	26.54	26.33	26.36	27.06	26.38
**H42**	31.34	31.26	31.23	31.35	31.56	31.49	31.13	31.08
**H43**	30.44	30.36	30.34	30.47	30.30	30.27	30.49	30.52
**H44**	30.86	30.82	30.78	30.67	30.73	30.67	31.01	30.75

S1. Correlation Graphs

B3LYP-6-31G + (d)

S1.1. Use of $CHCl_3$ as Solvent

1_{gas} (A); 2_{gas} (B); 1_{CHCl3} (C); 2_{CHCl3} (D); 1_{CH3OH} (E); 2_{CH3OH} (F); CS I (G) CS II (H).

S1.2. Use of CH_3OH as Solvent

1_{gas} (I); 2_{gas} (J); 1_{CH3OH} (K); 2_{CH3OH} (L); 1_{CHCl3} (M); 2_{CHCl3} (N); CS I (O); CS II (P).

Figure S9. Correlations graphs for calculated *vs.* experimental data.

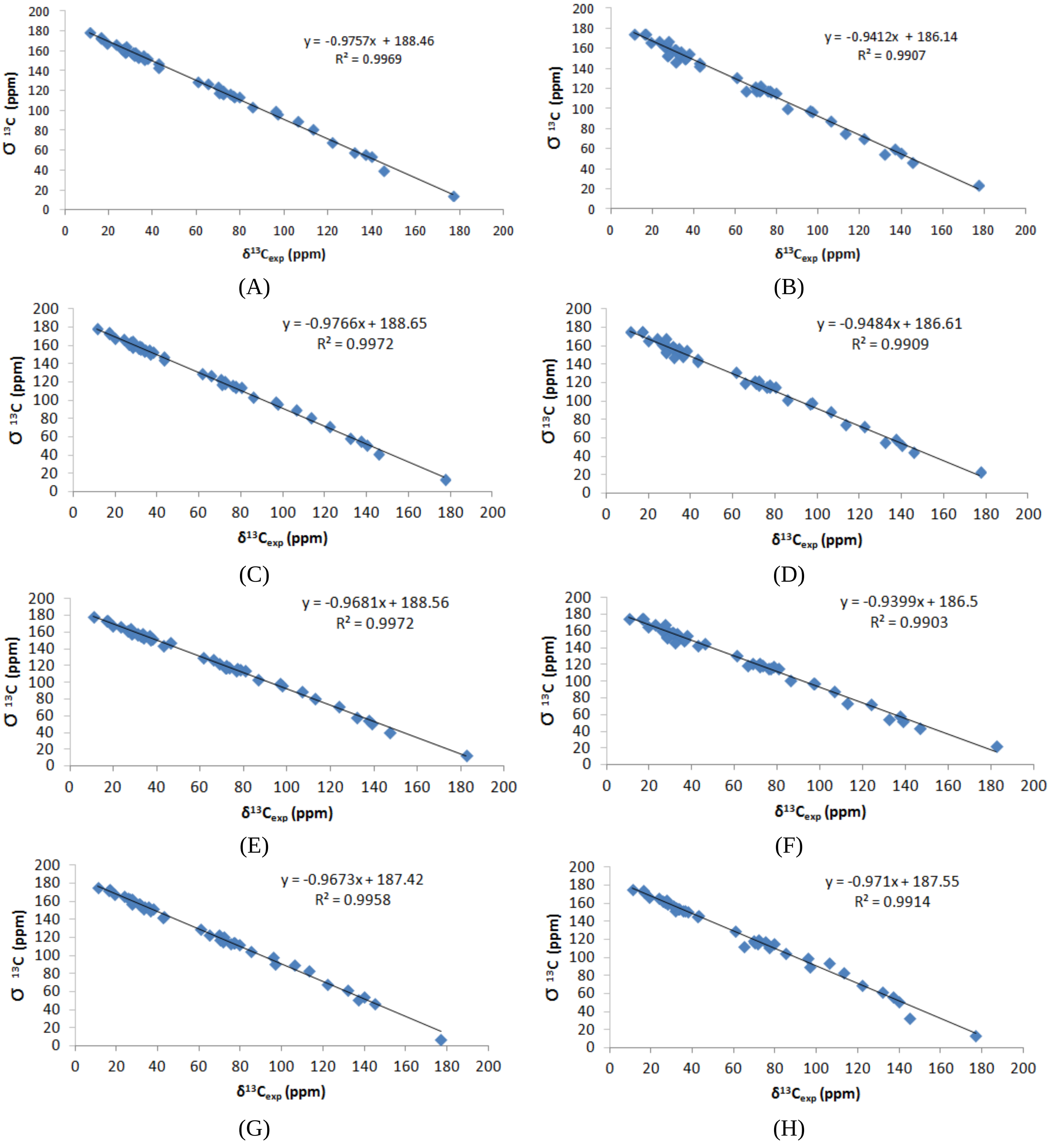

Figure S10. Correlations graphs for calculated *vs.* experimental data.

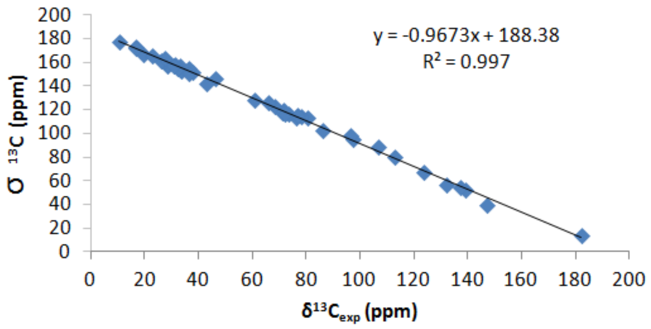

(I)

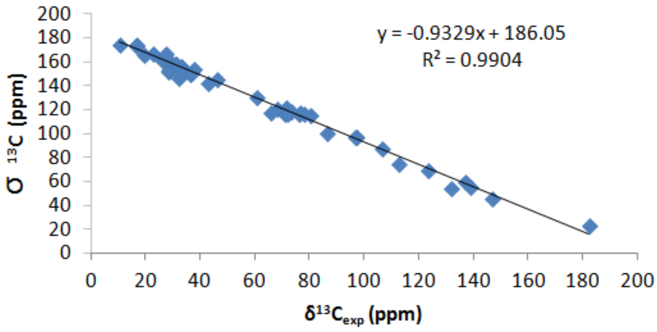

(J)

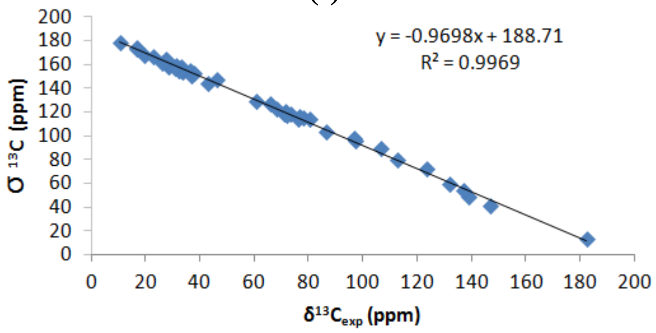

(K)

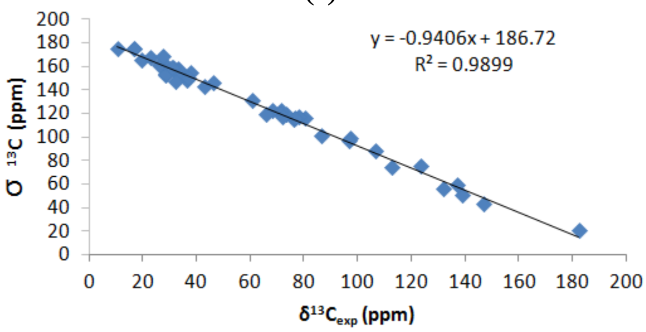

(L)

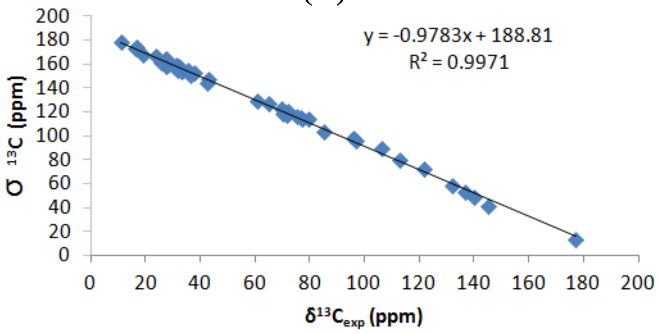

(M)

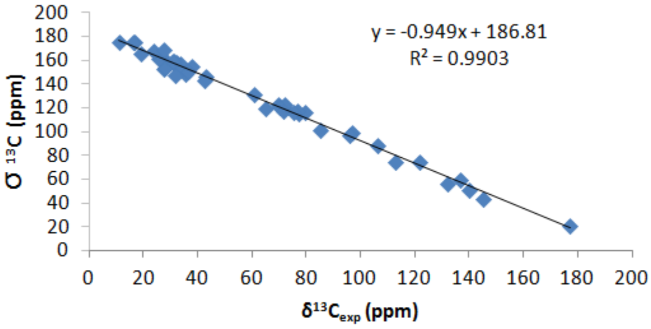

(N)

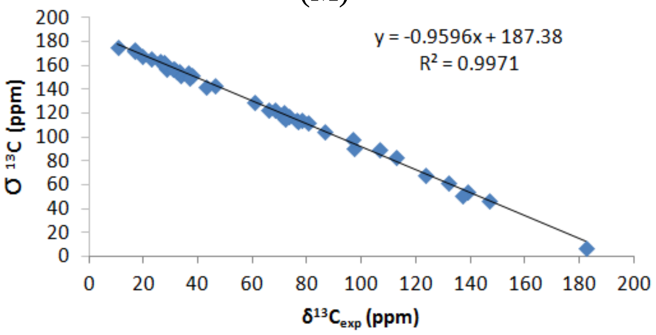

(O)

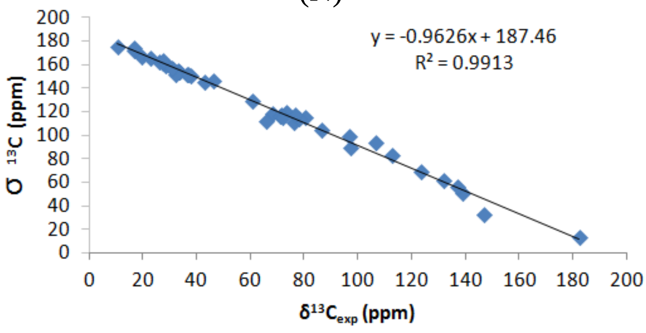

(P)

Figure S11. Correlations graphs for calculated *vs* experimental data.

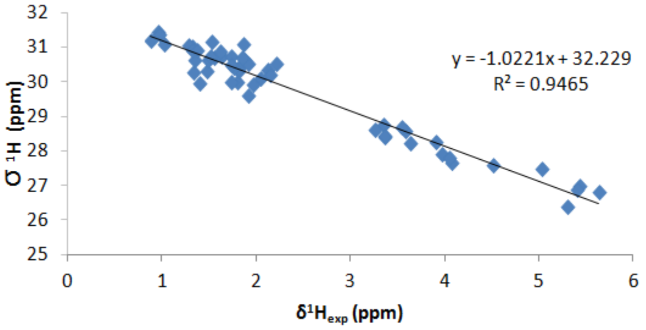

(A)

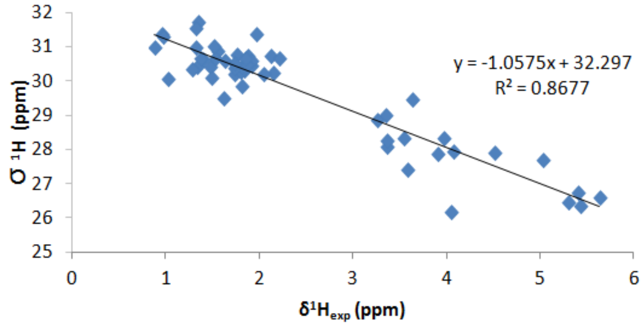

(B)

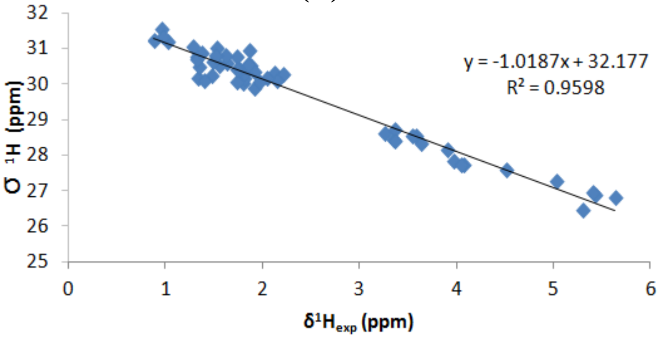

(C)

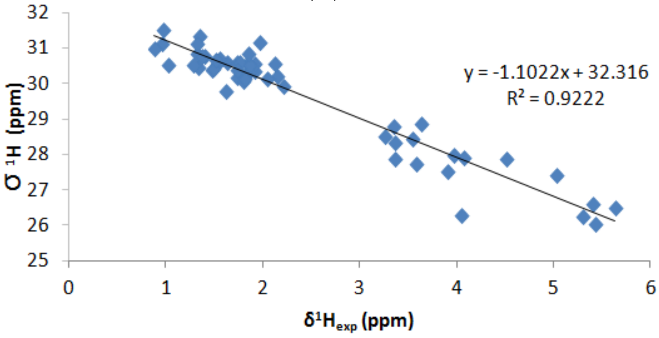

(D)

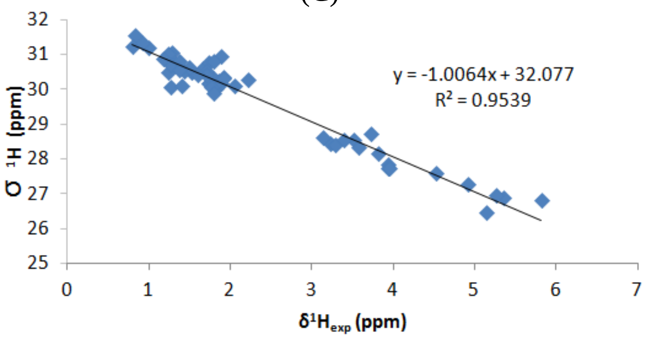

(E)

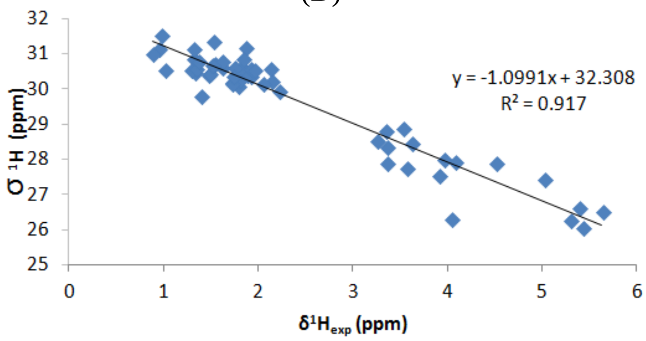

(F)

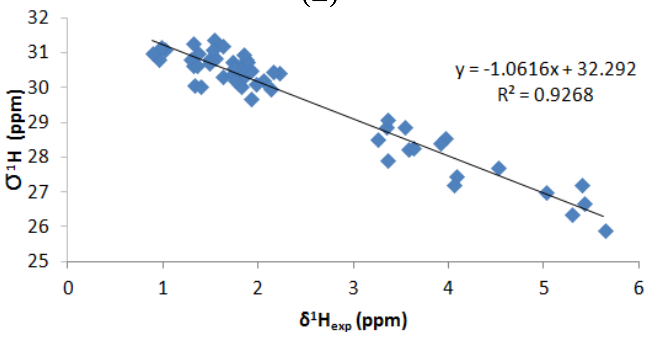

(G)

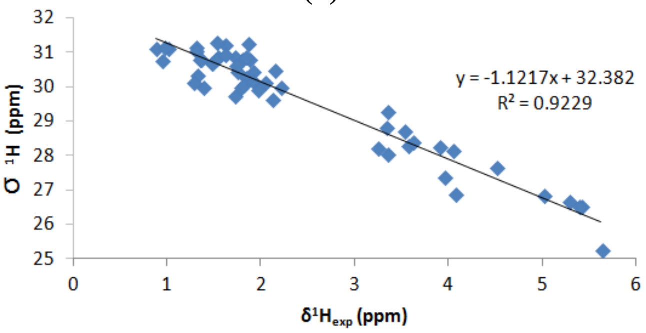

(H)

Figure S12. Correlations graphs for calculated *vs* experimental data.

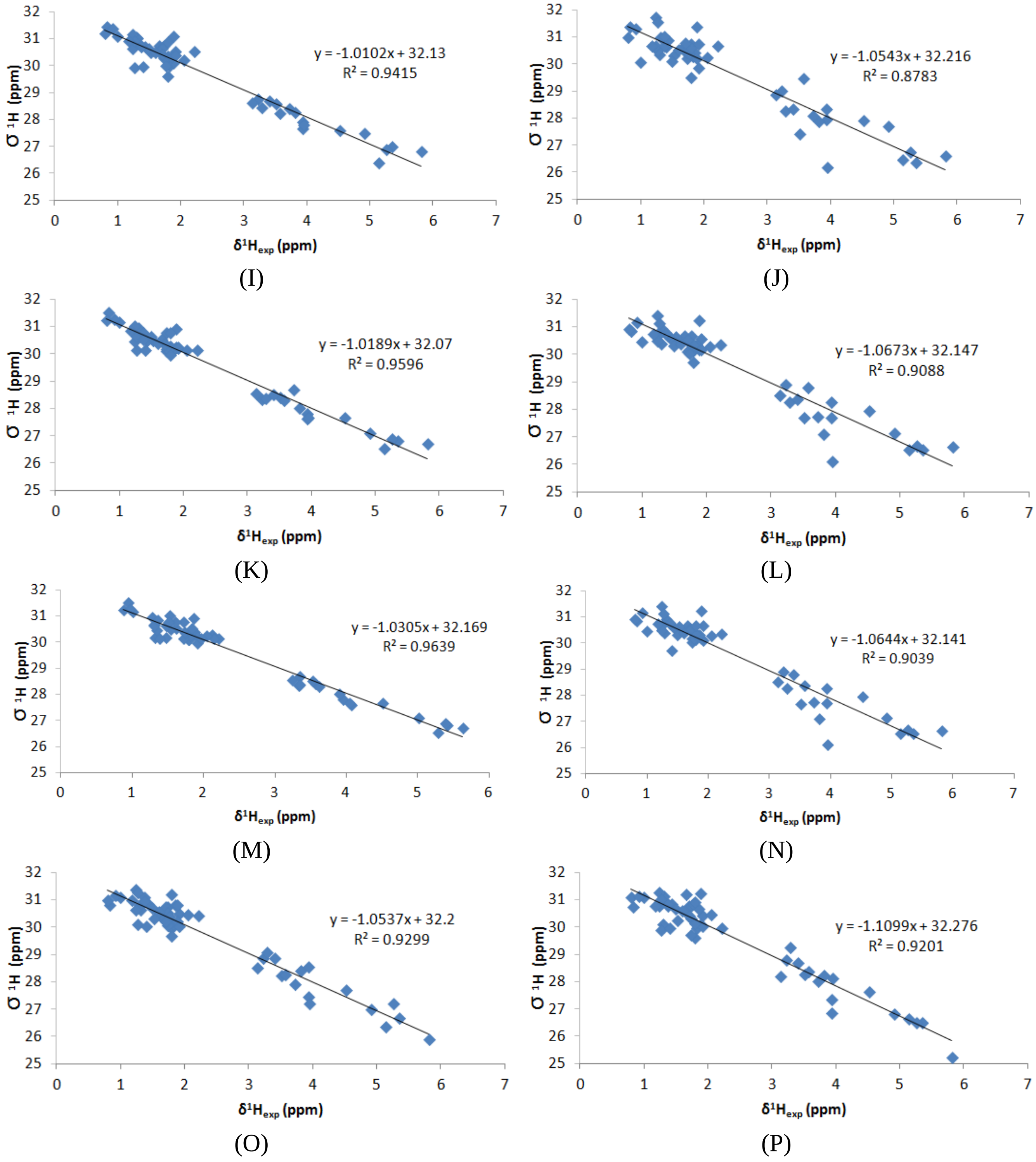

S2. Correlation Graphs

mPW1Pw91-6-31G + (d)

S2.1. Use of $CHCl_3$ as Solvent

1_{gas} (A); 2_{gas} (B); 1_{CHCl3} (C); 2_{CHCl3} (D); 1_{CH3OH} (E); 2_{CH3OH} (F); CS I (G) CS II (H)

S2.2. Use of CH_3OH as Solvent

1_{gas} (I); 2_{gas} (J); 1_{CH3OH} (K); 2_{CH3OH} (L); 1_{CHCl3} (M); 2_{CHCl3} (N); CS I (O); CS II (P)

Figure S13. Correlations graphs for calculated *vs*. experimental data.

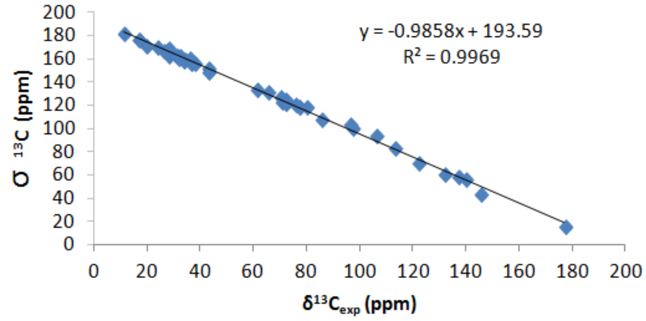

(A)

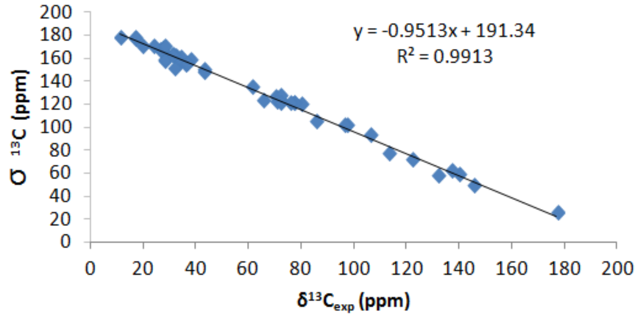

(B)

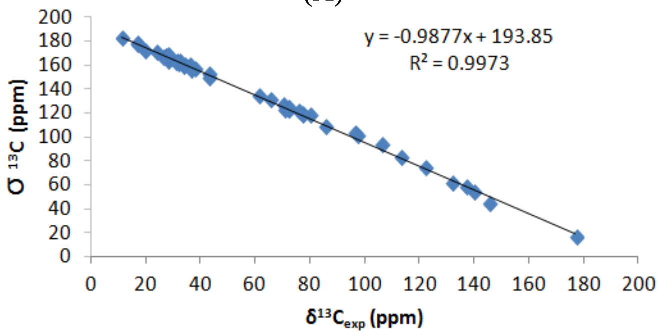

(C)

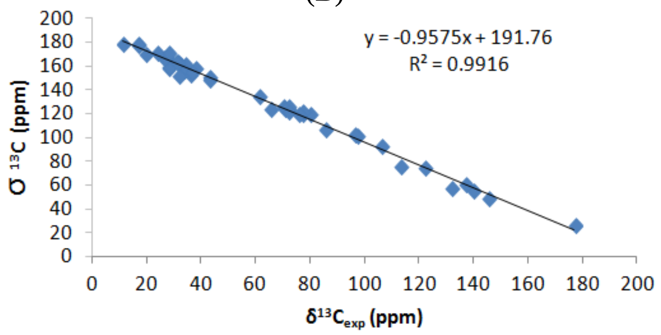

(D)

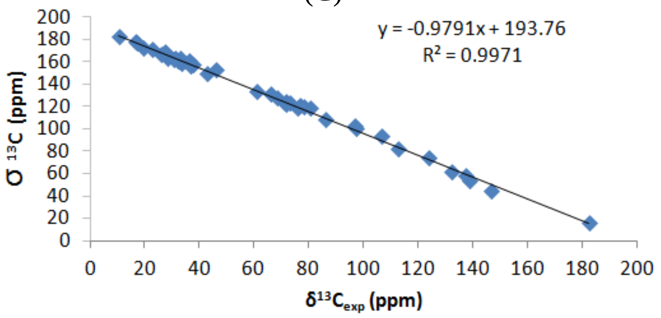

(E)

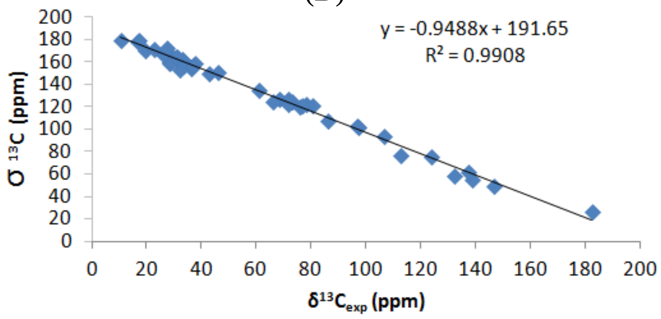

(F)

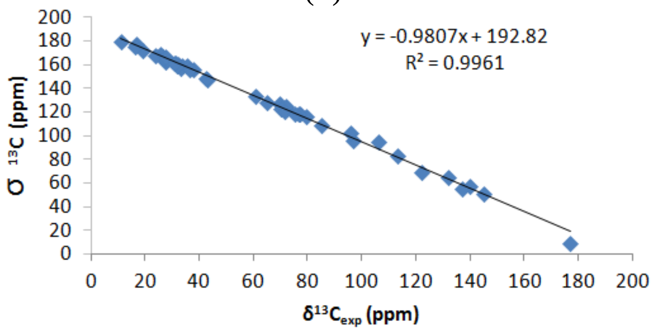

(G)

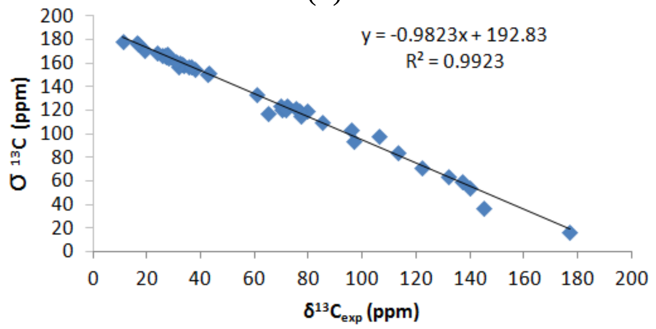

(H)

Figure S14. Correlations graphs for calculated *vs*. experimental data.

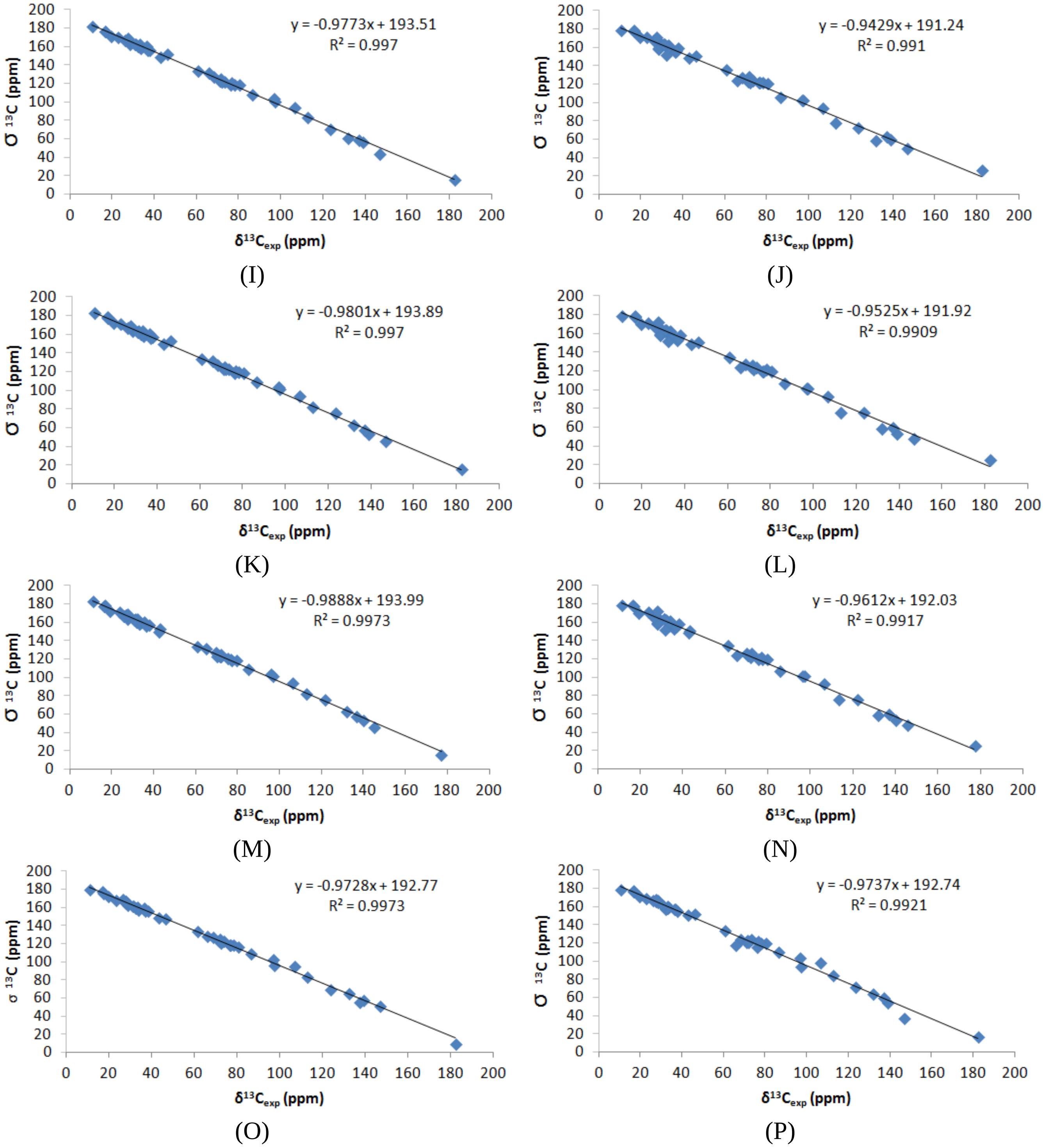

Figure S15. Correlations graphs for calculated *vs* experimental data.

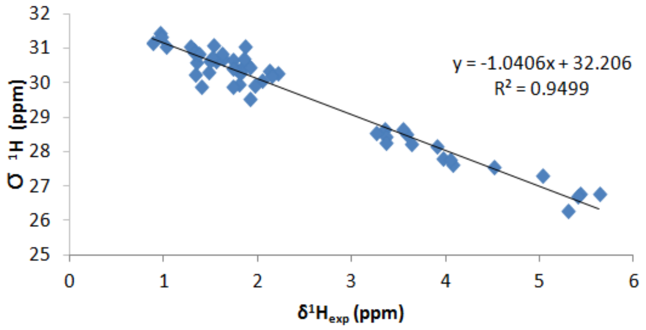

(A)

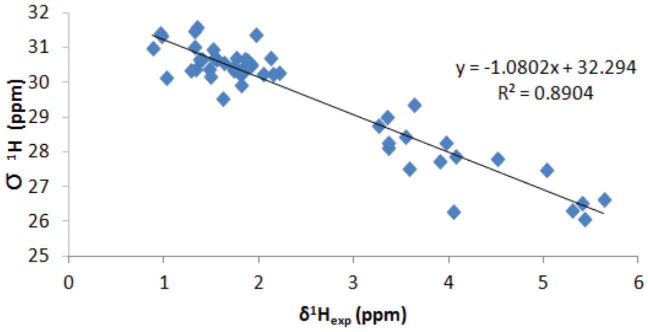

(B)

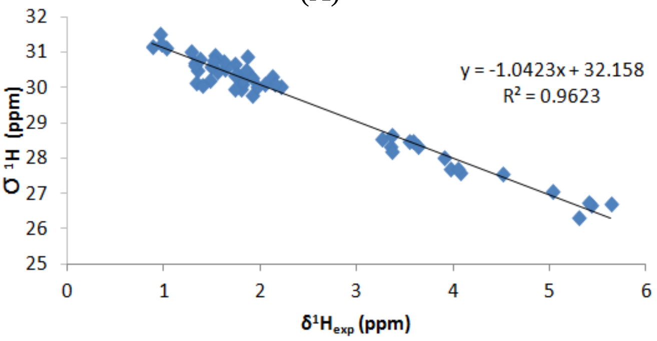

(C)

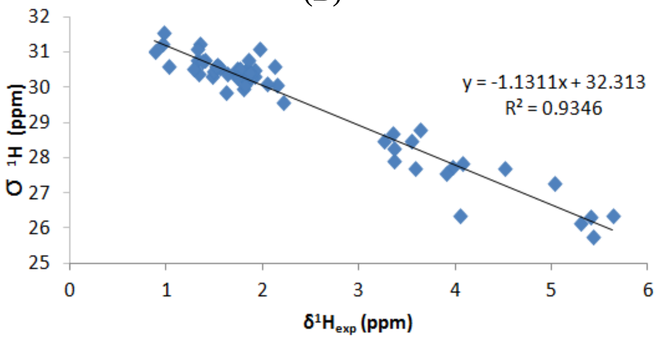

(D)

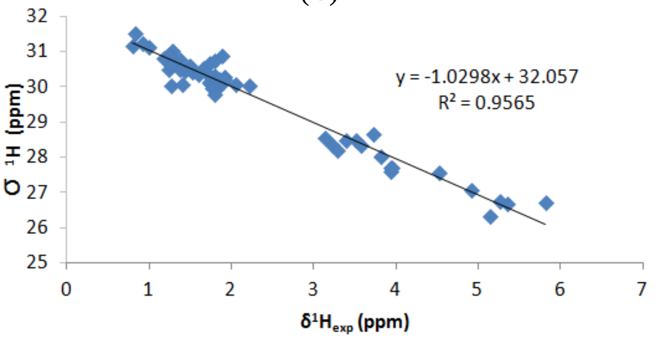

(E)

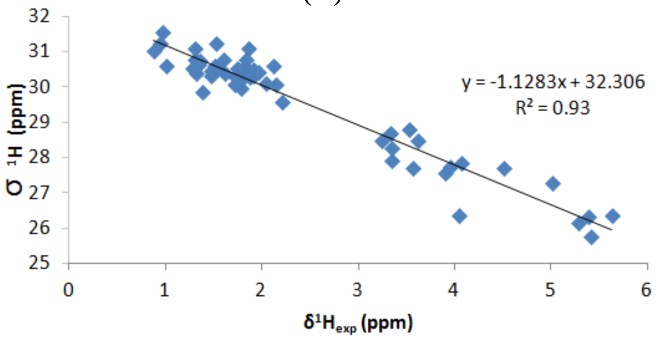

(F)

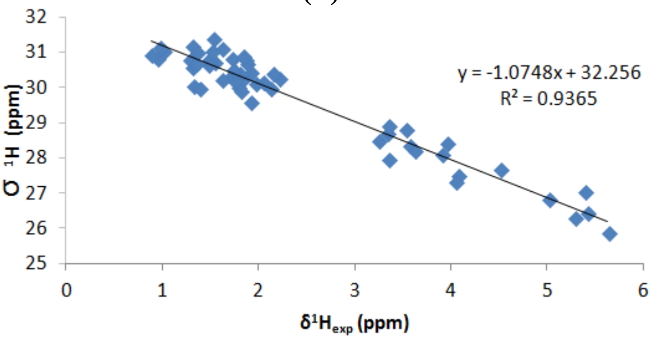

(G)

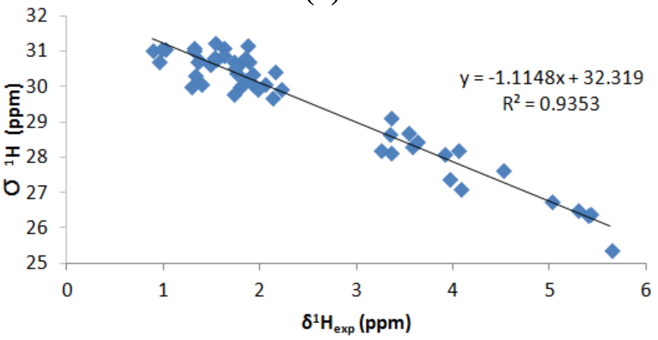

(H)

Figure S16. Correlations graphs for calculated *vs.* experimental data.

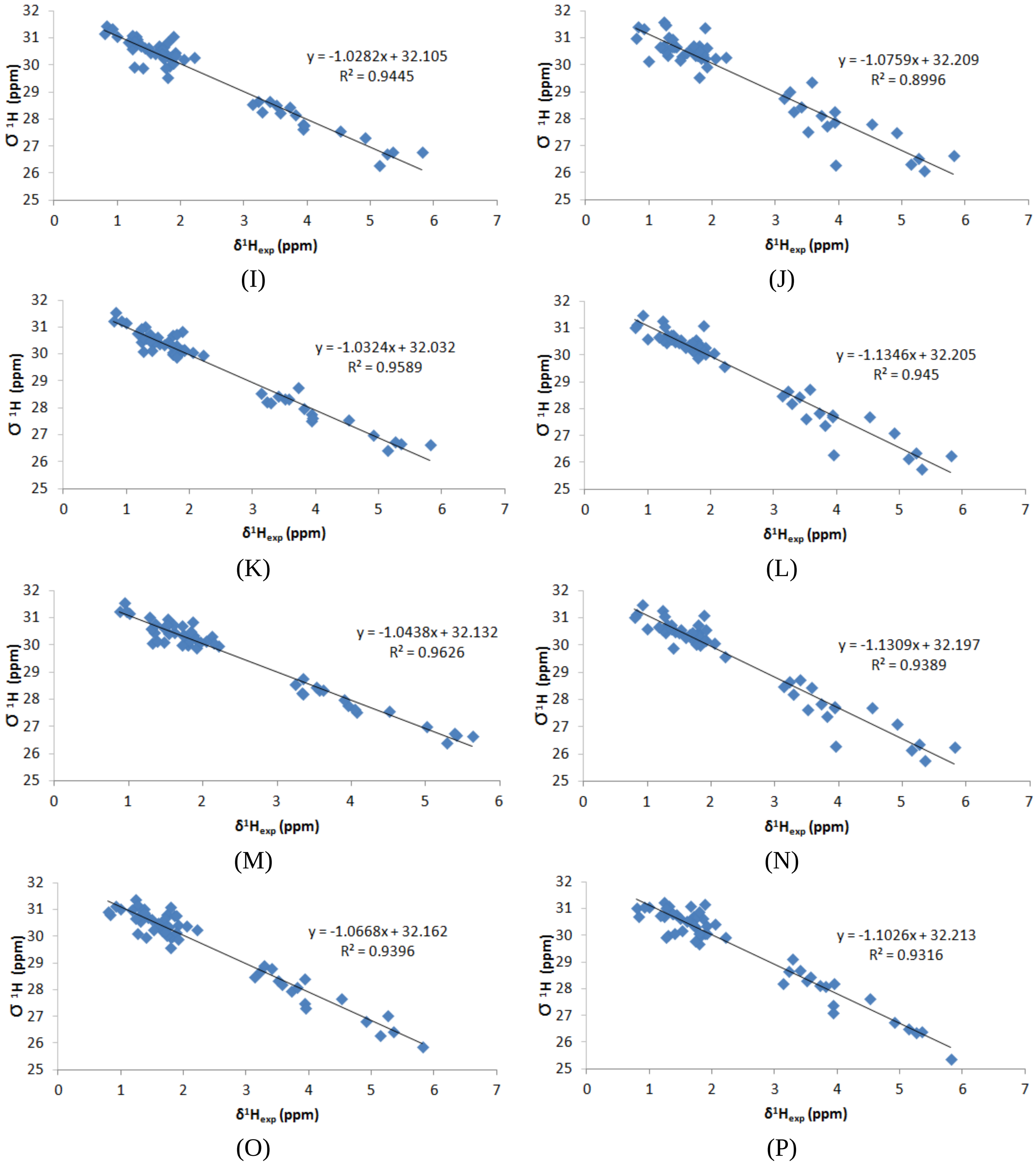

S3. Energies and Coordinates

Figure S17. Studied diasteroisomer of okadaic acid (**2**). Gas phase energy −2695.5434021131 hartrees.

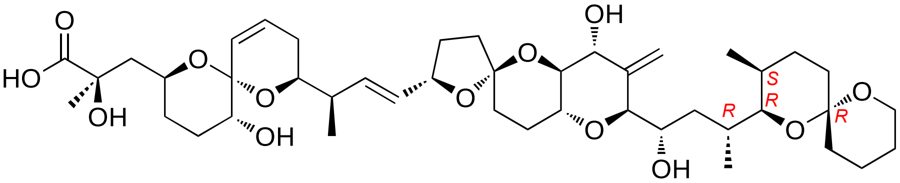

Okadaic acid 29-32 diasteroisomer (**2**)

Table S5. Structural coordinates.

Center		Coordinates		
Number		X	Y	Z
**1**	C1	84.111168	50.706711	25.452652
**2**	C2	84.569450	50.336964	26.878521
**3**	C3	86.049271	50.719624	27.141647
**4**	C4	86.453064	52.212521	27.066313
**5**	C5	87.971275	52.351387	27.286291
**6**	C6	88.352135	53.823807	27.406681
**7**	C7	87.549423	54.434280	28.561356
**8**	C8	86.036255	54.280807	28.270906
**9**	C9	85.213867	54.795223	29.442688
**10**	C10	84.194481	55.656734	29.293772
**11**	C11	83.793610	56.137955	27.913450
**12**	C12	84.414833	55.262589	26.803734
**13**	C13	84.379234	55.931839	25.412613
**14**	C14	85.175659	55.102440	24.425243
**15**	C15	86.354111	55.461567	23.893171
**16**	C16	87.152122	54.571793	22.969793
**17**	C17	87.600609	55.236305	21.668802
**18**	C18	88.942505	54.571815	21.368023
**19**	C19	89.148048	53.632244	22.561241
**20**	C20	90.616982	53.522858	23.026472
**21**	C21	90.721100	52.493526	24.163731
**22**	C22	90.101562	51.175564	23.693445
**23**	C23	88.641785	51.435474	23.283344
**24**	C24	87.954483	50.139030	22.836945
**25**	C25	88.170837	49.048931	23.886366
**26**	C26	89.607170	48.915943	24.396290
**27**	C27	90.552406	48.173565	23.417315
**28**	C28	90.155930	46.700756	23.161081
**29**	C29	90.807404	46.029861	21.923105
**30**	C30	90.094727	44.687622	21.596380
**31**	O31	90.680389	43.635422	22.364664

Table S5. *Cont.*

**Center**		**Coordinates**		
**Number**		**X**	**Y**	**Z**
**32**	C32	90.005676	42.407364	22.344997
**33**	C33	89.922409	41.904999	20.887642
**34**	C34	89.249680	42.966602	20.004866
**35**	C35	90.806839	41.412422	23.213621
**36**	C36	92.331757	45.860352	22.109474
**37**	C37	87.176369	48.296246	24.397663
**38**	C38	82.950027	56.149242	24.892761
**39**	C39	83.407585	56.218822	30.461561
**40**	C40	84.356705	48.832565	27.134996
**41**	O41	83.815460	52.026081	25.329220
**42**	O42	84.003120	49.896599	24.536442
**43**	O43	83.749283	50.999065	27.820791
**44**	O44	85.745148	52.920490	28.091942
**45**	O45	87.890564	55.793686	28.706728
**46**	O46	85.785103	55.034733	27.118515
**47**	O47	88.378242	54.193047	23.570438
**48**	O48	88.681152	52.362568	22.198915
**49**	O49	90.161705	50.197285	24.726454
**50**	O50	86.572433	50.410881	22.657864
**51**	O51	91.808601	48.210762	24.059958
**52**	C52	90.007942	44.299297	20.098160
**53**	O53	88.699608	42.490490	22.850510
**54**	H54	86.333282	50.339264	28.123478
**55**	H55	86.668495	50.171185	26.431437
**56**	H56	86.200981	52.633137	26.092199
**57**	H57	88.269814	51.824951	28.193848
**58**	H58	88.517792	51.885452	26.466759
**59**	H59	89.421394	53.918850	27.595942
**60**	H60	88.150208	54.341377	26.467031
**61**	H61	87.783318	53.913967	29.491819
**62**	H62	83.899315	54.301678	26.762352
**63**	H63	84.864586	56.906067	25.489885
**64**	H64	86.575798	53.670700	22.746561
**65**	H65	86.872444	55.090221	20.870790
**66**	H66	87.742798	56.308842	21.804970
**67**	H67	88.954659	54.012646	20.431881
**68**	H68	89.736809	55.318512	21.344118
**69**	H69	91.229828	53.219292	22.176924
**70**	H70	90.965218	54.503040	23.353600
**71**	H71	91.763275	52.346546	24.446087
**72**	H72	90.201996	52.863209	25.048258
**73**	H73	90.669670	50.833321	22.828732
**74**	H74	88.084137	51.843391	24.128347

Table S5. *Cont.*

**Center**		**Coordinates**		
**Number**		**X**	**Y**	**Z**
**75**	H75	88.384071	49.802094	21.892485
**76**	H76	89.579605	48.347851	25.328142
**77**	H77	90.613678	48.695683	22.463161
**78**	H78	90.337334	46.099056	24.052914
**79**	H79	89.077415	46.675266	23.011967
**80**	H80	90.642036	46.695164	21.075048
**81**	H81	89.058197	44.787342	21.921312
**82**	H82	90.927528	41.681141	20.530481
**83**	H83	89.357536	40.972553	20.866545
**84**	H84	92.839638	46.823589	22.135321
**85**	H85	92.554741	45.347065	23.045341
**86**	H86	92.795830	45.284130	21.314009
**87**	H87	82.959930	56.591686	23.896093
**88**	H88	82.400185	55.209396	24.835022
**89**	H89	82.388596	56.824135	25.538750
**90**	H90	82.352356	55.960987	30.367887
**91**	H91	83.769310	55.825420	31.412176
**92**	H92	83.494972	57.305283	30.488779
**93**	H93	84.596390	48.567059	28.164793
**94**	H94	83.320381	48.542191	26.956467
**95**	H95	84.983955	48.223568	26.483643
**96**	H96	83.528610	52.162209	24.441505
**97**	H97	83.975571	51.920898	27.756470
**98**	H98	87.388588	56.241985	28.036694
**99**	H99	86.142975	49.626049	22.352432
**100**	H100	91.847290	49.044250	24.515341
**101**	H101	82.706703	56.129211	27.827446
**102**	H102	84.757889	54.142712	24.166992
**103**	H103	86.791862	56.417255	24.140842
**104**	H104	87.375031	47.558117	25.160955
**105**	H105	86.149849	48.397522	24.078188
**106**	H106	89.403000	45.053524	19.592585
**107**	C107	91.353195	44.241768	19.352644
**108**	H108	89.198418	42.622536	18.971785
**109**	H109	88.217789	43.105591	20.330845
**110**	H110	91.798187	45.232716	19.266016
**111**	H111	92.069412	43.594204	19.858271
**112**	H112	91.224106	43.864391	18.338161
**113**	C113	90.790962	41.871494	24.677481
**114**	H114	91.828842	41.353699	22.837666
**115**	C115	88.588509	42.945984	24.199028
**116**	H116	91.304405	42.829464	24.774235
**117**	C117	89.340828	42.001106	25.145294

Table S5. *Cont.*

**Center**		**Coordinates**		
**Number**		**X**	**Y**	**Z**
**118**	H118	91.329857	41.159924	25.303045
**119**	H119	88.973053	43.962822	24.286367
**120**	H120	87.533424	42.986008	24.469910
**121**	H121	88.858932	41.022606	25.150194
**122**	H122	89.301437	42.378227	26.167719
**123**	H123	90.361908	40.421181	23.118170
**124**	H124	85.510338	54.457653	30.424559
**125**	H125	84.115982	57.174664	27.811586

S4. Representative NMR Compatible Conformational Search (CS I)

Figure S18. Crystallographic structure of okadaic acid (blue) superimposed with the energetically representative structures obtained from conformational search. Structures of an NMR compatible search (CS I) are in greenish.

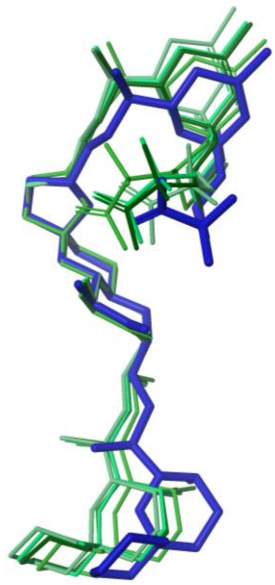

Table S6. CS I, Conformer 1. Gas phase energy and Boltzmann Populations −2695.56653455841 hartrees, 58.61%.

Center Number		Coordinates X	Y	Z
**1**	C1	85.938423	59.391159	20.347820
**2**	C2	86.392860	60.592625	21.191975
**3**	C3	87.774376	60.391079	21.869789
**4**	C4	88.951302	59.979923	20.953747
**5**	C5	89.218025	58.461964	20.917515
**6**	C6	90.453751	58.182812	20.060827
**7**	C7	91.644516	58.968315	20.629108
**8**	C8	91.288322	60.474541	20.686728
**9**	C9	92.408371	61.278221	21.330400
**10**	C10	92.879913	62.416512	20.792999
**11**	C11	92.270668	62.974224	19.518822
**12**	C12	90.923485	62.293095	19.192814
**13**	C13	90.438103	62.522362	17.745958
**14**	C14	89.198853	61.685757	17.483696
**15**	C15	89.166595	60.615349	16.674168
**16**	C16	87.952621	59.741291	16.455696
**17**	C17	87.635223	59.512947	14.983790
**18**	C18	86.835953	58.230499	15.057970
**19**	C19	87.548485	57.466137	16.178650
**20**	C20	88.614578	56.474808	15.654953
**21**	C21	89.231659	55.713634	16.839275
**22**	C22	88.106422	55.037701	17.621374
**23**	C23	87.094421	56.110638	18.057440
**24**	C24	85.944130	55.453075	18.817022
**25**	C25	86.515106	54.658474	19.986126
**26**	C26	87.665611	53.720581	19.612938
**27**	C27	87.195999	52.411205	18.933592
**28**	C28	86.266716	51.555794	19.821550
**29**	C29	85.761269	50.238968	19.177412
**30**	C30	85.045074	49.318207	20.202232
**31**	C31	85.880455	48.889973	21.437641
**32**	C32	85.029259	47.939415	22.292288
**33**	C33	84.542709	46.751320	21.453449
**34**	C34	83.784851	47.266834	20.214048
**35**	C35	83.424500	46.092525	19.277086
**36**	C36	82.577942	46.603386	18.104137
**37**	C37	81.332611	47.300915	18.652786
**38**	C38	81.762192	48.408169	19.624062
**39**	C39	87.248627	48.279404	21.075542
**40**	C40	84.825584	50.531094	17.984020
**41**	C41	86.107628	54.807781	21.261488

Table S6. *Cont.*

Center Number		Coordinates X	Y	Z
**42**	C42	90.153862	64.000816	17.440260
**43**	C43	94.036507	63.196060	21.387562
**44**	C44	85.313530	60.939682	22.232323
**45**	O45	85.827042	58.247368	21.070648
**46**	O46	85.673103	59.492287	19.150784
**47**	O47	86.469421	61.704109	20.322102
**48**	O48	90.128426	60.619358	21.458162
**49**	O49	92.780785	58.759079	19.822449
**50**	O50	91.095932	60.888828	19.362513
**51**	O51	88.194199	58.432640	16.953829
**52**	O52	86.576347	56.766281	16.902910
**53**	O53	88.627876	54.373516	18.769985
**54**	O54	85.077492	56.489895	19.243832
**55**	O55	88.377335	51.676975	18.691595
**56**	O56	84.622169	48.140945	19.508362
**57**	O57	82.596870	47.871960	20.650900
**58**	H58	88.034248	61.336681	22.347324
**59**	H59	87.687187	59.677975	22.690208
**60**	H60	88.756210	60.345993	19.945763
**61**	H61	89.374237	58.083241	21.928223
**62**	H62	88.366226	57.922165	20.513056
**63**	H63	90.671425	57.114635	20.053175
**64**	H64	90.256805	58.471233	19.026707
**65**	H65	91.863335	58.622890	21.640902
**66**	H66	90.155647	62.647152	19.884382
**67**	H67	91.220833	62.180714	17.066999
**68**	H68	87.082794	60.155460	16.967625
**69**	H69	87.081711	60.338558	14.536098
**70**	H70	88.547485	59.355301	14.406977
**71**	H71	85.812523	58.447521	15.368058
**72**	H72	86.809341	57.663166	14.127451
**73**	H73	88.141617	55.781960	14.957982
**74**	H74	89.379814	57.024723	15.106452
**75**	H75	89.947792	54.973236	16.483629
**76**	H76	89.780357	56.401066	17.484564
**77**	H77	87.622574	54.318043	16.961044
**78**	H78	87.583344	56.834087	18.710278
**79**	H79	85.398216	54.775757	18.158175
**80**	H80	88.190903	53.454411	20.532137
**81**	H81	86.705376	52.628151	17.987442
**82**	H82	86.813232	51.341206	20.738115
**83**	H83	85.400314	52.143269	20.126476

Table S6. *Cont.*

Center		Coordinates		
Number		X	Y	Z
**84**	H84	86.620529	49.693756	18.785835
**85**	H85	84.161385	49.851463	20.556786
**86**	H86	86.071068	49.765858	22.057217
**87**	H87	85.594208	47.586575	23.155155
**88**	H88	84.167252	48.477154	22.689888
**89**	H89	85.381599	46.136238	21.127892
**90**	H90	83.879120	46.111652	22.036030
**91**	H91	84.343361	45.627857	18.917908
**92**	H92	82.875328	45.341679	19.846518
**93**	H93	83.160866	47.296932	17.496445
**94**	H94	82.294197	45.775848	17.454006
**95**	H95	80.739388	47.719772	17.839298
**96**	H96	80.696587	46.581852	19.170544
**97**	H97	82.283051	49.204353	19.091175
**98**	H98	80.885696	48.862370	20.086201
**99**	H99	87.756104	47.893665	21.959623
**100**	H100	87.149742	47.461975	20.361343
**101**	H101	87.907806	49.023327	20.627371
**102**	H102	84.462227	49.609028	17.529850
**103**	H103	83.954132	51.108387	18.293219
**104**	H104	85.332031	51.079674	17.191727
**105**	H105	89.785194	64.125122	16.421621
**106**	H106	89.402832	64.408554	18.117861
**107**	H107	91.053459	64.608833	17.534466
**108**	H108	93.720490	64.207504	21.644150
**109**	H109	94.415054	62.719273	22.292328
**110**	H110	94.855545	63.263065	20.671030
**111**	H111	85.559074	61.859779	22.763214
**112**	H112	84.336441	61.079521	21.767925
**113**	H113	85.212395	60.149151	22.977018
**114**	H114	85.528122	57.542999	20.488194
**115**	H115	86.072945	61.400196	19.511171
**116**	H116	92.672752	59.359585	19.094614
**117**	H117	84.934128	57.030109	18.469141
**118**	H118	89.039284	52.311874	18.443222
**119**	H119	92.126320	64.049614	19.625711
**120**	H120	88.305870	61.977985	18.018335
**121**	H121	90.062546	60.321247	16.147848
**122**	H122	86.563820	54.244980	22.062572
**123**	H123	85.310249	55.484222	21.531557
**124**	H124	92.841423	60.870438	22.231379
**125**	H125	92.983070	62.823223	18.707310

Table S7. CS I, Conformer 2. Gas phase energy and Boltzmann Populations −2695.56555785124 hartrees, 20.83%.

Center		Coordinates		
Number		X	Y	Z
**1**	C1	86.176521	59.402092	20.591660
**2**	C2	86.654037	60.332878	21.718271
**3**	C3	88.057289	59.935188	22.238607
**4**	C4	89.169022	59.791603	21.169176
**5**	C5	89.716805	58.358089	21.046959
**6**	C6	90.876930	58.337139	20.048229
**7**	C7	91.942162	59.354698	20.487354
**8**	C8	91.294914	60.756691	20.611015
**9**	C9	92.288124	61.788704	21.121283
**10**	C10	92.406883	63.008549	20.568670
**11**	C11	91.515251	63.419041	19.409948
**12**	C12	90.342278	62.432777	19.210299
**13**	C13	89.672829	62.554909	17.824179
**14**	C14	88.737564	61.383816	17.581375
**15**	C15	88.934509	60.448284	16.638229
**16**	C16	88.046036	59.244446	16.419600
**17**	C17	87.475525	59.165829	15.003479
**18**	C18	87.540100	57.685749	14.646619
**19**	C19	88.127106	57.031250	15.899818
**20**	C20	89.126129	55.898071	15.576921
**21**	C21	89.612053	55.254974	16.883104
**22**	C22	88.385994	54.767204	17.647429
**23**	C23	87.455498	55.961273	17.912962
**24**	C24	86.198982	55.475433	18.639511
**25**	C25	86.586906	54.684200	19.886814
**26**	C26	87.688515	53.643913	19.673159
**27**	C27	87.176895	52.332344	19.030041
**28**	C28	86.133667	51.581089	19.882542
**29**	C29	85.544266	50.317333	19.206173
**30**	C30	84.808853	49.382771	20.202698
**31**	C31	85.642288	48.875317	21.409264
**32**	C32	84.773598	47.906883	22.224667
**33**	C33	84.252586	46.772915	21.334391
**34**	C34	83.497543	47.364498	20.127445
**35**	C35	83.106148	46.247078	19.136433
**36**	C36	82.266426	46.834286	17.994564
**37**	C37	81.041130	47.530388	18.584084
**38**	C38	81.499741	48.577187	19.606064
**39**	C39	86.992447	48.250843	21.007404
**40**	C40	84.596184	50.707718	18.051495
**41**	C41	86.055573	54.901630	21.104803

Table S7. *Cont.*

**Center**		**Coordinates**		
**Number**		**X**	**Y**	**Z**
**42**	C42	88.919151	63.882767	17.650194
**43**	C43	93.424904	64.032661	21.031172
**44**	C44	85.624855	60.376694	22.860867
**45**	O45	86.012253	58.125778	21.015785
**46**	O46	86.008438	59.791103	19.436468
**47**	O47	86.707344	61.642368	21.194597
**48**	O48	90.240913	60.665222	21.527151
**49**	O49	92.996574	59.374432	19.553127
**50**	O50	90.853706	61.107487	19.328154
**51**	O51	88.799988	58.051937	16.555159
**52**	O52	87.063316	56.529564	16.664160
**53**	O53	88.764809	54.167431	18.881681
**54**	O54	85.421043	56.621155	18.947660
**55**	O55	88.320290	51.512463	18.910337
**56**	O56	84.347633	48.252262	19.458263
**57**	O57	82.327080	47.973438	20.600729
**58**	H58	88.367867	60.673332	22.977434
**59**	H59	87.973129	59.004276	22.796984
**60**	H60	88.785110	60.107941	20.200432
**61**	H61	90.061684	58.004940	22.018551
**62**	H62	88.931564	57.674988	20.723589
**63**	H63	91.307632	57.337711	19.994013
**64**	H64	90.507614	58.571362	19.048386
**65**	H65	92.343018	59.069454	21.460808
**66**	H66	89.588829	62.591187	19.984776
**67**	H67	90.455025	62.508148	17.064795
**68**	H68	87.234390	59.227993	17.147907
**69**	H69	86.458344	59.558147	14.963836
**70**	H70	88.080139	59.738373	14.298585
**71**	H71	86.569183	57.251461	14.401868
**72**	H72	88.212814	57.534073	13.800322
**73**	H73	88.631050	55.161541	14.943235
**74**	H74	89.963936	56.308846	15.013496
**75**	H75	90.285439	54.425060	16.670189
**76**	H76	90.172195	55.977501	17.478603
**77**	H77	87.878372	54.038265	17.016949
**78**	H78	87.969101	56.705944	18.524910
**79**	H79	85.624908	54.819405	17.983110
**80**	H80	88.112350	53.399082	20.648933
**81**	H81	86.767029	52.524132	18.041546
**82**	H82	86.614838	51.327477	20.824850
**83**	H83	85.311028	52.245743	20.145645

Table S7. *Cont.*

**Center**		**Coordinates**		
**Number**		**X**	**Y**	**Z**
**84**	H84	86.364563	49.746590	18.770493
**85**	H85	83.945229	49.926666	20.588652
**86**	H86	85.858994	49.714855	22.068953
**87**	H87	85.335442	47.499466	23.064981
**88**	H88	83.928452	48.446484	22.654202
**89**	H89	85.073822	46.155514	20.972328
**90**	H90	83.579849	46.120838	21.891195
**91**	H91	84.012039	45.781460	18.749327
**92**	H92	82.543915	45.481819	19.671486
**93**	H93	82.862305	47.542709	17.418972
**94**	H94	81.960632	46.048729	17.305140
**95**	H95	80.453987	48.001186	17.794436
**96**	H96	80.392502	46.801487	19.069780
**97**	H97	82.033035	49.388031	19.110216
**98**	H98	80.635628	49.024185	20.096310
**99**	H99	87.497498	47.811222	21.866899
**100**	H100	86.868813	47.472023	20.255796
**101**	H101	87.665634	48.999523	20.589428
**102**	H102	84.192444	49.825207	17.555367
**103**	H103	83.750778	51.292919	18.412580
**104**	H104	85.101425	51.286404	17.280781
**105**	H105	88.447304	63.937717	16.668718
**106**	H106	88.137360	63.996819	18.402878
**107**	H107	89.590225	64.737846	17.736046
**108**	H108	92.922508	64.939529	21.370613
**109**	H109	94.029770	63.650593	21.854925
**110**	H110	94.094398	64.296288	20.211250
**111**	H111	85.909378	61.103672	23.621048
**112**	H112	84.637093	60.654774	22.494133
**113**	H113	85.532310	59.409348	23.354132
**114**	H114	85.748177	57.560219	20.283035
**115**	H115	86.641968	61.524078	20.235332
**116**	H116	92.680672	59.919842	18.842766
**117**	H117	85.315926	57.085308	18.121000
**118**	H118	89.040977	52.091984	18.693117
**119**	H119	91.130043	64.423965	19.585329
**120**	H120	87.879662	61.310665	18.234602
**121**	H121	89.793289	60.522667	15.984978
**122**	H122	86.378128	54.333652	21.964808
**123**	H123	85.281395	55.636238	21.268661
**124**	H124	92.924583	61.486752	21.939465
**125**	H125	92.133583	63.463707	18.512917

Table S8. CS I, Conformer 3. Gas phase energy and Boltzmann Populations −2695.56509557079 hartrees, 12.77%.

**Center**		**Coordinates**		
**Number**		**X**	**Y**	**Z**
**1**	C1	86.193192	59.313828	20.594460
**2**	C2	86.677666	60.290848	21.679338
**3**	C3	88.115227	59.989704	22.178545
**4**	C4	89.206650	59.852951	21.089464
**5**	C5	89.725784	58.411724	20.920134
**6**	C6	90.873489	58.393982	19.907120
**7**	C7	91.964630	59.379581	20.357172
**8**	C8	91.344696	60.788845	20.522987
**9**	C9	92.365280	61.796055	21.030281
**10**	C10	92.503586	63.017311	20.484138
**11**	C11	91.603531	63.457951	19.341259
**12**	C12	90.399666	62.506039	19.169872
**13**	C13	89.674751	62.641331	17.812672
**14**	C14	88.717758	61.476269	17.637423
**15**	C15	88.862137	60.507092	16.719923
**16**	C16	87.981369	59.282410	16.630825
**17**	C17	87.232109	59.147091	15.299958
**18**	C18	87.653061	57.784580	14.773096
**19**	C19	88.187294	57.090630	16.021074
**20**	C20	89.241280	56.005585	15.705320
**21**	C21	89.681610	55.319931	17.006390
**22**	C22	88.437798	54.772034	17.701015
**23**	C23	87.469330	55.937302	17.963573
**24**	C24	86.205673	55.416782	18.651148
**25**	C25	86.582031	54.612167	19.892883
**26**	C26	87.702316	53.591091	19.684086
**27**	C27	87.223160	52.281387	19.011099
**28**	C28	86.161659	51.518600	19.832872
**29**	C29	85.646126	50.203579	19.192905
**30**	C30	84.779060	49.370075	20.174946
**31**	C31	85.461098	48.953217	21.504799
**32**	C32	84.475082	48.089592	22.305033
**33**	C33	84.005791	46.893032	21.468365
**34**	C34	83.406403	47.392445	20.138876
**35**	C35	83.073326	46.198292	19.216986
**36**	C36	82.378960	46.700756	17.944672
**37**	C37	81.127937	47.488407	18.335697
**38**	C38	81.522415	48.613503	19.301355
**39**	C39	86.820442	48.253738	21.308050
**40**	C40	84.852257	50.492138	17.900043
**41**	C41	86.028358	54.807983	21.105421
**42**	C42	88.930237	63.976742	17.671322

Table S8. *Cont.*

**Center**		**Coordinates**		
**Number**		**X**	**Y**	**Z**
**43**	C43	93.553894	64.012131	20.938194
**44**	C44	85.678795	60.307644	22.849529
**45**	O45	86.242950	58.019039	20.995081
**46**	O46	85.773232	59.696842	19.503740
**47**	O47	86.650665	61.589241	21.120775
**48**	O48	90.304848	60.693413	21.455662
**49**	O49	93.004105	59.403873	19.406134
**50**	O50	90.888977	61.170666	19.255840
**51**	O51	88.781204	58.112309	16.743433
**52**	O52	87.106377	56.523113	16.712812
**53**	O53	88.789948	54.145939	18.930407
**54**	O54	85.407318	56.544857	18.971544
**55**	O55	88.376183	51.471939	18.918198
**56**	O56	84.359276	48.187428	19.488373
**57**	O57	82.218155	48.079937	20.428225
**58**	H58	88.402924	60.789478	22.862091
**59**	H59	88.103027	59.090233	22.795233
**60**	H60	88.807198	60.204964	20.139854
**61**	H61	90.075668	58.024513	21.877815
**62**	H62	88.925591	57.751034	20.587532
**63**	H63	91.283295	57.387375	19.823704
**64**	H64	90.495598	58.660404	18.918444
**65**	H65	92.374367	59.063911	21.318073
**66**	H66	89.679108	62.673779	19.973572
**67**	H67	90.419823	62.579105	17.018166
**68**	H68	87.263077	59.279617	17.449762
**69**	H69	86.156418	59.175606	15.476455
**70**	H70	87.473831	59.940945	14.592482
**71**	H71	86.848068	57.213768	14.309361
**72**	H72	88.466713	57.900425	14.056028
**73**	H73	88.804909	55.282040	15.015588
**74**	H74	90.093071	56.467068	15.204517
**75**	H75	90.385803	54.516254	16.792219
**76**	H76	90.196617	56.031452	17.653723
**77**	H77	87.973213	54.046974	17.033031
**78**	H78	87.948280	56.678993	18.606503
**79**	H79	85.654747	54.765083	17.970818
**80**	H80	88.108101	53.336361	20.665026
**81**	H81	86.840126	52.483086	18.013355
**82**	H82	86.600822	51.315826	20.807997
**83**	H83	85.305199	52.165379	20.024647
**84**	H84	86.506432	49.595215	18.912373

Table S8. *Cont.*

**Center**		**Coordinates**		
**Number**		**X**	**Y**	**Z**
**85**	H85	83.897156	49.965778	20.416489
**86**	H86	85.640060	49.844440	22.105709
**87**	H87	84.929863	47.745853	23.234119
**88**	H88	83.610710	48.690575	22.591366
**89**	H89	84.835518	46.219372	21.254414
**90**	H90	83.251312	46.317112	22.005022
**91**	H91	83.994728	45.668983	18.971909
**92**	H92	82.427406	45.504574	19.756559
**93**	H93	83.058846	47.333782	17.372761
**94**	H94	82.113663	45.861282	17.302074
**95**	H95	80.643990	47.901566	17.450148
**96**	H96	80.402802	46.828720	18.813841
**97**	H97	82.139137	49.355644	18.793394
**98**	H98	80.632187	49.134644	19.653528
**99**	H99	87.214211	47.881344	22.253670
**100**	H100	86.745377	47.411465	20.620285
**101**	H101	87.563217	48.939861	20.900248
**102**	H102	84.482498	49.571644	17.447823
**103**	H103	83.989647	51.129097	18.096115
**104**	H104	85.466133	50.976185	17.142420
**105**	H105	88.415627	64.037910	16.711987
**106**	H106	88.184586	64.099838	18.457743
**107**	H107	89.615944	64.822105	17.727459
**108**	H108	93.080948	64.930687	21.286551
**109**	H109	94.156761	63.610783	21.753481
**110**	H110	94.221466	64.260689	20.112770
**111**	H111	85.943588	61.069992	23.582674
**112**	H112	84.663391	60.516777	22.510208
**113**	H113	85.659714	59.347927	23.367615
**114**	H114	85.908279	57.451527	20.293346
**115**	H115	86.079636	61.520462	20.362936
**116**	H116	92.685448	59.972836	18.715590
**117**	H117	85.273827	57.009819	18.150080
**118**	H118	89.097969	52.057667	18.720984
**119**	H119	91.250053	64.473122	19.523605
**120**	H120	87.897003	61.427494	18.338907
**121**	H121	89.674149	60.558071	16.009808
**122**	H122	86.345154	54.232613	21.962898
**123**	H123	85.244370	55.532784	21.267246
**124**	H124	93.006935	61.472569	21.835981
**125**	H125	92.203033	63.484474	18.430937

Table S9. CS I, Conformer 4. Gas phase energy and Boltzmann Populations −2695.56407862476 hartrees, 4.35%.

**Center**		**Coordinates**		
**Number**		**X**	**Y**	**Z**
**1**	C1	85.736389	58.924442	20.079184
**2**	C2	86.005821	60.136490	20.985167
**3**	C3	87.334167	60.036224	21.781301
**4**	C4	88.619820	59.755844	20.967699
**5**	C5	89.024467	58.268673	20.925571
**6**	C6	90.353790	58.122803	20.183258
**7**	C7	91.411751	58.994877	20.874958
**8**	C8	90.917122	60.461575	20.925570
**9**	C9	91.897285	61.344852	21.683113
**10**	C10	92.310181	62.533699	21.210798
**11**	C11	91.769890	63.067242	19.895790
**12**	C12	90.524582	62.277924	19.436039
**13**	C13	90.152199	62.499485	17.954903
**14**	C14	89.020714	61.564919	17.566380
**15**	C15	89.155937	60.516624	16.738710
**16**	C16	88.048195	59.547192	16.393957
**17**	C17	87.882431	59.335197	14.895021
**18**	C18	87.194008	57.988220	14.869953
**19**	C19	87.868416	57.255024	16.034281
**20**	C20	89.056175	56.369938	15.587824
**21**	C21	89.629509	55.629337	16.806650
**22**	C22	88.501556	54.841164	17.470793
**23**	C23	87.368332	55.814472	17.836929
**24**	C24	86.215721	55.043110	18.476580
**25**	C25	86.747757	54.266235	19.675377
**26**	C26	88.001007	53.436417	19.386778
**27**	C27	87.705299	52.112339	18.641260
**28**	C28	86.763000	51.167702	19.416246
**29**	C29	86.443573	49.823467	18.716503
**30**	C30	85.694611	48.878029	19.694056
**31**	C31	85.963982	47.360645	19.530203
**32**	C32	85.149452	46.624458	20.604340
**33**	C33	83.665894	47.009274	20.507793
**34**	C34	83.509201	48.540283	20.630518
**35**	C35	82.039474	48.951706	20.391853
**36**	C36	81.877953	50.459908	20.621771
**37**	C37	82.347183	50.803783	22.036005
**38**	C38	83.785995	50.305588	22.225376
**39**	C39	85.699089	46.791645	18.124971
**40**	C40	85.669151	50.059242	17.398684
**41**	C41	86.220085	54.346458	20.912300
**42**	C42	89.765930	63.954628	17.649118

Table S9. *Cont.*

**Center**		**Coordinates**		
**Number**		**X**	**Y**	**Z**
**43**	C43	93.333328	63.397400	21.922234
**44**	C44	84.811981	60.359135	21.930134
**45**	O45	85.665169	57.755207	20.765469
**46**	O46	85.567871	59.036186	18.865671
**47**	O47	86.060005	61.274593	20.148643
**48**	O48	89.684425	60.482895	21.589760
**49**	O49	92.632225	58.908085	20.175961
**50**	O50	90.807678	60.890701	19.596647
**51**	O51	88.359962	58.250031	16.883226
**52**	O52	86.899559	56.457558	16.654572
**53**	O53	88.975060	54.189289	18.647055
**54**	O54	85.230438	55.992565	18.845387
**55**	O55	88.958755	51.477203	18.502117
**56**	O56	84.294243	49.158783	19.647867
**57**	O57	83.870003	48.910404	21.934561
**58**	H58	87.465057	60.986580	22.300371
**59**	H59	87.238472	59.294750	22.575205
**60**	H60	88.482742	60.131229	19.953615
**61**	H61	89.120796	57.876297	21.938557
**62**	H62	88.265144	57.668266	20.431864
**63**	H63	90.666870	57.078606	20.176346
**64**	H64	90.227074	58.420078	19.140676
**65**	H65	91.566528	58.643852	21.896608
**66**	H66	89.668915	62.544960	20.060314
**67**	H67	91.019897	62.244255	17.344719
**68**	H68	87.103569	59.869717	16.834263
**69**	H69	87.300858	60.123558	14.416769
**70**	H70	88.852127	59.271847	14.399577
**71**	H71	86.132248	58.109066	15.090666
**72**	H72	87.298897	57.447514	13.929172
**73**	H73	88.706223	55.660206	14.837280
**74**	H74	89.817108	56.996201	15.121320
**75**	H75	90.434189	54.960899	16.501341
**76**	H76	90.059341	56.341370	17.512611
**77**	H77	88.139671	54.103531	16.754608
**78**	H78	87.735428	56.557037	18.545904
**79**	H79	85.788795	54.341396	17.758055
**80**	H80	88.463890	53.188435	20.343821
**81**	H81	87.290276	52.313423	17.655914
**82**	H82	87.212563	50.975227	20.391260
**83**	H83	85.819885	51.675034	19.622158
**84**	H84	87.395615	49.348709	18.475216

Table S9. *Cont.*

**Center Number**		**Coordinates X**	**Y**	**Z**
**85**	H85	86.042763	49.120384	20.699209
**86**	H86	87.019455	47.185390	19.744225
**87**	H87	85.267944	45.545261	20.507193
**88**	H88	85.526039	46.884098	21.594828
**89**	H89	83.239861	46.682751	19.559284
**90**	H90	83.091698	46.530064	21.301237
**91**	H91	81.754570	48.682533	19.374254
**92**	H92	81.399780	48.389942	21.073633
**93**	H93	82.462807	51.014820	19.886648
**94**	H94	80.837486	50.754189	20.484751
**95**	H95	82.293549	51.879677	22.205463
**96**	H96	81.694107	50.334435	22.772783
**97**	H97	84.473129	50.865700	21.590164
**98**	H98	84.107094	50.469376	23.254000
**99**	H99	85.806732	45.707008	18.113888
**100**	H100	84.695053	47.029034	17.773903
**101**	H101	86.411697	47.186192	17.401035
**102**	H102	85.332657	49.133675	16.939611
**103**	H103	84.780350	50.667698	17.568239
**104**	H104	86.284744	50.565590	16.656610
**105**	H105	89.480225	64.071342	16.603292
**106**	H106	88.923988	64.278557	18.262041
**107**	H107	90.595764	64.636261	17.835073
**108**	H108	92.905251	64.370064	22.165987
**109**	H109	93.670654	62.933327	22.849646
**110**	H110	94.204018	63.554806	21.285141
**111**	H111	84.926842	61.281586	22.499882
**112**	H112	83.871338	60.426994	21.381910
**113**	H113	84.715820	59.541977	22.646204
**114**	H114	85.481087	57.044582	20.144176
**115**	H115	85.762314	60.961330	19.299889
**116**	H116	92.538086	59.514759	19.451338
**117**	H117	85.107529	56.538227	18.071051
**118**	H118	89.588516	52.167210	18.328146
**119**	H119	91.519775	64.122589	20.007360
**120**	H120	88.061066	61.764931	18.022152
**121**	H121	90.117928	60.314552	16.291544
**122**	H122	86.650581	53.800629	21.738916
**123**	H123	85.348114	54.947380	21.123960
**124**	H124	92.282776	60.954334	22.612898
**125**	H125	92.563004	63.000477	19.150545

Table S10. CS I, Conformer 5. Gas phase energy and Boltzmann Populations −2695.56290872663 hartrees, 1.26%.

Center		Coordinates		
**Number**		**X**	**Y**	**Z**
**1**	C1	86.125305	59.076077	20.963697
**2**	C2	86.633011	60.396461	21.564951
**3**	C3	88.127510	60.357292	21.981575
**4**	C4	89.142906	59.901501	20.907084
**5**	C5	89.515221	58.407688	20.988312
**6**	C6	90.588905	58.093571	19.945488
**7**	C7	91.799675	59.007751	20.182634
**8**	C8	91.344475	60.487606	20.140989
**9**	C9	92.498444	61.425209	20.463409
**10**	C10	92.772217	62.512985	19.722698
**11**	C11	91.895912	62.874905	18.536579
**12**	C12	90.568863	62.085117	18.554083
**13**	C13	89.807518	62.107216	17.211895
**14**	C14	88.610672	61.176487	17.287382
**15**	C15	88.512848	60.015068	16.621279
**16**	C16	87.351898	59.053921	16.738791
**17**	C17	86.785553	58.629749	15.390203
**18**	C18	86.116386	57.323524	15.757735
**19**	C19	87.081650	56.743206	16.796709
**20**	C20	88.104828	55.754269	16.189537
**21**	C21	88.988190	55.179977	17.308779
**22**	C22	88.083290	54.545784	18.364008
**23**	C23	87.090851	55.607643	18.866297
**24**	C24	86.154976	54.986252	19.900324
**25**	C25	86.990257	54.369583	21.017431
**26**	C26	88.121368	53.457752	20.534588
**27**	C27	87.637283	52.053501	20.097580
**28**	C28	87.014175	51.225845	21.248558
**29**	C29	86.245598	49.935913	20.853706
**30**	C30	85.068100	50.172005	19.873930
**31**	C31	84.027733	51.231121	20.317028
**32**	C32	82.943390	51.309124	19.232655
**33**	C33	82.323868	49.924797	18.994621
**34**	C34	83.431488	48.904827	18.656717
**35**	C35	82.845497	47.477165	18.587225
**36**	C36	83.932426	46.490021	18.142735
**37**	C37	84.497734	46.942257	16.795809
**38**	C38	84.998741	48.387039	16.919781
**39**	C39	83.429848	50.966461	21.713821
**40**	C40	87.188591	48.832829	20.338335
**41**	C41	86.815529	54.651608	22.323086
**42**	C42	89.356529	63.518623	16.806204

Table S10. *Cont.*

**Center**		**Coordinates**		
**Number**		**X**	**Y**	**Z**
**43**	C43	93.954727	63.422695	19.992884
**44**	C44	85.741066	60.809959	22.748684
**45**	O45	86.238441	58.026585	21.817499
**46**	O46	85.636810	59.012424	19.836365
**47**	O47	86.460701	61.393799	20.578241
**48**	O48	90.339859	60.662128	21.101030
**49**	O49	92.779541	58.763584	19.199877
**50**	O50	90.878563	60.724186	18.841312
**51**	O51	87.783882	57.831562	17.320372
**52**	O52	86.318130	56.086269	17.768702
**53**	O53	88.857994	54.058651	19.457132
**54**	O54	85.306511	56.018822	20.370743
**55**	O55	88.804855	51.403801	19.644478
**56**	O56	84.393234	48.927925	19.677135
**57**	O57	83.954453	49.235916	17.397572
**58**	H58	88.396812	61.367764	22.291704
**59**	H59	88.249519	59.747753	22.877552
**60**	H60	88.736916	60.129040	19.921444
**61**	H61	89.884735	58.165813	21.985477
**62**	H62	88.647766	57.775955	20.818085
**63**	H63	90.883713	57.046299	20.014265
**64**	H64	90.182152	58.240818	18.943356
**65**	H65	92.228333	58.802395	21.164986
**66**	H66	89.917213	62.478531	19.337509
**67**	H67	90.475372	61.729252	16.436329
**68**	H68	86.562492	59.477852	17.361189
**69**	H69	86.095901	59.362228	14.970302
**70**	H70	87.584373	58.453674	14.668621
**71**	H71	85.154320	57.519566	16.233837
**72**	H72	85.962196	56.645824	14.917856
**73**	H73	87.565224	54.955925	15.678640
**74**	H74	88.709976	56.273071	15.445549
**75**	H75	89.680504	54.441380	16.905386
**76**	H76	89.592773	55.970749	17.755159
**77**	H77	87.542130	53.724304	17.894243
**78**	H78	87.635178	56.431427	19.328619
**79**	H79	85.549065	54.208645	19.433205
**80**	H80	88.826546	53.330395	21.358124
**81**	H81	86.931221	52.140720	19.274914
**82**	H82	87.790924	50.976299	21.972563
**83**	H83	86.322754	51.859234	21.801645
**84**	H84	85.819260	49.542385	21.776745

Table S10. *Cont.*

**Center**		**Coordinates**		
**Number**		**X**	**Y**	**Z**
**85**	H85	85.491333	50.479759	18.917425
**86**	H86	84.511726	52.206650	20.353657
**87**	H87	82.170631	52.026005	19.510662
**88**	H88	83.379639	51.674149	18.301653
**89**	H89	81.783676	49.584076	19.877810
**90**	H90	81.607986	49.957088	18.172749
**91**	H91	82.452454	47.204594	19.567253
**92**	H92	82.012161	47.470806	17.883585
**93**	H93	84.727257	46.447868	18.888933
**94**	H94	83.520340	45.484100	18.063162
**95**	H95	85.309906	46.285542	16.482332
**96**	H96	83.727539	46.882950	16.025785
**97**	H97	85.861031	48.437397	17.585505
**98**	H98	85.331764	48.753120	15.948592
**99**	H99	82.622955	51.665169	21.935280
**100**	H100	83.029633	49.956074	21.798170
**101**	H101	84.176308	51.083561	22.498510
**102**	H102	86.665146	47.881695	20.238224
**103**	H103	87.599167	49.081463	19.359997
**104**	H104	88.024757	48.675816	21.019623
**105**	H105	88.797157	63.496002	15.870474
**106**	H106	88.714195	63.963650	17.567003
**107**	H107	90.207520	64.183151	16.657660
**108**	H108	93.613976	64.438927	20.192661
**109**	H109	94.530441	63.082569	20.854334
**110**	H110	94.618958	63.446396	19.128603
**111**	H111	86.008163	61.801064	23.116228
**112**	H112	84.687042	60.836777	22.468918
**113**	H113	85.841301	60.113762	23.582346
**114**	H114	85.892921	57.240147	21.385508
**115**	H115	85.943138	60.971375	19.899422
**116**	H116	92.492714	59.263462	18.444958
**117**	H117	84.986076	56.456894	19.584490
**118**	H118	89.342430	52.077366	19.242847
**119**	H119	91.690559	63.945595	18.550003
**120**	H120	87.812309	61.481216	17.949520
**121**	H121	89.315773	59.709774	15.966801
**122**	H122	87.453522	54.216484	23.078299
**123**	H123	86.032967	55.312267	22.665876
**124**	H124	93.121315	61.156155	21.303188
**125**	H125	92.455223	62.664902	17.624464

S5. Representative NMR Incompatible Conformational Search (CS II)

Figure S19. Crystallographic structure of okadaic acid (blue) superimposed with the energetically representative structures obtained from conformational search. Structures of an NMR incompatible search (CS II) are in reddish.

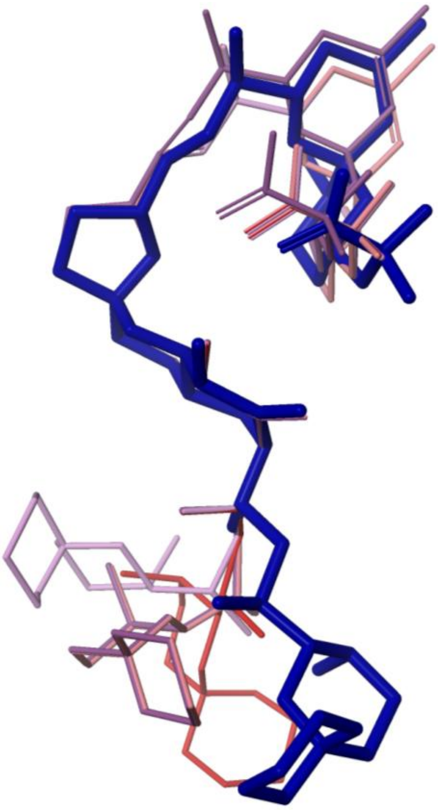

Table S11. CS II, Conformer 1. Gas phase energy and Boltzmann Populations −2695.556258 hartrees, 49.92%.

Center		Coordinates		
Number		X	Y	Z
**1**	C1	76.108307	57.051773	13.750768
**2**	C2	75.429855	58.363914	14.170176
**3**	C3	74.437378	58.179733	15.341824
**4**	C4	75.022827	57.547798	16.623804
**5**	C5	73.951668	57.380959	17.714109
**6**	C6	74.608322	56.832005	18.978533
**7**	C7	75.720596	57.795208	19.409668
**8**	C8	76.744438	57.968029	18.257376
**9**	C9	77.787117	59.010006	18.638943
**10**	C10	79.108200	58.785065	18.546137
**11**	C11	79.621376	57.467831	17.999399
**12**	C12	78.510857	56.713223	17.239944
**13**	C13	78.848022	55.240070	16.924829
**14**	C14	77.715454	54.636383	16.120249
**15**	C15	76.835739	53.731888	16.575811

Table S11. *Cont.*

Center		Coordinates		
Number		X	Y	Z
**16**	C16	75.674141	53.221558	15.755466
**17**	C17	75.568810	51.708580	15.674277
**18**	C18	74.126579	51.540218	15.226770
**19**	C19	73.432053	52.788177	15.803410
**20**	C20	72.401237	52.447525	16.900993
**21**	C21	71.726028	53.737736	17.382313
**22**	C22	71.109398	54.437626	16.173166
**23**	C23	72.208061	54.707359	15.126352
**24**	C24	71.608162	55.397099	13.892622
**25**	C25	70.787636	56.615200	14.323368
**26**	C26	69.877159	56.393219	15.532850
**27**	C27	68.469101	55.832832	15.190156
**28**	C28	67.655487	55.469902	16.451273
**29**	C29	66.107574	55.435360	16.307262
**30**	C30	65.463570	54.317921	15.434609
**31**	C31	65.423157	52.866890	15.986176
**32**	C32	64.641068	52.013367	14.976174
**33**	C33	65.275459	52.119820	13.582923
**34**	C34	65.324104	53.596909	13.141768
**35**	C35	66.097092	53.736790	11.811905
**36**	C36	66.053688	55.194866	11.336917
**37**	C37	64.594818	55.633678	11.202279
**38**	C38	63.870186	55.399796	12.534517
**39**	C39	66.792381	52.240955	16.307014
**40**	C40	65.442276	55.495792	17.694809
**41**	C41	70.905571	57.829014	13.749936
**42**	C42	80.170998	55.071873	16.160881
**43**	C43	80.153801	59.789085	18.990238
**44**	C44	74.743706	58.992775	12.948071
**45**	O45	77.408821	57.321247	13.480883
**46**	O46	75.555595	55.959755	13.657129
**47**	O47	76.444305	59.277065	14.575750
**48**	O48	76.064102	58.406437	17.110668
**49**	O49	76.354858	57.300713	20.566484
**50**	O50	77.340363	56.715534	18.055521
**51**	O51	74.445923	53.558487	16.369848
**52**	O52	72.800461	53.462475	14.749808
**53**	O53	70.524529	55.666550	16.581560
**54**	O54	72.676163	55.777962	13.038436
**55**	O55	68.546272	54.718143	14.328876
**56**	O56	66.008812	54.339535	14.115639
**57**	O57	64.008926	54.038456	12.943127

Table S11. *Cont.*

Center		Coordinates		
Number		X	Y	Z
**58**	H58	73.990303	59.143097	15.589536
**59**	H59	73.610176	57.559288	14.996874
**60**	H60	75.435562	56.564980	16.391291
**61**	H61	73.473900	58.338184	17.926580
**62**	H62	73.162125	56.707710	17.382320
**63**	H63	73.866989	56.729080	19.771109
**64**	H64	75.008026	55.834545	18.786991
**65**	H65	75.288483	58.770378	19.641142
**66**	H66	78.297371	57.236324	16.305279
**67**	H67	78.928200	54.695034	17.866661
**68**	H68	75.702156	53.644321	14.750332
**69**	H69	76.286629	51.267860	14.982249
**70**	H70	75.713646	51.261459	16.658510
**71**	H71	74.055977	51.558052	14.138186
**72**	H72	73.672691	50.617878	15.589911
**73**	H73	71.662689	51.758118	16.489866
**74**	H74	72.907616	51.941956	17.723717
**75**	H75	70.957512	53.512146	18.121140
**76**	H76	72.454491	54.387505	17.868404
**77**	H77	70.349640	53.776974	15.757547
**78**	H78	72.969894	55.357929	15.555001
**79**	H79	70.959061	54.698822	13.363420
**80**	H80	69.700310	57.376362	15.971578
**81**	H81	67.937859	56.611401	14.640248
**82**	H82	68.049843	54.561047	16.896490
**83**	H83	67.879501	56.241833	17.188786
**84**	H84	65.839226	56.371746	15.815718
**85**	H85	64.420395	54.624687	15.337218
**86**	H86	64.847107	52.863308	16.912289
**87**	H87	64.604279	50.971855	15.295983
**88**	H88	63.604900	52.352070	14.929430
**89**	H89	66.287567	51.714409	13.585257
**90**	H90	64.700279	51.550301	12.852210
**91**	H91	67.126892	53.407822	11.957623
**92**	H92	65.643860	53.079201	11.068956
**93**	H93	66.577614	55.837204	12.046294
**94**	H94	66.569778	55.295952	10.382098
**95**	H95	64.536346	56.684944	10.918287
**96**	H96	64.104012	55.065323	10.411174
**97**	H97	64.259468	56.066422	13.305314
**98**	H98	62.809525	55.628281	12.429527
**99**	H99	66.712044	51.161369	16.434416

Table S11. *Cont.*

Center		Coordinates		
Number		X	Y	Z
**100**	H100	67.522858	52.427517	15.519726
**101**	H101	67.191856	52.626747	17.243059
**102**	H102	64.355110	55.522594	17.613152
**103**	H103	65.709618	54.632000	18.304033
**104**	H104	65.745995	56.388577	18.242207
**105**	H105	80.342560	54.025936	15.904432
**106**	H106	80.169060	55.644821	15.232762
**107**	H107	81.023544	55.399296	16.755669
**108**	H108	80.796921	60.063904	18.153765
**109**	H109	79.694122	60.698994	19.377598
**110**	H110	80.776993	59.362881	19.776924
**111**	H111	74.293610	59.954639	13.194332
**112**	H112	75.445724	59.157600	12.129434
**113**	H113	73.950623	58.345753	12.569774
**114**	H114	77.420509	58.253006	13.715962
**115**	H115	76.519165	59.175243	15.526061
**116**	H116	76.972359	56.652126	20.250748
**117**	H117	73.430481	55.246925	13.273880
**118**	H118	67.640495	54.464680	14.146080
**119**	H119	80.463219	57.658867	17.333239
**120**	H120	77.616226	54.998119	15.107305
**121**	H121	76.919792	53.354340	17.584005
**122**	H122	70.335190	58.675194	14.101346
**123**	H123	71.566696	57.992874	12.912091
**124**	H124	77.415474	59.941650	19.039204
**125**	H125	79.995270	56.870209	18.831408

Table S12. CS II, Conformer 2. Gas phase energy and Boltzmann Populations −2695.555664 hartrees, 26.61%.

Center		Coordinates		
Number		X	Y	Z
**1**	C1	76.679565	57.083557	14.014741
**2**	C2	75.993996	58.427334	14.294632
**3**	C3	74.684105	58.262936	15.102359
**4**	C4	74.834404	57.612045	16.494566
**5**	C5	73.474121	57.443493	17.190573
**6**	C6	73.688347	56.927303	18.613255
**7**	C7	74.609932	57.900974	19.359800
**8**	C8	75.948029	58.040981	18.591066
**9**	C9	76.844437	59.074860	19.254337
**10**	C10	78.140831	58.844788	19.519381
**11**	C11	78.782600	57.528740	19.123833
**12**	C12	77.899879	56.739285	18.131910

Table S12. *Cont.*

**Center**		**Coordinates**		
**Number**		**X**	**Y**	**Z**
**13**	C13	78.311714	55.257053	17.981783
**14**	C14	77.275505	54.519310	17.143671
**15**	C15	77.476547	53.832798	16.003962
**16**	C16	76.367012	53.180557	15.204565
**17**	C17	76.429428	51.661335	15.155599
**18**	C18	74.991142	51.276173	14.826412
**19**	C19	74.192970	52.554558	15.138440
**20**	C20	72.975540	52.311642	16.055592
**21**	C21	72.225716	53.634445	16.265236
**22**	C22	71.853302	54.209091	14.898273
**23**	C23	73.132736	54.383587	14.058359
**24**	C24	72.784248	54.941875	12.670822
**25**	C25	71.925232	56.198635	12.821101
**26**	C26	70.797478	56.095783	13.850586
**27**	C27	69.466194	55.518291	13.295045
**28**	C28	68.404976	55.318970	14.396938
**29**	C29	67.002632	54.865612	13.914270
**30**	C30	66.052963	54.542171	15.098755
**31**	C31	66.546646	53.460644	16.095234
**32**	C32	65.451645	53.240894	17.149340
**33**	C33	64.122353	52.876366	16.476387
**34**	C34	63.743954	53.963020	15.450235
**35**	C35	62.494102	53.534622	14.649531
**36**	C36	62.063313	54.668659	13.710460
**37**	C37	61.809456	55.931202	14.535032
**38**	C38	63.065041	56.259560	15.353296
**39**	C39	66.972420	52.143272	15.416993
**40**	C40	66.358788	55.925064	12.993842
**41**	C41	72.174927	57.350311	12.167266
**42**	C42	79.746208	55.100342	17.440271
**43**	C43	79.029541	59.843033	20.235088
**44**	C44	75.739639	59.150398	12.963075
**45**	O45	78.012611	57.240734	14.198514
**46**	O46	76.105362	56.065853	13.639646
**47**	O47	76.897598	59.246719	15.028807
**48**	O48	75.669083	58.467503	17.284851
**49**	O49	74.836266	57.434593	20.669746
**50**	O50	76.555580	56.778927	18.608038
**51**	O51	75.091141	53.393326	15.785621
**52**	O52	73.762405	53.107841	13.927160
**53**	O53	71.216599	55.469227	15.066699
**54**	O54	73.985184	55.221794	11.964080

Table S12. *Cont.*

**Center Number**		**Coordinates X**	**Y**	**Z**
**55**	O55	69.654419	54.304138	12.590844
**56**	O56	64.804657	54.115528	14.546799
**57**	O57	63.444073	55.139336	16.153366
**58**	H58	74.205437	59.236565	15.214181
**59**	H59	73.987083	57.662514	14.515414
**60**	H60	75.301384	56.631126	16.393984
**61**	H61	72.942703	58.395645	17.219118
**62**	H62	72.844963	56.752365	16.630651
**63**	H63	72.731003	56.842289	19.127794
**64**	H64	74.121300	55.926228	18.585255
**65**	H65	74.136131	58.882553	19.417593
**66**	H66	77.940201	57.214603	17.149847
**67**	H67	78.277039	54.794357	18.969000
**68**	H68	76.378342	53.582893	14.190787
**69**	H69	77.148209	51.292553	14.423738
**70**	H70	76.703094	51.266144	16.134674
**71**	H71	74.864174	51.008282	13.776711
**72**	H72	74.652657	50.443066	15.442946
**73**	H73	72.326202	51.570221	15.588340
**74**	H74	73.318489	51.903332	17.006685
**75**	H75	71.330200	53.473366	16.864843
**76**	H76	72.854332	54.336124	16.813795
**77**	H77	71.177406	53.507446	14.410799
**78**	H78	73.798515	55.085510	14.562951
**79**	H79	72.215599	54.200726	12.108788
**80**	H80	70.564377	57.116959	14.155984
**81**	H81	69.102150	56.243134	12.567011
**82**	H82	68.806351	54.588345	15.097375
**83**	H83	68.302986	56.243526	14.966803
**84**	H84	67.112289	53.953106	13.328830
**85**	H85	65.894119	55.469208	15.652419
**86**	H86	67.414223	53.844425	16.632124
**87**	H87	65.744827	52.462132	17.853495
**88**	H88	65.320801	54.150944	17.736780
**89**	H89	64.195221	51.915253	15.967487
**90**	H90	63.324837	52.791332	17.215122
**91**	H91	62.724388	52.630543	14.084874
**92**	H92	61.692696	53.292080	15.348522
**93**	H93	62.839752	54.854359	12.967046
**94**	H94	61.164028	54.385475	13.163515
**95**	H95	61.551945	56.767521	13.884328
**96**	H96	60.961933	55.778652	15.204452

Table S12. *Cont.*

**Center**		**Coordinates**		
**Number**		**X**	**Y**	**Z**
**97**	H97	63.885674	56.549133	14.696188
**98**	H98	62.873974	57.109608	16.008324
**99**	H99	67.203064	51.376106	16.156210
**100**	H100	66.191803	51.754406	14.763110
**101**	H101	67.867531	52.280300	14.810291
**102**	H102	65.365158	55.616989	12.667253
**103**	H103	66.250153	56.881695	13.505477
**104**	H104	66.937431	56.091225	12.086661
**105**	H105	80.020912	54.049232	17.347979
**106**	H106	79.854301	55.566021	16.459753
**107**	H107	80.478050	55.556786	18.106058
**108**	H108	79.875465	60.119370	19.605099
**109**	H109	78.484360	60.752888	20.488607
**110**	H110	79.416039	59.411205	21.158693
**111**	H111	75.308212	60.138939	13.122230
**112**	H112	76.660461	59.282764	12.392897
**113**	H113	75.047539	58.586739	12.335752
**114**	H114	78.017738	58.153805	14.497914
**115**	H115	76.642838	59.158169	15.949044
**116**	H116	75.523033	56.784077	20.588125
**117**	H117	74.695000	55.279148	12.599669
**118**	H118	68.838379	54.073872	12.171689
**119**	H119	79.758438	57.724892	18.678802
**120**	H120	76.266418	54.580666	17.525967
**121**	H121	78.466011	53.734921	15.584064
**122**	H122	71.565971	58.228203	12.321176
**123**	H123	72.984810	57.430222	11.456911
**124**	H124	76.379166	60.004974	19.545694
**125**	H125	78.953262	56.948433	20.031210

Table S13. CS II, Conformer 3. Gas phase energy and Boltzmann Populations −2695.554772 hartrees, 10.35%.

**Center**		**Coordinates**		
**Number**		**X**	**Y**	**Z**
**1**	C1	75.953598	57.063057	13.812674
**2**	C2	75.426163	58.435211	14.251992
**3**	C3	74.365150	58.326965	15.373464
**4**	C4	74.840797	57.659088	16.682007
**5**	C5	73.702652	57.554550	17.710096
**6**	C6	74.254082	57.017731	19.030720
**7**	C7	75.387062	57.937683	19.504711
**8**	C8	76.483459	58.014709	18.412474
**9**	C9	77.573914	58.996197	18.812452

Table S13. *Cont.*

Center		Coordinates		
Number		X	Y	Z
**10**	C10	78.880920	58.697781	18.731808
**11**	C11	79.328598	57.353378	18.191044
**12**	C12	78.178391	56.617889	17.468267
**13**	C13	78.459618	55.117535	17.225134
**14**	C14	77.204781	54.441353	16.687885
**15**	C15	77.068283	53.753441	15.539480
**16**	C16	75.756989	53.167011	15.057623
**17**	C17	75.723740	51.647251	15.002251
**18**	C18	74.230377	51.343060	15.051254
**19**	C19	73.609367	52.656746	15.558966
**20**	C20	72.662903	52.464542	16.762966
**21**	C21	72.063828	53.820873	17.159521
**22**	C22	71.376686	54.429192	15.936838
**23**	C23	72.399193	54.549763	14.790947
**24**	C24	71.731239	55.143181	13.541498
**25**	C25	71.003586	56.437725	13.909659
**26**	C26	70.181168	56.377422	15.199308
**27**	C27	68.725876	55.864189	15.019760
**28**	C28	67.996971	55.674377	16.367638
**29**	C29	66.442627	55.711189	16.340946
**30**	C30	65.680214	54.549713	15.637591
**31**	C31	65.613358	53.165478	16.338133
**32**	C32	64.715820	52.259880	15.481054
**33**	C33	65.245064	52.191475	14.042261
**34**	C34	65.329597	53.612152	13.447900
**35**	C35	66.003197	53.575569	12.058452
**36**	C36	65.992386	54.978973	11.438933
**37**	C37	64.549934	55.481812	11.364981
**38**	C38	63.920280	55.422688	12.763083
**39**	C39	66.971848	52.499977	16.622900
**40**	C40	65.891113	55.946072	17.759474
**41**	C41	71.130096	57.583588	13.211611
**42**	C42	79.694893	54.891003	16.331642
**43**	C43	79.975777	59.643696	19.184662
**44**	C44	74.867981	59.176327	13.027371
**45**	O45	77.294113	57.158817	13.641146
**46**	O46	75.256256	56.074280	13.607692
**47**	O47	76.525841	59.208363	14.720929
**48**	O48	75.895493	58.464928	17.221867
**49**	O49	75.923302	57.450855	20.713091
**50**	O50	77.008553	56.723019	18.278065
**51**	O51	74.687851	53.440918	15.947822

Table S13. *Cont.*

**Center Number**		**Coordinates X**	**Y**	**Z**
**52**	O52	72.907234	53.245979	14.501328
**53**	O53	70.873177	55.717907	16.264086
**54**	O54	72.722328	55.375439	12.549129
**55**	O55	68.683990	54.665966	14.277204
**56**	O56	66.122078	54.409481	14.287581
**57**	O57	64.025459	54.103516	13.299876
**58**	H58	73.979149	59.321365	15.600872
**59**	H59	73.511604	57.764778	14.991077
**60**	H60	75.214622	56.656525	16.468767
**61**	H61	73.246117	58.532116	17.870476
**62**	H62	72.914932	56.900299	17.338053
**63**	H63	73.461113	56.977806	19.777775
**64**	H64	74.612907	55.996113	18.896864
**65**	H65	74.995644	58.941727	19.677931
**66**	H66	77.985916	57.097965	16.506771
**67**	H67	78.657516	54.649422	18.190315
**68**	H68	75.527031	53.576706	14.073262
**69**	H69	76.212051	51.246864	14.113721
**70**	H70	76.215363	51.229820	15.881855
**71**	H71	73.825783	51.099361	14.068052
**72**	H72	74.014847	50.518879	15.731703
**73**	H73	71.876213	51.762897	16.482796
**74**	H74	73.220665	52.027733	17.591805
**75**	H75	71.349289	53.698250	17.973000
**76**	H76	72.849472	54.483631	17.522739
**77**	H77	70.562149	53.767059	15.645674
**78**	H78	73.208885	55.211002	15.103603
**79**	H79	71.001381	54.437382	13.144415
**80**	H80	70.085518	57.405399	15.552076
**81**	H81	68.190567	56.611374	14.431593
**82**	H82	68.381187	54.793835	16.874439
**83**	H83	68.314156	56.502270	17.003185
**84**	H84	66.181549	56.607357	15.776015
**85**	H85	64.648476	54.902561	15.584469
**86**	H86	65.110992	53.286251	17.298643
**87**	H87	64.654327	51.259136	15.908966
**88**	H88	63.696438	52.648930	15.474149
**89**	H89	66.233917	51.733128	14.013756
**90**	H90	64.588768	51.584393	13.417886
**91**	H91	67.024437	53.206612	12.162909
**92**	H92	65.463219	52.872925	11.422428
**93**	H93	66.599533	55.659176	12.038090

Table S13. *Cont.*

**Center**		**Coordinates**		
**Number**		**X**	**Y**	**Z**
**94**	H94	66.437294	54.955399	10.444072
**95**	H95	64.519585	56.500977	10.978215
**96**	H96	63.973000	54.865028	10.674691
**97**	H97	64.399254	56.140598	13.430352
**98**	H98	62.866722	55.697636	12.711704
**99**	H99	66.850166	51.444695	16.867376
**100**	H100	67.647331	52.565941	15.769910
**101**	H101	67.460464	52.954891	17.482483
**102**	H102	64.803383	56.024525	17.754681
**103**	H103	66.163727	55.133747	18.433708
**104**	H104	66.278557	56.870777	18.188116
**105**	H105	79.881187	53.827850	16.177412
**106**	H106	79.569595	55.358261	15.353928
**107**	H107	80.596901	55.303551	16.782633
**108**	H108	80.641327	59.880665	18.354164
**109**	H109	79.563263	60.578556	19.565691
**110**	H110	80.566849	59.185829	19.978315
**111**	H111	74.537544	60.182442	13.286615
**112**	H112	75.614304	59.270000	12.236962
**113**	H113	74.012161	58.647839	12.604788
**114**	H114	77.418152	58.069164	13.922536
**115**	H115	76.517365	59.122822	15.676013
**116**	H116	76.530197	56.766300	20.459064
**117**	H117	73.574524	55.376129	12.978751
**118**	H118	67.756134	54.444790	14.186909
**119**	H119	80.163574	57.502316	17.505919
**120**	H120	76.333183	54.553165	17.317169
**121**	H121	77.909241	53.606373	14.879135
**122**	H122	70.626244	58.487549	13.518216
**123**	H123	71.728600	57.632301	12.313748
**124**	H124	77.249321	59.946877	19.209078
**125**	H125	79.699501	56.758316	19.026217

Table S14. CS II, Conformer 4. Gas phase energy and Boltzmann Populations −2695.554695 hartrees, 9.54%.

**Center**		**Coordinates**		
**Number**		**X**	**Y**	**Z**
**1**	C1	76.033676	57.383125	13.781252
**2**	C2	75.513123	58.689163	14.398239
**3**	C3	74.450546	58.451412	15.496490
**4**	C4	74.890709	57.546078	16.667879
**5**	C5	73.756294	57.339962	17.684395
**6**	C6	74.279701	56.504890	18.850527

Table S14. *Cont.*

**Center**		**Coordinates**		
**Number**		**X**	**Y**	**Z**
**7**	C7	75.479843	57.229675	19.471411
**8**	C8	76.570908	57.464035	18.393890
**9**	C9	77.710930	58.287601	18.977356
**10**	C10	78.998810	57.918228	18.880869
**11**	C11	79.377014	56.658630	18.127438
**12**	C12	78.221214	56.186512	17.221451
**13**	C13	78.395760	54.756016	16.668289
**14**	C14	77.235130	54.437355	15.748568
**15**	C15	76.230659	53.594688	16.031992
**16**	C16	75.053246	53.370403	15.112164
**17**	C17	74.763718	51.913174	14.796431
**18**	C18	73.331985	52.000259	14.295819
**19**	C19	72.774040	53.227940	15.039486
**20**	C20	71.664230	52.865143	16.049200
**21**	C21	71.129921	54.146503	16.701122
**22**	C22	70.665367	55.094749	15.597732
**23**	C23	71.834908	55.369667	14.633391
**24**	C24	71.384911	56.311680	13.507080
**25**	C25	70.709641	57.550327	14.102010
**26**	C26	69.722168	57.273285	15.237571
**27**	C27	68.275154	56.956692	14.768746
**28**	C28	67.355217	56.516953	15.924918
**29**	C29	65.927757	56.099449	15.486600
**30**	C30	65.712227	54.561245	15.413899
**31**	C31	65.389610	53.821617	16.741716
**32**	C32	65.146118	52.345222	16.404375
**33**	C33	66.354340	51.764629	15.659117
**34**	C34	66.641418	52.594025	14.389535
**35**	C35	67.936386	52.079391	13.715927
**36**	C36	68.149109	52.748730	12.351790
**37**	C37	66.901718	52.561653	11.488951
**38**	C38	65.678650	53.081909	12.252862
**39**	C39	66.437218	53.978176	17.860979
**40**	C40	64.836403	56.815006	16.303955
**41**	C41	71.009590	58.809635	13.727809
**42**	C42	79.721169	54.55497	15.916602
**43**	C43	80.134300	58.694145	19.518984
**44**	C44	74.974480	59.597157	13.282463
**45**	O45	77.368217	57.515541	13.585978
**46**	O46	75.348984	56.404137	13.498984
**47**	O47	76.617546	59.376690	14.976639
**48**	O48	76.003944	58.171028	17.322170

Table S14. *Cont.*

**Center**		**Coordinates**		
**Number**		**X**	**Y**	**Z**
**49**	O49	75.993744	56.466976	20.538694
**50**	O50	77.022232	56.198605	17.994638
**51**	O51	73.850357	53.769039	15.739714
**52**	O52	72.274689	54.125427	14.086259
**53**	O53	70.225471	56.316288	16.174736
**54**	O54	72.528870	56.670815	12.747477
**55**	O55	68.247597	55.989277	13.741638
**56**	O56	66.824944	53.940895	14.753215
**57**	O57	65.569374	52.40958	13.506447
**58**	H58	74.118835	59.412346	15.891354
**59**	H59	73.568375	58.011536	15.031030
**60**	H60	75.190933	56.570217	16.283350
**61**	H61	73.391594	58.30188	18.046923
**62**	H62	72.906303	56.841328	17.220270
**63**	H63	73.494507	56.365578	19.593761
**64**	H64	74.563164	55.511242	18.498762
**65**	H65	75.159348	58.198162	19.859484
**66**	H66	78.115112	56.880459	16.384809
**67**	H67	78.371117	54.056980	17.505733
**68**	H68	75.179779	53.931900	14.185503
**69**	H69	75.449982	51.491997	14.061522
**70**	H70	74.810394	51.308647	15.703072
**71**	H71	73.310303	52.188026	13.221271
**72**	H72	72.752495	51.100494	14.503682
**73**	H73	70.867752	52.338943	15.523409
**74**	H74	72.071037	52.186733	16.799709
**75**	H75	70.304100	53.915417	17.373602
**76**	H76	71.908745	54.615509	17.303152
**77**	H77	69.847221	54.610542	15.066191
**78**	H78	72.654076	55.841000	15.176002
**79**	H79	70.676147	55.795132	12.859124
**80**	H80	69.646065	58.192841	15.819720
**81**	H81	67.865753	57.867004	14.327682
**82**	H82	67.834366	55.70195	16.461275
**83**	H83	67.302795	57.335728	16.643465
**84**	H84	65.790024	56.465202	14.467479
**85**	H85	64.838905	54.431057	14.771917
**86**	H86	64.449516	54.213387	17.132036
**87**	H87	64.946823	51.769756	17.308556
**88**	H88	64.255615	52.250576	15.781002
**89**	H89	67.241318	51.761444	16.292393
**90**	H90	66.162994	50.730595	15.370293

Table S14. *Cont.*

**Center**		**Coordinates**		
**Number**		**X**	**Y**	**Z**
**91**	H91	68.781860	52.268795	14.376613
**92**	H92	67.856628	50.999046	13.588325
**93**	H93	68.361015	53.808338	12.474131
**94**	H94	69.019196	52.319042	11.855204
**95**	H95	67.010834	53.091503	10.541988
**96**	H96	66.765282	51.506702	11.247858
**97**	H97	65.745613	54.160503	12.403852
**98**	H98	64.771439	52.899921	11.676616
**99**	H99	66.183136	53.361294	18.723112
**100**	H100	67.434578	53.688141	17.531101
**101**	H101	66.490913	55.006527	18.216316
**102**	H102	63.840401	56.527813	15.965155
**103**	H103	64.905777	56.586021	17.367163
**104**	H104	64.915176	57.897373	16.197678
**105**	H105	79.779587	53.552658	15.490972
**106**	H106	79.826050	55.269104	15.098864
**107**	H107	80.580139	54.672539	16.577034
**108**	H108	80.843956	59.023621	18.759602
**109**	H109	79.768318	59.575855	20.046188
**110**	H110	80.664955	58.066753	20.235689
**111**	H111	74.638756	60.556732	13.676093
**112**	H112	75.731712	59.799294	12.523561
**113**	H113	74.125641	59.133324	12.777552
**114**	H114	77.491257	58.386395	13.973159
**115**	H115	76.633018	59.113235	15.898235
**116**	H116	76.544518	55.810532	20.129492
**117**	H117	73.212715	56.037235	12.941386
**118**	H118	67.798798	55.217346	14.086383
**119**	H119	80.265282	56.852871	17.525585
**120**	H120	77.227638	54.961704	14.804208
**121**	H121	76.222046	53.058548	16.969322
**122**	H122	70.53685	59.661953	14.191514
**123**	H123	71.723228	59.007923	12.942192
**124**	H124	77.434311	59.177353	19.523373
**125**	H125	79.639038	55.887653	18.852779

Table S15. CS II, Conformer 5. Gas phase energy and Boltzmann Populations −2695.553772 hartrees, 3.59%.

**Center**		**Coordinates**		
**Number**		**X**	**Y**	**Z**
**1**	C1	75.890556	57.383163	13.848571
**2**	C2	75.523041	58.725010	14.495579
**3**	C3	74.394081	58.582329	15.544791
**4**	C4	74.705727	57.653561	16.738420
**5**	C5	73.506783	57.534351	17.692944
**6**	C6	73.911423	56.718246	18.920511
**7**	C7	75.121185	57.389496	19.584583
**8**	C8	76.278954	57.502151	18.560778
**9**	C9	77.456894	58.254303	19.159861
**10**	C10	78.720474	57.807774	19.071852
**11**	C11	79.030602	56.526138	18.322475
**12**	C12	77.839722	56.073250	17.448908
**13**	C13	77.950935	54.609932	16.963610
**14**	C14	76.651100	54.192635	16.287844
**15**	C15	76.487068	53.725315	15.036680
**16**	C16	75.139244	53.391056	14.430634
**17**	C17	74.930916	51.914566	14.125496
**18**	C18	73.411690	51.777874	14.118857
**19**	C19	72.927635	53.076397	14.786735
**20**	C20	71.898605	52.845150	15.913271
**21**	C21	71.445206	54.200317	16.474419
**22**	C22	70.919594	55.062267	15.325928
**23**	C23	72.015434	55.200214	14.253289
**24**	C24	71.509026	56.051193	13.079323
**25**	C25	70.943108	57.371632	13.606651
**26**	C26	70.040314	57.247353	14.836775
**27**	C27	68.546288	56.969433	14.515411
**28**	C28	67.703056	56.703598	15.778321
**29**	C29	66.229240	56.314533	15.494909
**30**	C30	65.944908	54.790417	15.613015
**31**	C31	65.687881	54.221287	17.035467
**32**	C32	65.356010	52.731239	16.886494
**33**	C33	66.481247	52.009453	16.135015
**34**	C34	66.715767	52.676933	14.763121
**35**	C35	67.938293	52.025154	14.073311
**36**	C36	68.087532	52.527111	12.631272
**37**	C37	66.777435	52.309639	11.874915
**38**	C38	65.632271	52.972111	12.649562
**39**	C39	66.821098	54.447716	18.055058
**40**	C40	65.235878	57.170998	16.301706
**41**	C41	71.267838	58.574757	13.093816
**42**	C42	79.190857	54.380352	16.077417

Table S15. *Cont.*

**Center**		**Coordinates**		
**Number**		**X**	**Y**	**Z**
**43**	C43	79.895775	58.516060	19.716465
**44**	C44	75.138634	59.726955	13.396097
**45**	O45	77.239693	57.318153	13.743106
**46**	O46	75.082405	56.547279	13.455462
**47**	O47	76.685822	59.251278	15.126298
**48**	O48	75.818993	58.214748	17.444395
**49**	O49	75.523903	56.642574	20.709105
**50**	O50	76.649414	56.193291	18.226366
**51**	O51	74.070511	53.651932	15.325438
**52**	O52	72.362175	53.891094	13.799306
**53**	O53	70.572411	56.350540	15.817247
**54**	O54	72.578491	56.275948	12.170374
**55**	O55	68.387306	55.906067	13.601954
**56**	O56	66.981392	54.045189	14.956956
**57**	O57	65.579178	52.449532	13.976109
**58**	H58	74.126137	59.569908	15.922248
**59**	H59	73.498810	58.210087	15.043915
**60**	H60	74.963829	56.658455	16.372948
**61**	H61	73.168892	58.524345	18.002089
**62**	H62	72.665146	57.062016	17.187468
**63**	H63	73.078613	56.660271	19.621618
**64**	H64	74.147942	55.694599	18.626266
**65**	H65	74.847755	58.393448	19.914183
**66**	H66	77.758316	56.725357	16.577114
**67**	H67	78.046387	53.968708	17.840874
**68**	H68	75.005074	53.978928	13.521594
**69**	H69	75.390411	51.611588	13.184563
**70**	H70	75.352516	51.303631	14.924659
**71**	H71	73.003792	51.705418	13.109824
**72**	H72	73.092583	50.904709	14.688554
**73**	H73	71.052826	52.291275	15.506478
**74**	H74	72.352501	52.233425	16.693485
**75**	H75	70.670380	54.060642	17.227890
**76**	H76	72.282166	54.695580	16.966860
**77**	H77	70.044395	54.566620	14.907831
**78**	H78	72.883850	55.693508	14.692658
**79**	H79	70.715492	55.519783	12.553537
**80**	H80	70.055725	58.217453	15.335706
**81**	H81	68.147797	57.852291	14.012828
**82**	H82	68.187317	55.926796	16.364782
**83**	H83	67.742393	57.595875	16.404175
**84**	H84	66.027954	56.572289	14.453582

Table S15. *Cont.*

**Center**		**Coordinates**		
**Number**		**X**	**Y**	**Z**
**85**	H85	65.023399	54.632710	15.049306
**86**	H86	64.796791	54.701962	17.441034
**87**	H87	65.196510	52.271259	17.861877
**88**	H88	64.420151	52.613987	16.337900
**89**	H89	67.409431	52.029671	16.705828
**90**	H90	66.224045	50.961254	15.978923
**91**	H91	68.833763	52.243092	14.654455
**92**	H92	67.802795	50.942841	14.074979
**93**	H93	68.354378	53.581249	12.618521
**94**	H94	68.902237	52.000763	12.133783
**95**	H95	66.845131	52.725513	10.869021
**96**	H96	66.579437	51.242809	11.763961
**97**	H97	65.755394	54.056072	12.672008
**98**	H98	64.681198	52.773094	12.155547
**99**	H99	66.604378	53.945580	18.998047
**100**	H100	67.777679	54.071033	17.693588
**101**	H101	66.946465	55.505211	18.284283
**102**	H102	64.205368	56.899281	16.070396
**103**	H103	65.377174	57.058151	17.376368
**104**	H104	65.352264	58.229446	16.066944
**105**	H105	79.256744	53.341927	15.751645
**106**	H106	79.167534	55.013123	15.189141
**107**	H107	80.113403	54.598019	16.614763
**108**	H108	80.629158	58.800526	18.961435
**109**	H109	79.579620	59.419514	20.239082
**110**	H110	80.383125	57.860085	20.438337
**111**	H111	75.935028	59.843616	12.659428
**112**	H112	74.929466	60.713295	13.810926
**113**	H113	74.246414	59.399727	12.860421
**114**	H114	77.465958	58.144314	14.178555
**115**	H115	76.609421	59.017864	16.053257
**116**	H116	76.057465	55.937691	20.362774
**117**	H117	73.399284	56.157944	12.643105
**118**	H118	67.952484	55.191284	14.066560
**119**	H119	79.912201	56.679073	17.699427
**120**	H120	75.771072	54.306110	16.904930
**121**	H121	77.334091	53.586613	14.382244
**122**	H122	70.872780	59.488808	13.510451
**123**	H123	71.921852	58.664364	12.238818
**124**	H124	77.228973	59.159286	19.703493
**125**	H125	79.283089	55.758308	19.054516

© 2014 by the authors; licensee MDPI, Basel, Switzerland. This article is an open access article distributed under the terms and conditions of the Creative Commons Attribution license (http://creativecommons.org/licenses/by/3.0/).
